# The cellular correlates and adolescent reorganisation of cortical myelination networks in the common marmoset

**DOI:** 10.1038/s42003-026-10276-y

**Published:** 2026-05-19

**Authors:** E. D. Hutchings, S. J. Sawiak, R. L. Smith, R. A. I. Bethlehem, A. C. Roberts, E. T. Bullmore

**Affiliations:** 1 Department of Psychiatry, University of Cambridge, Cambridge, UK; 2 Department of Physiology, Development and Neuroscience, University of Cambridge, Cambridge, UK; 3 Human Genetics Branch, National Institute of Mental Health, Bethesda, MD USA; 4 Department of Psychology, University of Cambridge, Cambridge, UK; 5 School of Academic Psychiatry, Institute of Psychiatry, Psychology & Neuroscience, King’s College London, London, UK

**Keywords:** Neuroscience, Developmental biology, Development of the nervous system

## Abstract

Structural similarity provides a powerful framework for measuring coordinated macro- and micro-structural variation across the cortex of a single brain. Similarity networks derived from myelin-sensitive MRI sequences undergo marked reorganisation during adolescence, linked to adversity exposure and behavioural outcomes in humans and rodents. However, the cellular mechanisms of MRI similarity and its development in non-human primate cortex remain unexplored. Here, we use myelin-sensitive T1w/T2w ratio images from a cross-sectional sample of 446 common marmosets (aged 0.62 to 12.75 years) to estimate MIND (Morphometric INverse Divergence) as a measure of myelination similarity in individual animals. We find that MIND metrics of myelination similarity are highly correlated with spatial gene expression of myelin basic protein (*MBP*) and other genes enriched in glutamatergic neurons and PV+ and VIP+ interneurons, reflecting the activity dependence of myelination. Across adolescence, network phenotypes accurately predict animal age and reproducibly capture an axis of myeloarchitectonic maturation spanning primary to transmodal association cortices, consistent with patterns observed in humans. Similarity mapping is a biologically validated and technically reliable measure of cortical myelination networks that can advance our understanding of phylogenetically conserved patterns of myelination network development in the marmoset cortex.

## Abstract (web summary)

Network modelling of myelin-sensitive MRI data in primates reveals that cortical myelin architecture is coupled to neuronal gene expression and undergoes a programme of adolescent reorganisation that mirrors changes seen in humans.

## Introduction

Network science has significantly advanced our understanding of brain structure^1–3^. Magnetic resonance imaging (MRI) has been a key driver of this progress because it has enabled the collection of whole brain images at the millimetre scale in vivo^1^. Analysis of MRI images allows quantification of brain structure both at the macro-scale, through metrics such as cortical thickness, surface area, and mean curvature; and at the micro-scale, below the minimal resolution of single voxels, using sequences such as diffusion imaging and magnetisation transfer^4^. Recent technical advances in structural similarity have permitted the integration of these MRI-derived structural features into a single network representation, with edges between nodes defined by the statistical relationships between the MRI features measured in pairs of regional nodes^5,6^. Similarity networks therefore represent spatially patterned structural variation across the cortex (the architectome), rather than directly measuring connectivity (the connectome), as is the case in diffusion tensor imaging (DTI) tractography. However, areas that are structurally similar are more likely to be axonally connected due to the network formation principle of homophilic attachment, or “like attracts like”^6,7^. Accordingly, the architectome represented by MRI-derived structural similarity networks has been correlated empirically with the connectome represented by axonal tract tracing data in the macaque^8–10^ and rat^11^ cortex.

A recently developed method for estimating structural similarity networks from MRI data is morphometric inverse divergence (MIND), which uses Kullback-Leibler divergence to quantify the similarity between a pair of cortical areas in terms of their voxel- or vertex-wise distributions of one or more structural MRI features^8^. MIND and adjacent methods are technically robust and reliable estimators^8,12^ of heritable phenotypes^8^, associated with similarity in gene expression^8,9,11^, developmentally sensitive^5,8,11^, and altered in psychiatric disorders^5,13,14^.

Animal models can enhance our understanding of the biological underpinnings of structural similarity networks and how they are reorganised during development and disease^10,11^. The common marmoset (*Callithrix jacchus*), a New World primate, represents a key rung on the translational ladder between rodent and human neuroscience, combining the tractability of rodent research with the translational relevance of primate research^15^. Like rodents, marmosets have an accelerated lifespan compared to humans, reaching adulthood at 18-20 months^16,17^, which facilitates longitudinal MRI studies that collect repeated measures over the lifespan. Like humans, marmosets show prolonged postnatal development in the context of familial nurturing^18–20^, and protracted development of association cortex^17^, particularly those areas involved in social cognition^21^.

Representations of the marmoset structural connectome generated from “gold standard” tract tracing data^22,23^ have revealed topological similarities to the human brain, with a rich club of highly interconnected hubs and exponentially distributed connection distances or wiring costs^24,25^. These topological parallels between human and marmoset brain networks, combined with the suitability of marmosets for MRI^26^ and the drive towards greater use of non-invasive imaging for primate research as part of the 3Rs in neuroscience^27^, have motivated efforts to generate MRI structural networks in the common marmoset, using DTI tractography^28^ and structural covariance^29^. However, there are problems with both of these approaches. DTI tractography suffers from a high false positive rate, i.e., identifying tracts between cortical areas that are not validated by comparable tract tracing data^30^; and a bias against long-range connections, i.e., failing to identify tracts between distantly separated cortical areas that are demonstrated by tract tracing data^31^. Whereas, structural covariance network analysis is constrained to estimate a network representing between-subject covariance in a single MRI-derived feature, typically from a dataset of *N* ~100 subjects, and therefore cannot provide the single-brain network analysis that is required for MRI studies of brain network development. We anticipated that similarity network analysis of MRI data in the common marmoset could circumvent these issues and provide a robust methodological platform for measuring the neurodevelopmental trajectories of primate brain network maturation.

To investigate the biological significance of structural similarity network analysis in the common marmoset, we focused on a myelin-sensitive MRI parameter, the T1w/T2w ratio^32^. Myelin is important to normative brain function^33^, shows characteristic patterns of variation across the cortex^34,35^, and a clear programme of developmental changes in humans^36^. However, developmental changes in myeloarchitecture of the marmoset cortex have not previously been investigated. To do this, we estimated MIND networks from an open cross-sectional dataset containing T1-weighted (T1w) and T2-weighted (T2w) MRI images from 446 animals spanning pre-puberty to senescence (0.62–12.75 years)^28^. Specifically, we estimated the ratio between T1w and T2w images to yield parameter maps with enhanced myelin-sensitive contrast^32^ and estimated similarity networks from parcellated voxel-wise T1w/T2w ratio images using MIND^8^.

We used these similarity networks to test two key hypotheses grounded in gene expression and development of the marmoset brain. First, assuming that T1w/T2w MIND network metrics are representative at the macro scale of cortical (co-)variation in microscopic myeloarchitectonics, they should be significantly co-located with molecular and cellular markers of myeloarchitecture. To test this, we benchmarked T1w/T2w MIND networks against two transcriptomic datasets: (i) spatially-resolved in-situ hybridisation images of myelin basic protein (*MBP*) messenger RNA (mRNA) in an infant and adult marmoset^37,38^; and (ii) whole genome single nucleus RNA sequencing data from 12 cortical regions in 6 adult marmosets^39^. Second, assuming that T1w/T2w MIND networks can thus be validated as cortical myelination networks, we predicted: (i) that T1w/T2w MIND networks would show age-related changes during adolescence which are consistent across individuals and predictive of chronological age; and (ii) that the cortical patterning of network changes is conserved across species, such that cortical areas occupying a similar position in the hierarchy from low-level primary sensory areas to higher-order transmodal association areas^40–42^ would show similar trajectories of myelination network development, as has been observed in humans^43^.

## Results

### Estimation and anatomical validation of T1w/T2w similarity networks

**Fig. 1:**
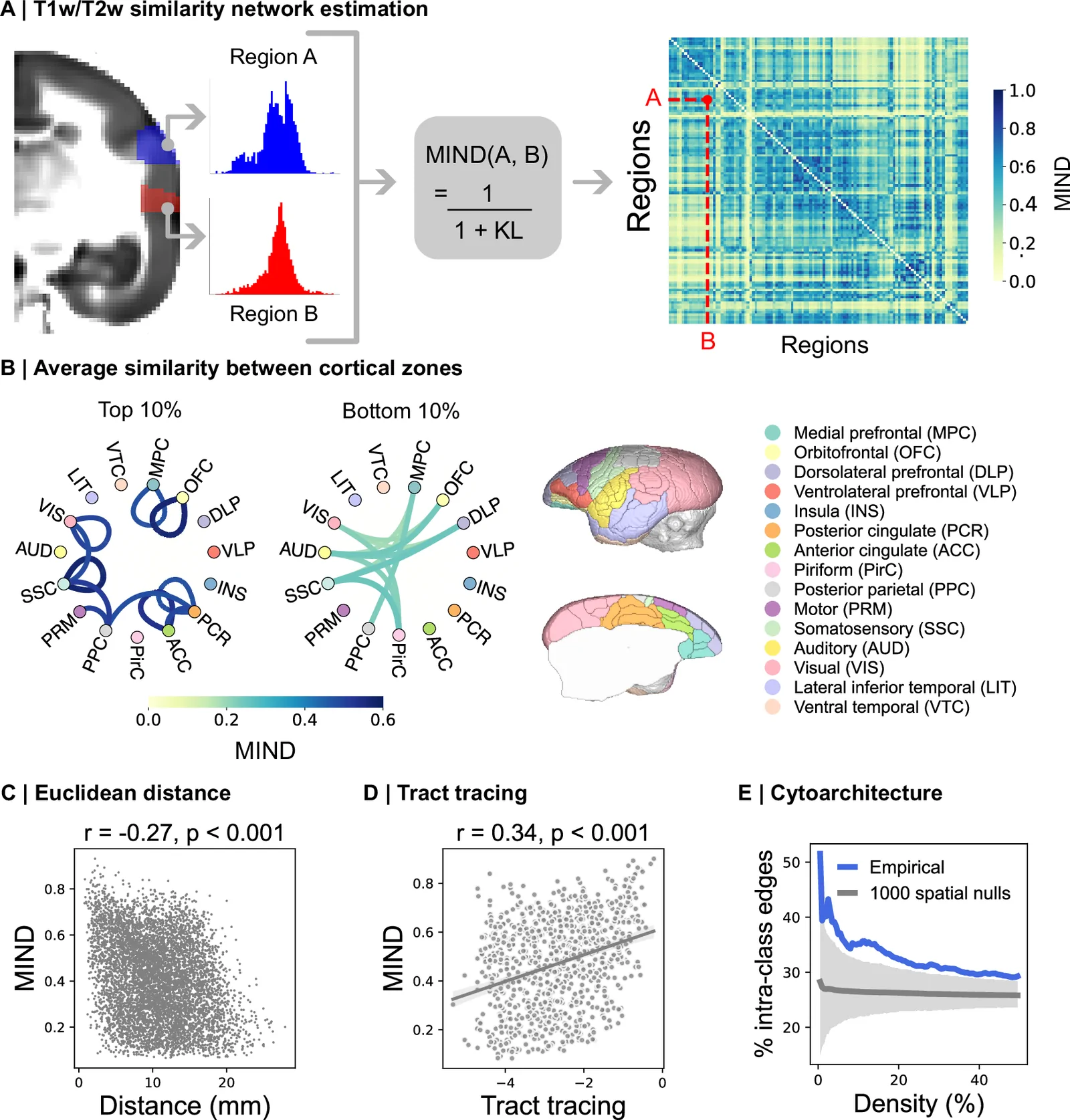
T1w/T2w similarity reflects axonal connectivity and cytoarchitectonic similarity. **A** Similarity between pairs of cortical areal T1w/T2w voxel distributions was estimated using morphometric inverse divergence (MIND). The average adult (*N* = 340 animals) T1w/T2w similarity network is shown on the right of the panel. **B** Mean similarity between *N* = 15 cortical zones. Top and bottom 10% of mean between-zone similarity is shown. **C** T1w/T2w similarity decays with physical distance between cortical areas across 26,335 edges. **D** T1w/T2w similarity is significantly correlated with axonal connectivity across 1485 edges. **E** The proportion of edges (*N* = 26,335) between regions of the same cytoarchitectonic class (*N* = 6 classes) increases as the network is thresholded to lower densities. The empirical network is shown in blue. The mean of 1000 spatial autocorrelation-preserving null networks is shown in grey, with shading indicating the 95% confidence interval.

Morphometric inverse divergence (MIND) is essentially the inverse of the Kullback-Leibler (KL) divergence^44^, which quantifies the similarity between each pair of cortical areas in terms of their voxel-wise T1w/T2w distributions (Fig. 1A, Supplementary Fig. 1). Several methods have been used to estimate KL divergence in the context of MRI structural similarity networks (Supplementary Methods), though these methods have not been formally compared. We therefore benchmarked two estimators of KL divergence, based on either global or local comparisons of distribution density, and variable parameters for each of these estimators (Supplementary Table 1 and Supplementary Figs. 2 and 3), on a set of biologically informed performance criteria and an age prediction task. While both algorithms produced similar MIND networks, the local estimator out-performed the global estimator across all performance criteria and the age prediction task (Supplementary Fig. 4). Our subsequent analyses, therefore, used the local KL estimator, as previously implemented^8^, to construct MIND networks from voxel-level data.

The average adult (*N* = 340, mean age = 4.3 years) T1w/T2w similarity network displayed high average similarity within anatomically and functionally defined cortical zones^45^ and strong dis-similarity between primary sensory and prefrontal association cortical zones (Fig. 1B). For two example primary sensory and prefrontal association regions, seed-based maps of MIND similarity to the rest of the cortex are visualised in Supplementary Fig. 5. The edges of the mean adult network recapitulated biological relationships with Euclidean distance, axonal connectivity, and cytoarchitecture previously demonstrated for structural similarity networks in other non-human species^8,9,11^. MIND similarity was negatively correlated with the Euclidean distance between regions (Spearman *r* = −0.27, *p*_rand_ < 0.001, Fig. 1C). Using retrograde tract tracing data as a “gold standard” estimate of the average adult marmoset connectome^22^, we observed a significant positive correlation between T1w/T2w similarity and axonal connectivity (Spearman *r* = 0.34, *p*_variogram_ < 0.001, Fig. 1D), reflecting homophily in cortical connectivity^6^. Using a cytoarchitectonic class assignment map^46^ (Supplementary Fig. 6B), the proportion of edges between areas of the same cortical class increased as the network was thresholded at progressively lower densities and was consistently greater than the proportion of intra-class edges on average over 1000 spatially constrained null model networks (Fig. 1E). This suggests that high values of T1w/T2w MIND represent the high cytoarchitectonic and myeloarchitectonic similarity between cortical areas of the same histologically defined class.

### T1w/T2w similarity networks align with molecular and cellular transcriptomic markers of myeloarchitecture

**Fig. 2:**
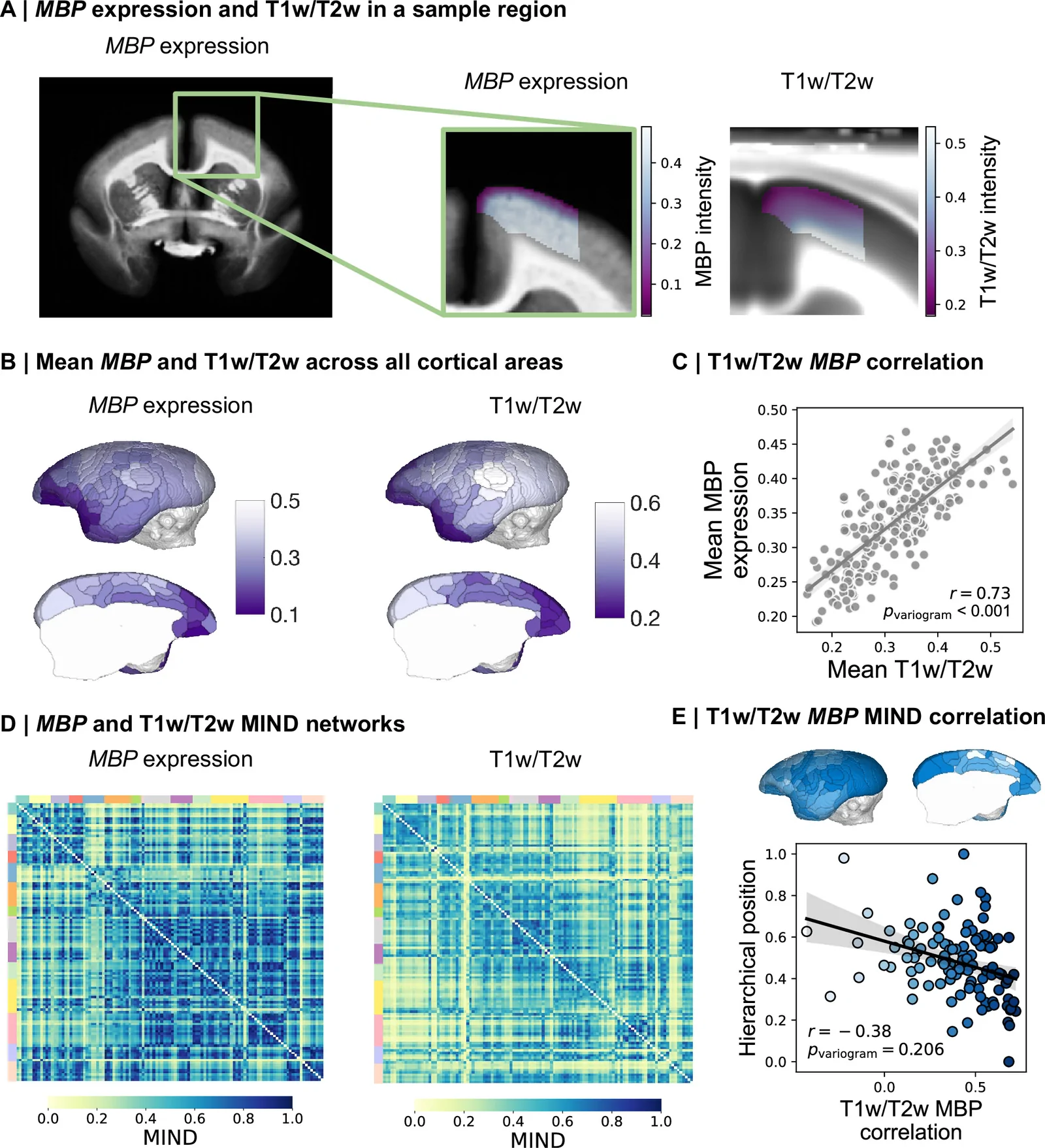
T1w/T2w similarity aligns with inter-areal similarity of myelin basic protein (MBP) gene expression. **A** Qualitative comparison of cortical myelination in MBP mRNA and T1w/T2w images in an example region, the left primary motor cortex. For T1w/T2w, the colour scale is capped at the 90^th^ percentile of intracortical values to improve visualisation. **B** Mean *MBP* expression and mean T1w/T2w per cortical area. **C** Regional mean *MBP* expression and T1w/T2w were significantly correlated across all 230 cortical areas. **D** Adult MIND networks estimating inter-areal similarity of *MBP* expression and T1w/T2w, grouped into 15 cortical zones. **E** Regional correlations between the MIND network edge weights linking each cortical area to the rest of the brain in *MBP* and T1w/T2w MIND networks. The correlation (Spearman *r*) between MBP MIND and T1w/T2w MIND edge weights is shown for each area (*N* = 230) on a cortical map (top panel). The strength of positive correlation was greater for specialised sensory and motor areas positioned lower in the cortical hierarchy (bottom panel).

Given the strong correlation of T1w/T2w MRI with myelin^32,47^, we hypothesised that T1w/T2w similarity would mirror similarity in a molecular transcriptomic marker of myeloarchitecture. To test this hypothesis, we downloaded spatially resolved images of myelin basic protein (*MBP*) messenger RNA (mRNA) expression from the Marmoset Gene Atlas^37,38^, measured using in-situ hybridisation, and registered these images to MRI space (Supplementary Fig. 7). The resulting *MBP* mRNA image volume was compared with a mean T1w/T2w volume formed by averaging T1w/T2w images from *N* = 16 age- and sex-matched animals. Both modalities showed a drop-off in myelin-related signal in superficial layers of cortex, though this was more obvious in the *MBP* mRNA image (Fig. 2A). Regionally parcellated cortical maps of mean *MBP* expression and mean T1w/T2w (Fig. 2B) were positively correlated (Spearman *r* = 0.73, *p*_variogram_ < 0.001, Fig. 2C). Standard deviation and skewness, quantifying the shape of each regional distribution, were less strongly, though still significantly, correlated between *MBP* mRNA and T1w/T2w MRI images (Supplementary Fig. 8).

Given the high correspondence between T1w/T2w and *MBP* expression, we further hypothesised that *MBP* MIND networks would show a highly similar organisation to T1w/T2w MIND networks. Since each cortical area in the *MBP* mRNA volume contains a distribution of voxel values of *MBP* expression, we could estimate the *MBP* MIND network using identical methods and a commensurate cortical parcellation as was used to estimate T1w/T2w MIND networks (Fig. 2D). Across all 26,335 edges, T1w/T2w and *MBP* similarity were significantly correlated (Spearman *r* = 0.42, *p*_variogram_ < 0.001), and the strength of correlation was weaker (non-significantly) for regions highest in the cortical hierarchy (Spearman *r* = −0.38, *p*_variogram_ = 0.208, Fig. 2E). This was not driven exclusively by cross-modal similarity in signal intensity distributions, which displayed a distinct cortical pattern (Supplementary Fig. 9).

**Fig. 3:**
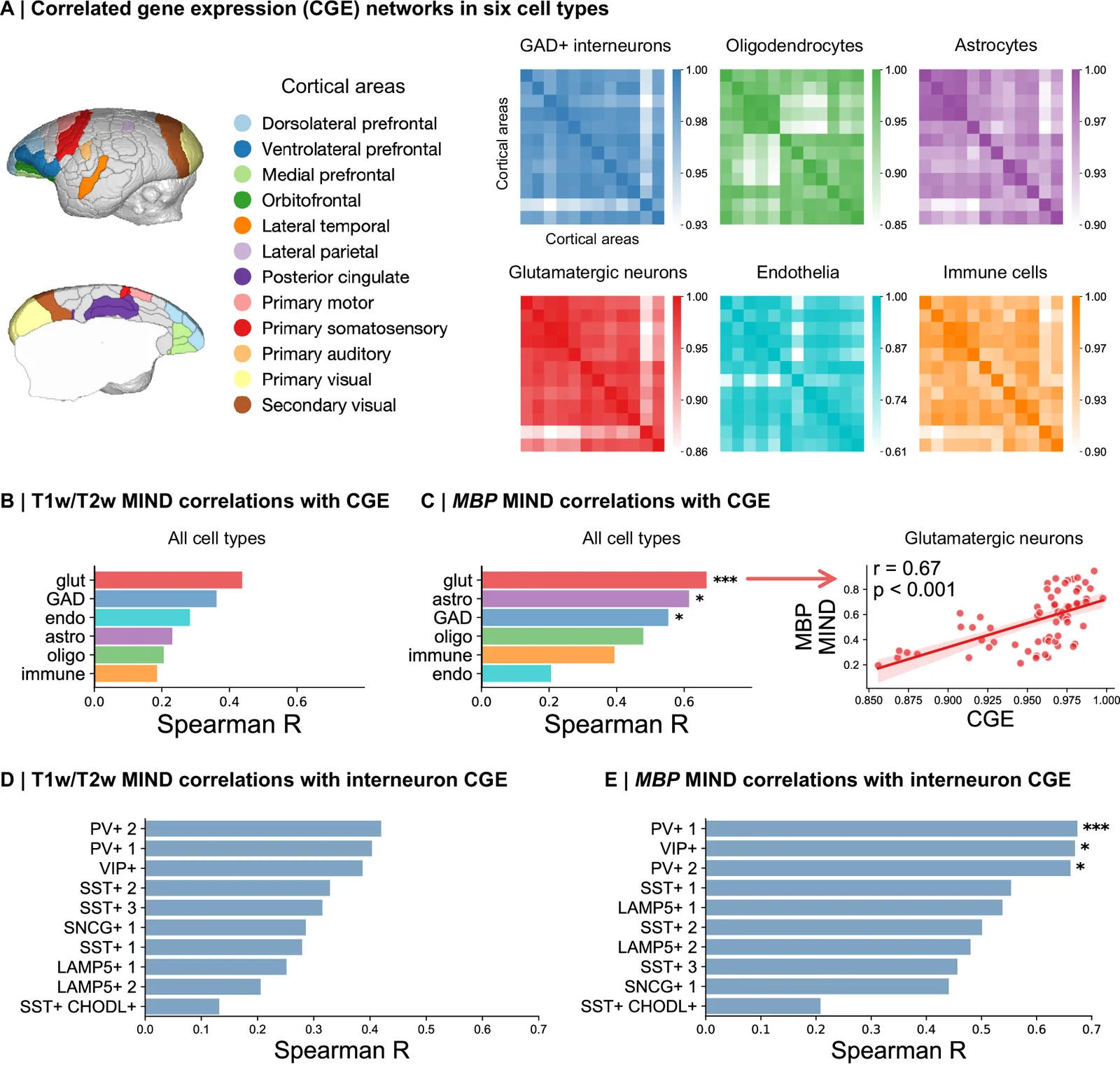
Myeloarchitectonic similarity is associated with co-expression of genes in glutamatergic neurons and PV+ and VIP+ interneurons. **A** Cell type-specific correlated gene expression (CGE) matrices estimated using single nucleus RNA sequencing data from 12 cortical areas^39^. In the following plots, bars represent the correlation (Spearman’s *r*) between myeloarchitectonic (T1w/T2w or *MBP* expression) similarity network edges and whole genome co-expression network edges (*N* edges = 66 in all panels). Asterisks indicate the level of significance after comparison with spatial autocorrelation preserving nulls and Bonferroni correction for multiple (six) comparisons: *p < 0.05, **p < 0.01, ***p < 0.001. **B** T1w/T2w MIND is most strongly associated with whole genome co-expression by glutamatergic neurons. **C** *MBP* MIND was significantly correlated with whole genome co-expression by neurons and astrocytes, most strongly with glutamatergic neurons. **D** T1w/T2w MIND was correlated with gene co-expression in PV+ and VIP+ interneurons, though no correlations were significant after correction for multiple comparisons. **E** *MBP* MIND was strongly and significantly correlated with gene co-expression in PV+ and VIP+ interneurons.

Studies using bulk transcriptomic data on human cortex have found that: (1) mean T1w/T2w correlates strongly with the first principal component of gene expression^34^; and (2) macroscale structural similarity is correlated with gene co-expression^8^. We therefore hypothesised that T1w/T2w MIND similarity would be correlated with gene co-expression. To assess this hypothesis, we used single nucleus RNA sequencing data^39^ from 12 cortical areas (Fig. 3A and Supplementary Table 2). The use of single cell transcriptomic data allowed us to assess the relationship between T1w/T2w MIND and gene co-expression within distinct cell types while avoiding cell type compositional differences as a potential confound^48^. We estimated whole genome co-expression matrices for 6 cell types (Fig. 3A) and correlated these with a matched average adult T1w/T2w MIND network (*N* = 340, mean age = 4.3 years). The cell type-specific correlation between whole genome co-expression and T1w/T2w MIND similarity was strongest for glutamatergic neurons (Spearman *r* = 0.44; uncorrected *p* = 0.049); although no cell types remained significant after Bonferroni correction for multiple (6) comparisons (Fig. 3B). When we repeated this analysis using *MBP* MIND edge weights instead of T1w/T2w MIND edge weights, we found much stronger relationships with cell type-specific gene co-expression. Inter-areal co-expression by glutamatergic neurons was most strongly correlated with *MBP* MIND (Spearman *r* = 0.67, *p*_FWER_ < 0.001). Whole genome co-expression was significantly correlated with *MBP* MIND in glutamatergic neurons, interneurons, and astrocytes after Bonferroni correction for multiple comparisons (Fig. 3C).

Cortical cell types contain considerable heterogeneity^39,49^. We therefore hypothesised that there may be cell subtypes driving the relationship between gene co-expression and MIND similarity. We elected to test this relationship within GAD+ interneurons as previous work has indicated that markers of distinct interneuron subtypes are differentially correlated with T1w/T2w and other measures of cortical hierarchy in diverse species, with parvalbumin (PV+) interneurons being particularly strongly co-located with cortical hierarchy^34,50–53^. We therefore estimated the correlations between interneuronal subtype-specific whole genome co-expression and T1w/T2w MIND edge weights for a set of 10 interneuron subtypes defined a priori by hierarchical clustering^39^ (Supplementary Fig. 10). We observed that both T1w/T2w MIND and *MBP* MIND were most strongly correlated with whole genome co-expression by PV+ and vasoactive intestinal peptide (VIP+) interneuronal subtypes (Fig. 3D, E). Correlations between T1w/T2w MIND and genome co-expression by type 2 PV+ interneurons (Spearman *r* = 0.43, uncorrected *p*_variogram_ = 0.049), type 1 PV+ interneurons (Spearman *r* = 0.40, uncorrected *p*_variogram_ = 0.085), and VIP+ interneurons (Spearman *r* = 0.39, uncorrected *p*_variogram_ = 0.061) were non-significant after multiple comparisons correction. However, correlations between *MBP* MIND similarity and whole genome co-expression by type 1 PV+, type 2 PV+, and VIP+ interneurons remained significant after correction for multiple comparisons. Furthermore, we observed non-significant associations between T1w/T2w and *MBP* MIND and gene co-expression in two oligodendrocyte subtypes (**Supplementary Methods**).

### Age-related changes in cortical T1w/T2w

**Fig. 4:**
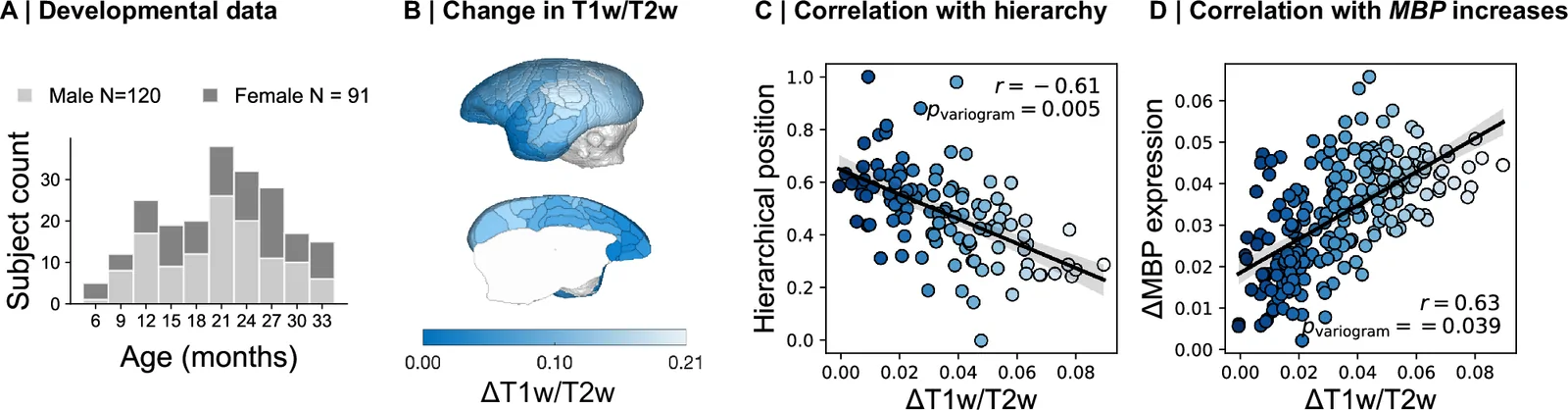
Marmosets show conserved adolescent increases in myelination. **A** *N* = 211 animals were used for developmental analysis, ranging from 7 months (pre-puberty) to 3 years (mature adulthood). **B** Rate of change in mean T1w/T2w for each region (*N* = 230) during this period, assessed using multiple linear regressions. Left hemisphere results are shown, though rates of change were bilaterally similar. **C** Rate of change in mean T1w/T2w was negatively associated with a region’s position in the cortical hierarchy across the 115 left hemispheric regions for which hierarchical position data were present. **D** Rate of change in mean T1w/T2w is significantly correlated with change in mean *MBP* expression across 230 areas.

We modelled age-related changes in a subset of *N* = 211 marmosets with ages ranging from 7 to 36 months (pre-puberty to mature adulthood; Fig. 4A). We used multiple linear regressions to estimate the linear slope of mean T1w/T2w change over this age range (Fig. 4B). We used linear models because they are easily interpretable and allowed for a direct comparison with linear age-related changes in *MBP* for which only two timepoints were available. Supplementary non-linear analyses revealed that mean T1w/T2w values plateau before 3 years of age, motivating the use of 3 years as an upper age range for linear analyses (Supplementary Fig. 11). Corroborating previous findings in humans^54,55^, we found that primary sensory regions lower in the cortical hierarchy showed significantly greater age-related increases in T1w/T2w, while regions higher in the hierarchy showed significantly smaller age-related increases in T1w/T2w (Spearman *r* = −0.61, *p*_variogram_ = 0.005, Fig. 4C). Furthermore, β_age_ coefficients were positively correlated with pre-pubertal (< 1 year) mean T1w/T2w, which would support a “rich get richer” pattern of myelination, though this trend did not remain significant when accounting for spatial autocorrelation (Spearman *r* = 0.66, *p*_variogram_ = 0.079, Supplementary Fig. 12A). Coefficients of age-related change were robust to the inclusion of covariates acting as additional T1w/T2w image normalisations (Supplementary Fig. 12B). Furthermore, these results were not unique to MRI; rate of change in mean T1w/T2w was highly correlated with the rate of change in mean *MBP* expression between a 6-month-old (pre-pubertal) male and a 4-year-old (mature adult) male animal (Spearman *r* = 0.63, *p*_variogram_ = 0.039, Fig. 4D).

### Developmental changes in myelination similarity

**Fig. 5:**
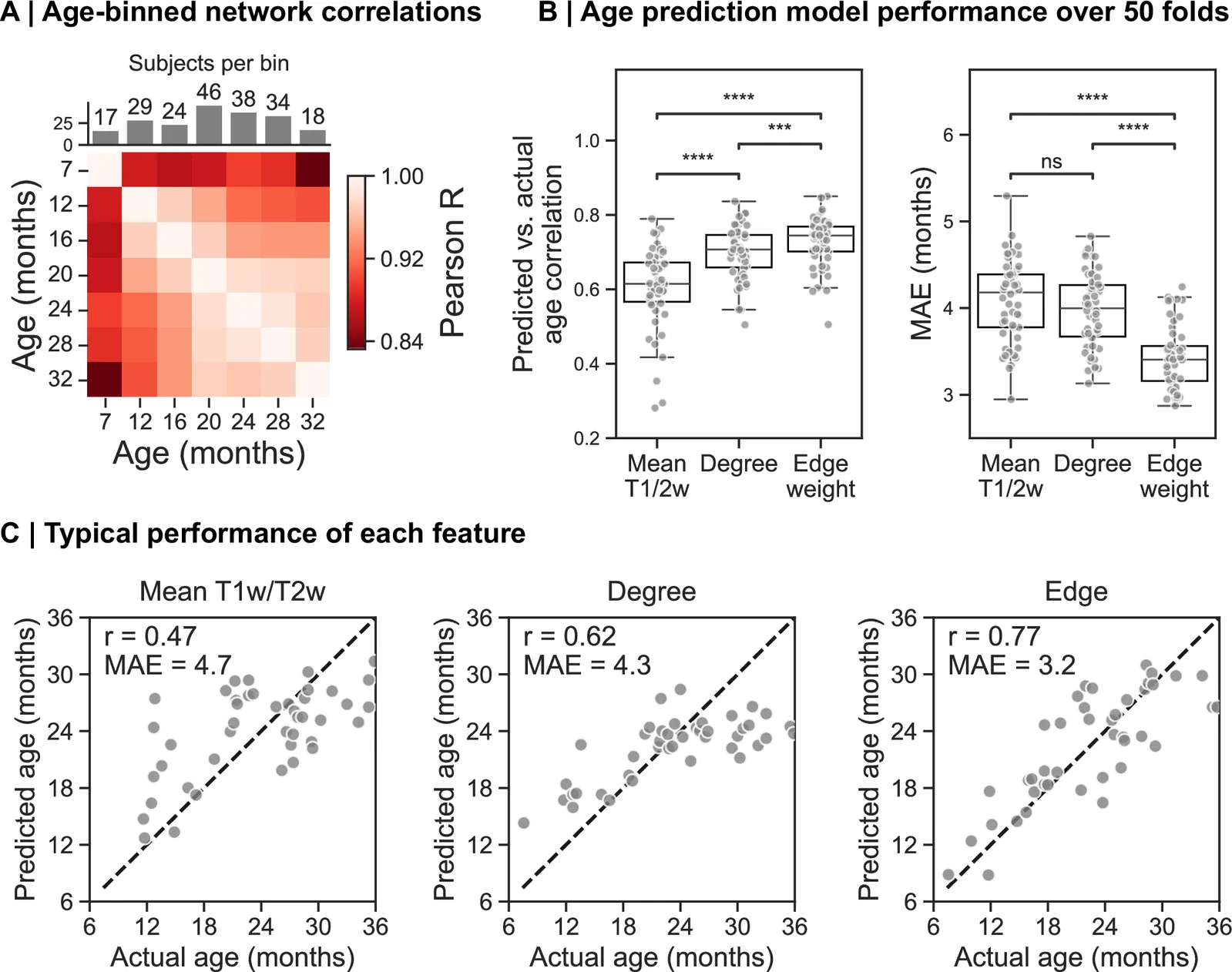
T1w/T2w MIND network edges and nodal degrees predict age more accurately than regional mean T1w/T2w. **A** Networks averaged into *N* = 7 age bins become decreasingly correlated as the age between bins  increases. *N* = 26,335 edges were used in each correlation (Spearman *r*). Correlations remained high after 20 months, indicating that there was relatively little change in T1w/T2w similarity beyond this age. **B** Performance of age prediction models trained on mean T1w/T2w and network features (degree and edge weights) over 50 splits of the data. Performance in each test fold was quantified as the mean absolute error (MAE) and partial correlation between *N* = 42 or 43 actual and predicted animal ages. Asterisks indicate significance of 3 paired Wilcoxon signed-rank tests: *p < 0.05, **p < 0.01, ***p < 0.001, ****p < 0.0001. **C** Typical model performance for each predictor. Performance of the model with the median partial R across 50 folds is shown here.

We hypothesised that T1w/T2w MIND would also show age-related changes representative of the inter-areally coordinated maturation of cortical myeloarchitecture. To qualitatively assess whether T1w/T2w MIND networks showed age-related changes, we assigned individual networks for animals aged <3 years to one of 7 age-defined bins (each spanning ≥4 months), estimated the mean network for each age bin by averaging across edges for animals within that bin, and computed the correlations between all pairs of averaged networks (Fig. 5A). Correlations between networks progressively decreased as the separation in age between them increased, suggesting that there were indeed graded network changes occurring during development. After 20 months, roughly corresponding to the termination of adolescence^17^, correlations between networks were more robust to variation in the age difference between them, indicating a slowing or termination of developmental change in T1w/T2w MIND. This maturational plateau was consistently found to begin between 18 and 21 months in sensitivity analyses using bin sizes of 3 and 6 months (Supplementary Fig. 13).

To assess the extent to which T1w/T2w MIND networks capture developmental changes which are chronologically consistent across individuals, we used regression models to predict subject age. Models were trained to predict age from an individual scan using one of three distinct sets of features: i) regional mean T1w/T2w signal itself; ii) MIND networks summarised by the weighted degree of each node; and iii) all edge weights in the MIND network. We reasoned that if models trained using similarity network metrics (ii and iii) provided more accurate age predictions than a non-network metric (i), this would suggest that similarity networks are more sensitive to the subtle biological changes occurring during adolescent brain development, prioritising their adoption in future neurodevelopmental MRI studies. We measured accuracy of age prediction using the partial correlation between predicted and true age, corrected for sex and estimated total intracranial volume (eTIV), and the mean absolute error (MAE) between predicted and true ages. Over 50 splits of the data, T1w/T2w MIND network features out-performed regional mean T1w/T2w in predicting age (Fig. 5B), indicated by higher partial correlations and lower MAE. Example model predictions (Fig. 5C) illustrate a plateau in age predictions around 2 years, most evident in the degree-based prediction. MIND edge weights and nodal degree more accurately predicted age than did regional mean T1w/T2w, reinforcing the added value of MIND network metrics in characterising intracortical maturation of myelin.

To identify specific age-related changes in nodal degree and edge weights of T1w/T2w MIND networks that were driving the superior predictive performance of MIND network features (Fig. 5), we used multiple linear regression. At the nodal level, 52/230 regions showed significant linear age-related changes in nodal degree after correction for multiple comparisons (Supplementary Fig. 14A). Anterior temporal, mainly auditory, and some frontal areas showed significant degree decreases, reflecting an increasingly differentiated myeloarchitectonic structure relative to the rest of the cortex (Supplementary Fig. 14B). Conversely, some somatosensory, primary motor, and visual areas showed significant degree increases, reflecting myeloarchitectonic convergence with the rest of the cortex (Supplementary Fig. 14B). This pattern of changes was robust to the addition of eTIV as a covariate (Supplementary Fig. 14C).

**Fig. 6:**
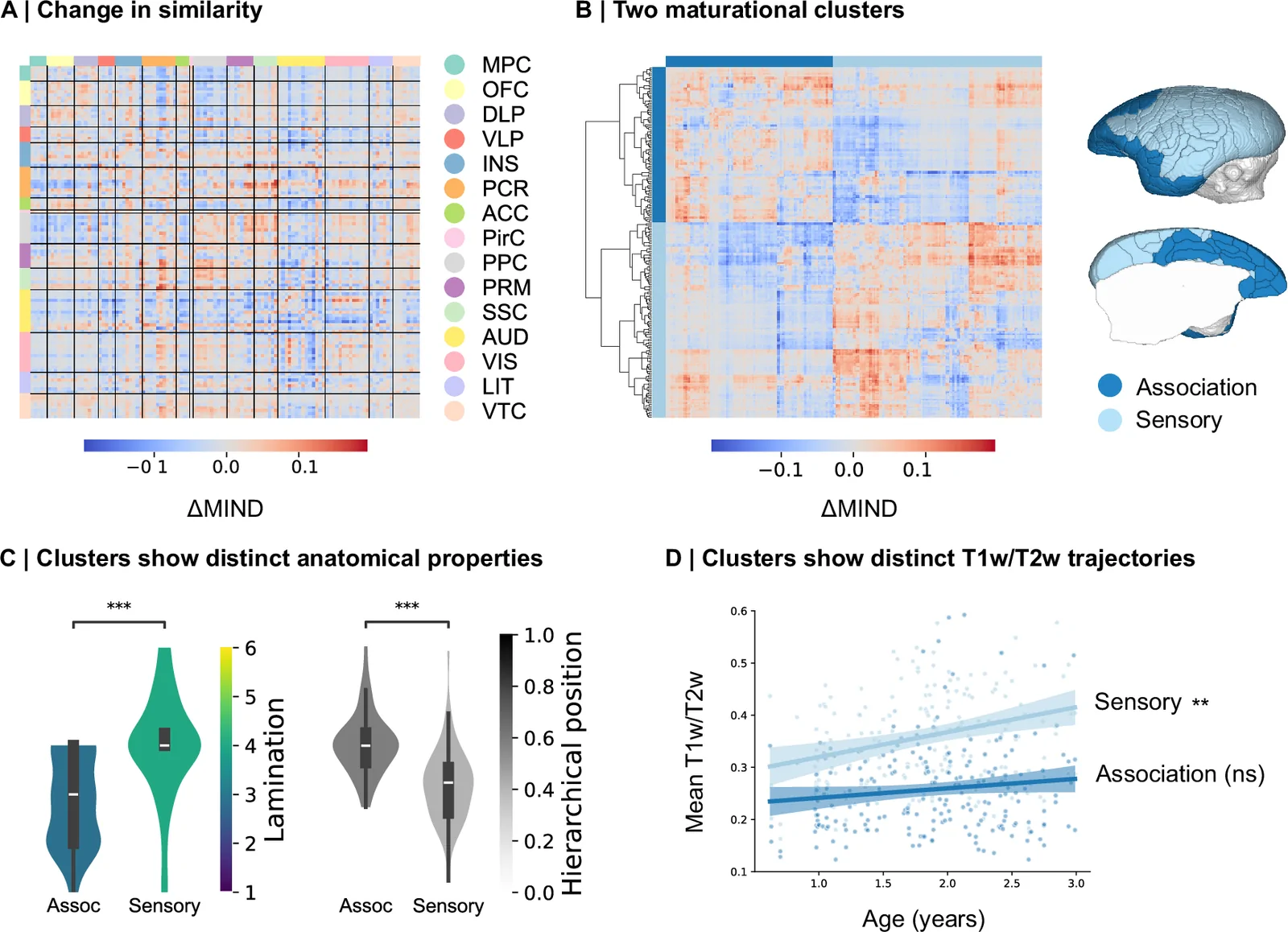
Sensory and association cortex show distinct age-related changes in myelination network properties. **A** For *N* = 211 animals, annual change in T1w/T2w similarity was computed between all pairs of cortical areas (*N* = 230 cortical areas). Matrix shows the left hemisphere, grouped into 15 cortical zones. **B** Hierarchical clustering revealed two cortical clusters with similar patterns of maturation. Matrix shows both hemispheres. **C** Cortical areas were assigned an integer from 1 (Agranular) to 6 (Koniocortex), reflecting their degree of lamination. The sensory cluster had a significantly higher mean degree of lamination and significantly lower mean hierarchical position than the association cluster. Asterisks represent statistical significance, with *p*-values derived from two-tailed, unpaired t-tests evaluated against 1000 spatially autocorrelated null models. Tests compared left hemisphere regions (*N* = 66 in the sensory cluster, *N* = 49 in the association cluster) for which hierarchical position and cytoarchitectonic class data were present. **D** Maturational clusters were differentiated by their linear mean T1w/T2w trajectories. For *N* = 211 animals, mean T1w/T2w was computed for each cluster (as the mean T1w/T2w value across the *N* = 133 and *N* = 97 bilateral regions assigned to the sensory and association clusters, respectively), and developmental change in cluster mean T1w/T2w each was estimated using multiple linear regressions with age and sex as predictors. Asterisks represent the statistical significance of the β_age_ coefficient after multiple (2) comparisons correction using the Bonferroni method. Shading represents the 95% confidence interval of the regression line.

We performed the same regression analyses at each edge. The β_age_ coefficient of each edge, representing the linear rate of change in similarity between each pair of cortical nodes, is shown in Fig. 6A, grouped into cortical zones. This analysis revealed bidirectional changes in similarity of certain cortical areas, which were not apparent in the degree-level analyses. For example, orbitofrontal cortex (OFC) showed decreases in similarity to posterior parietal (PPC), auditory (AUD), and visual (VIS) cortices, and increases in similarity to dorsolateral prefrontal (DLP) and anterior cingulate (ACC) cortices. Mean age-related changes between pairs of cortical zones are visualised in Supplementary Fig. 15A.

We next used agglomerative hierarchical clustering as a data-driven approach to explore how cortical areas were coordinated in terms of their adolescent maturation. This approach iteratively groups cortical areas into clusters based on how statistically similar they are in terms of their patterns of edge-level changes. The two-cluster solution separated the cortex into one cluster containing mainly primary sensory and motor areas (“sensory”) and one cluster containing mainly frontal and paralimbic association areas (“association”; Fig. 6B and Supplementary Fig. 15B). Consistent with these identities, clusters were anatomically differentiable, with the sensory cluster having significantly higher mean degree of lamination (*t* = 2.46, *p*_variogram_ < 0.001) and significantly lower mean hierarchical position (*t* = −2.98, *p*_variogram_ < 0.001; Fig. 6C). Clusters also showed distinct mean T1w/T2w trajectories, with the sensory cluster becoming significantly more myelinated during adolescence (*β*_age_ = 0.047, *p*_FWER_ = 0.001) and increasingly divergent from the association cluster (Fig. 6D). When repeating this analysis using three or four clusters, the association cluster remained largely intact, suggesting that its constituent regions are strongly maturationally coupled (Supplementary Fig. 15C).

We replicated this analysis of age-related changes in MIND network edges using *MBP* expression data. Here, the rate of developmental change was approximated using the difference between a mature adult (4-year-old) and pre-pubertal (6-month-old) male *MBP* MIND network, divided by the difference in age (Supplementary Fig. 16A). We observed large decreases in *MBP* expression similarity between primary sensory and prefrontal cortical regions (Supplementary Fig. 16B). Clusters identified in the *MBP* MIND developmental matrix broadly overlapped with those identified in the T1w/T2w data and showed the same significant pattern of differences in mean lamination and hierarchical level (Supplementary Fig. 16E). However, cross-modal clustering solutions also reflected modality-specific developmental trends, evidenced by a moderate cophenetic distance correlation (Spearman *r* = 0.22) and differences in overlap of clusters with cortical zones (Supplementary Fig. 16C, D).

## Discussion

We estimated structural similarity networks from myelin-sensitive MRI images in the common marmoset and largely confirmed our prior hypotheses: (1) that MRI-derived cortical similarity networks are closely coupled with commensurate networks derived from microscopic measurements of myelin basic protein gene expression and transcription at the cellular level; and (2) that MRI-derived cortical myeloarchitectonic similarity shows developmental changes which are stable across individuals and are different for regions occupying different levels of the sensory-association hierarchy, as in humans. We discuss these hypotheses in light of the existing literature.

T1w/T2w ratio calculation enhances contrast related to myelin in humans^32^. We show, via co-location with spatially resolved brain images of myelin basic protein mRNA, that this applies to T1w/T2w images obtained from common marmosets at high magnetic field strengths. T1w/T2w and *MBP* expression similarity network edges were highly correlated, especially for edges of primary sensory and motor areas, lowest in the cortical hierarchy. The reduced cross-modal concordance of similarity in higher-order cortical areas may arise due to their prolonged developmental and enhanced adult plasticity^42,47^, which would render their myeloarchitectonic structures more sensitive to individual-specific environmental influences and thus increase inter-individual variability of similarity of these areas.

We found that cortical areas with high myeloarchitectonic similarity also had similar whole genome expression profiles in neurons. We interpret this finding in two ways. First, axonal connectivity is guided by the degree of matching between specific patterns of adhesion molecules on neuronal cell surfaces^56,57^ and axonally connected neurons typically exhibit greater similarity in their whole genome expression profiles^58^. Thus, if we interpret similarity as a proxy of axonal connectivity emerging due to homophily^6,7^, supported by our finding that MIND similarity was moderately but significantly correlated (*r* = 0.34) with tract tracing measures of axonal connectivity, similarity and neuronal gene co-expression should also be correlated. Second, despite oligodendrocytes being the key cell type responsible for myelination in the brain, myelination is also dependent on neuronal factors, including the expression of permissive surface molecules and neuronal activity^59,60^. This interdependence may explain the relationship between local myeloarchitecture and neuronal gene expression patterns.

The coupling between myeloarchitectonic similarity and neuronal gene co-expression was strongest for glutamatergic and PV+ and VIP+ interneurons. Previous work has shown that transcriptomic markers of glutamatergic neurons and interneurons (most strongly PV+ but also VIP+ and SST+) correlate strongly with mean T1w/T2w^34,51^ and other indices of cortical hierarchy^50,52^. These neuronal subtypes form anatomically and functionally interconnected cortical microcircuits^61,62^ that strongly reflect local laminar structure^63^. Cortical areas with more similar myeloarchitecture are therefore likely to contain phenotypically similar cortical microcircuits and similar gene expression profiles in the cell types forming these circuits.

Our second main hypothesis centred around adolescence, the age range marked by peak incidence of many psychiatric disorders^64^, and during which increases in cortical myelin contribute to the closure of critical windows of enhanced sensitivity—and vulnerability—to environmental stimuli^65^. Previous studies have shown that these adolescent increases in mean T1w/T2w vary along the cortical hierarchy, with primary sensory areas showing steepest increases and association areas showing shallowest increases^54,55^. We show that this pattern is conserved in the common marmoset, highlighting its translational utility as a primate neurodevelopmental model.

We further demonstrated that T1w/T2w MIND networks show adolescent changes which are stable across individuals and can therefore predict age. Previous work has shown that MIND networks generated from multiple microscale and macroscale MRI features can predict adolescent age in humans^8^. Our work generalises this finding to a simpler context, using a single clinically ubiquitous MRI feature in a primate model. We found that network features predicted age more accurately than mean T1w/T2w alone, suggesting that relative myeloarchitectonic changes between cortical areas are more developmentally informative than absolute changes in mean myelin. Though the enhanced performance of edge-based prediction is unsurprising due to the large number of edges, it is striking that degree-based predictions, with one value per cortical area, out-perform mean T1w/T2w. This stands in contrast to a prior study using morphometric similarity networks (estimated by pairwise correlations of 7 mean macrostructural features^9^), which reported that network features were less accurately predictive of human adolescent age than mean cortical thickness or volume alone^66^. Though we did not use the same MRI features as Griffiths-King et al.^66^, our results suggest that similarity networks estimated using divergence between voxel-level information, rather than using correlations between mean areal feature vectors averaged across all voxels, are more sensitive to age-related cortical changes than mean feature vectors themselves.

We observed marked decreases in the weighted degree of auditory cortical regions, indicating that these regions became increasingly myeloarchitectonically differentiated from the rest of the cortex during adolescence and early adulthood. Previous research in marmosets has demonstrated that vocal behaviours show protracted development, remaining plastic in adulthood^67^, and that the response properties of auditory cortical neurons to self-vocalisations change following manipulation of the laryngeal apparatus in adults^68^. This sustained experience-dependent plasticity may contribute to the increasing structural differentiation we observed, as individual vocal and social experiences shape local microstructural properties over time. We also observed myeloarchitectonic differentiation in regions functionally connected to auditory cortex within a broader auditory processing network, particularly the anterior cingulate cortex^69,70^ (Supplementary Fig. 14B), suggesting that experience-dependent differentiation may extend beyond primary auditory fields to distributed circuitry supporting vocal communication.

Edge-level similarity network changes were organised along an axis from primary sensory to transmodal association areas^40–42^. Cortical areas occupying similar positions along this axis showed similar patterns of edge level maturation, with particularly strong maturational coupling observed within a rostromedial transmodal association cluster that overlapped a putative human default mode network homologue identified by previous analysis of tract tracing data^71^. In human cortex, myelination networks estimated as the pairwise correlations between regional MT (Magnetisation Transfer) depth profiles showed a convergent profile of adolescent reorganisation, with developmental changes organised along a latent axis mirroring the sensory-association gradient^43^. MT profiles of primary sensory (idiotypic) cortex became increasingly divergent from paralimbic cortex, and intermediate areas became either more idiotypic-like or paralimbic-like, indicating a progressive polarisation of the sensory-association axis. Likewise, we observed that areas located at opposite ends of the sensory-association gradient tended to diverge maturationally in their myeloarchitecture, with large decreases in similarity between primary sensory areas and prefrontal, temporal, and paralimbic association areas. Parallel evidence from rat cortex indicates that prefrontal areas become myeloarchitectonically differentiated from primary sensory areas between late adolescence and mid-adulthood in MIND similarity networks estimated from myelin-sensitive MT images^11^. Taken together, these findings suggest that the sensory-association axis represents a conserved gradient of myelinated cortical network reorganisation during adolescence.

Our study has limitations. First, although T1w/T2w ratio is undoubtedly sensitive to myelination, it is not a direct measure of myelin. Hence, though we observed strong concordance between mean T1w/T2w and mean *MBP* expression across the cortical surface, there were differences in the signal distribution shapes (standard deviation and skewness) between modalities. These differences may arise because: (i) T1w/T2w images are sensitive to non-myelin factors such as iron and water content^32,72^; and (ii) noise and partial voluming effects introduced during MRI acquisition may influence regional T1w/T2w distribution shapes, particularly at the white matter surface^73^. These non-myelin factors, through influencing regional T1w/T2w distribution shapes and therefore the inter-areal similarity of regional T1w/T2w distributions, constitute a source of non-myeloarchitectonic variability in T1w/T2w similarity networks, which could explain why we observed only nominally significant correlations between T1w/T2w MIND and gene co-expression. The absence of these non-myelin factors in *MBP* images means that they more faithfully represent cortical myelination patterns and provide a ceiling for correlations between myeloarchitectonic similarity and gene co-expression. Furthermore, we did not observe significant correlations between myeloarchitectonic similarity and oligodendrocyte gene co-expression, which we expected given that oligodendrocytes are the key cell type responsible for myelination. We suspect that this is due to insufficient statistical power: expanding the number of cortical areas for which single cell RNA sequencing data are available (beyond the 12 areas openly provided by Krienen et al.^39^, which allow estimation of 66 pair-wise inter-areal similarities or edges) would allow for a finer-grained and more comprehensive comparison between cortical networks of structural similarity and gene co-expression.

Second, it would in principle be interesting to analyse the relationships between MIND MRI phenotypes and excitatory neuronal subtypes. Previous work has shown that excitatory subtypes differ in their association with mean cortical myelin across the cortex^34,51^. Moreover, activity-dependent myelination is influenced by factors such as growth factor signalling and neuronal diameter^74^, both of which vary across glutamatergic subtypes. However, for reasons detailed in Supplementary Methods, here we restricted our focus to inhibitory inter-neuronal subtypes.

Third, we used linear modelling approaches as an interpretable way to model age-related changes and to facilitate cross-modal comparisons with *MBP* expression data, for which we only had access to two timepoints, though we expect that cortical myelination similarity would show non-linear trajectories. In humans, developmental increases in cortical mean T1w/T2w follow non-linear trajectories, with primary sensory areas reaching peak myelination first, and association areas last^54,75^. A comparable chronology has been observed for grey matter volume decreases in marmoset cortex^17^, thought to reflect progressive increases in cortical myelination^76^. Furthermore, cortical myelin undergoes dynamic changes across the lifespan. Indeed, myelination begins *in utero* for sensory and motor areas^36^, while ageing involves cortical de-myelination. The regional sequencing of age-related myelin decreases is thought to be the chronological reverse of the sequence of adolescent myelin increases, reflecting a “first in last out” hypothesis of senescence^75^. The field would benefit from an exploration of non-linear lifespan trajectories of myelination network features, for example, using normative modelling approaches^77,78^, and from an investigation of how these trajectories relate to brain function, cognition, and psychiatric outcomes.

## Methods

### T1w/T2w ratio data and preprocessing

We used T1-weighted (T1w) and T2-weighted (T2w) images acquired at 9.4 T from the Brain/MINDS project, currently the world’s largest open-access marmoset MRI data resource^28^. The dataset contains cross-sectional MRI images from *N* = 446 marmosets, spanning 0.62–12.75 years (*N* female = 238). T1w images were collected using a magnetisation-prepared rapid gradient echo (MP-RAGE) with repetition time (TR) = 6000 ms, echo time (TE) = 2 ms, flip angle = 12°, number of averages (NA) = 1, inversion time = 1600 ms, and scan time = 20 min. T2w images were collected using rapid acquisition with relaxation enhancement (RARE) with TR = 4000 ms, TE = 22 ms, RARE factor = 4, flip angle = 90°, NA  =  1, and scan time = 7 min, 24 s. Reconstructed T1w and T2w images had identical matrix dimensions (178 × 178 × 96), voxel sizes (0.27 mm isotropic), orientations (RAS), and voxel-to-RAS affine transformation matrices, indicating a one-to-one correspondence of voxel coordinates across modalities. For full details on image acquisition, see Hata et al.^28^.

Volumetric images were parcellated according to the 2019 Brain/MINDS Atlas^79^ (BMA 2019), which is based on the histological atlas defined by Paxinos et al.^45^. Cortical areas were defined with separate left and right counterparts. For a full list of cortical areas used in our analyses, see Supplementary Data 1. The full preprocessing pipeline is visualised in Supplementary Fig. 1. Briefly, T2w images were skull stripped using BrainSuite18a^80^ to improve the quality of subsequent image registration, for which we used advanced normalisation tools^81^ (ANTs). An ex-vivo T2w template^79^ was warped to each T2w image, after which the warp was applied to the BMA 2019 atlas to generate a label image for each subject in native MRI space. Animals were analysed in native space to preserve the original resolution and distributional characteristics of voxel-level MRI features. Voxel-wise division of the T1w image by the T2w image generated a T1w/T2w ratio image, which increased myelin-related contrast and reduced intensity inhomogeneity bias^32^. T1w and T2w images were not co-registered prior to calculation of T1w/T2w images because: (i) reconstructed T1w and T2w image volumes were provided on identical native-space sampling grids; and (ii) marmosets were anaesthetised and head-fixed before scanning, minimising head motion between T1w and T2w image acquisitions^28^.

### Similarity network estimation using Morphometric Inverse Divergence

For each parcellated T1w/T2w image, a distribution of voxel values was extracted from each cortical area. Similarity between each pair of voxel value distributions was estimated using morphometric inverse divergence (MIND^8^), yielding a univariate (single feature) similarity network. MIND is the inverse of the Kullback-Leibler (KL) divergence, a statistical measure of divergence or dissimilarity between two univariate or multivariate distributions^44^. For two sets of voxels (or vertices from a surface reconstruction) in cortical areas *a* and *b* following distributions *P*_*a*_ and *P*_*b*_, the KL divergence between them is calculated as:

1 $${D}_{{KL}}\left({P}_{a}\parallel {P}_{b}\right)=\int {p}_{a}\left(x\right) log \left(\frac{{p}_{a}\left(x\right)}{{p}_{b}\left(x\right)}\right){dx\geq0}$$

Where *p*_*a*_*(x)* and *p*_*b*_*(x)* are the probability densities at specific values of *x*. KL is then symmetrised, normalised, and inverted, converting it to a measure of similarity:

2 $${MIND}\left(a,b\right)=\frac{1}{1+{D}_{{KL}}\left({P}_{a}\parallel {P}_{b}\right)+{D}_{{KL}}\left({P}_{b}\parallel {P}_{a}\right)}$$

Estimation of MIND for each possible pair of areas in the cortical parcellation results in a similarity matrix, where each element (edge) represents the similarity in voxel distributions between a pair of cortical areas, ranging from >0 to 1. Low values indicate a highly dissimilar, divergent or differentiated pair of cortical areas, and high values indicate a highly similar, convergent, or near-identical pair of cortical areas.

Two elementary features of each MIND network were the focus of further analysis: nodes and edges. Edge weights represent the pair-wise similarity between cortical nodes, and the weighted degree of each node is simply the average of the weights of all edges linking that node to the rest of the cortical network.

### Estimation of KL divergence

Non-parametric estimation of the KL divergence between two (voxel) distributions requires a systematic comparison of probability densities, which can be done either globally or locally. Global methods involve comparing coarse partitions of the distributions, e.g., corresponding histogram bins, while local methods involve comparing densities in the local neighbourhoods surrounding individual distribution values, e.g., voxel values (Supplementary Methods). Though both global and local KL estimation methods have previously been used to estimate structural similarity networks from MRI data^8,82^, they have not been directly compared. We addressed this gap in the literature by systematically comparing a global and local KL estimator. We used a global estimator of KL, which binned the data into histograms^83^ using in-house code. Secondly, we used the local estimator implemented in the current MIND algorithm^8^ based on a k-nearest neighbours (k-NN) approximation (Supplementary Methods).

To facilitate comparisons between similarity networks generated using different KL estimators, and variable parameters of each of those estimators, we adopted a previously used set of anatomical benchmarks^8,11^. Anatomical benchmarking assumes that the “best performing” or “most optimal” MRI similarity networks are ones that correspond most strongly with the underlying anatomy. In our case, benchmarks were based on cytoarchitecture, interhemispheric symmetry, and axonal connectivity (Supplementary Methods). We additionally compared MRI similarity networks resulting from these KL estimation algorithms using an age prediction task. Here, the networks producing most accurate age predictions, assessed via their partial correlation and mean absolute error relative to actual ages, were deemed “most optimal”.

### Marmoset anatomical data

#### Cytoarchitecture

We used a cytoarchitectonic class assignment map^46^, in which cortical regions were assigned to one of six classes based on expert classification of histological sections (Supplementary Fig. 6B). In order of increasing lamination, the classes are: agranular (Ag), dysgranular (Dys), eulaminate I (Eu I), eulaminate II (Eu II), eulaminate (Eu III), and koniocortex (Kon).

#### Axonal connectivity

Tract tracing data were obtained from Majka et al.^22^. Data from 143 retrograde tracer injections in 52 adult marmosets (*N* female = 21, aged 1.4–4.6 years) were used to generate a single {55 × 116} tract tracing matrix. Retrograde tracer injections were placed in 55 target regions, labelling axonal tracts originating from cell bodies in 116 source regions. Quantitative analysis of histological sections was used to estimate the fraction of labelled neurons (FLN) originating in each source brain region for each of the 55 target regions receiving a tracer injection. The logarithm of the estimated fraction of labelled neurons in each region, log_10_(FLN), averaged across animals, was used as the key metric of axonal connectivity. Log_10_(FLN) ranges from 0 (100% of tracts originate in source) to -∞ (0% of tracts originate in source). Because this approach fails to capture axonal pathways terminating in the 61 regions not injected with tracer, we used the edge-complete matrix of {55 × 55} regions that had received at least one injection. For comparability, MIND similarity matrices were restricted to the same 55 regions in all analyses involving tract tracing data. Edge-level analyses used the 1485 edges in the upper triangle of the tract tracing matrix.

#### Position in the cortical hierarchy

A cortical area can be assigned to a position in the cortical processing hierarchy based on its laminar pattern of incoming and outgoing axonal connections^84^. This is based on the observation that feedforward connections, carrying information up the cortical hierarchy from sensory to association regions, arise mainly from supragranular layers and terminate in layer IV^85^. The proportion of tracts a cortical area receives which originate in supragranular layers has therefore been used to estimate cortical maps of hierarchical position in the marmoset^25^, with regions receiving a high proportion of feedforward information located at the top of the hierarchy and regions receiving a low proportion of feedforward information located at the bottom. We used the hierarchical position map from Theodoni et al.^25^, which assigns to 110 of 115 cortical areas a value ranging from 0 (bottom of the hierarchy: primary visual cortex) to 1 (top of the hierarchy: agranular insula). This map is visualised in Supplementary Fig. 6C.

### Marmoset transcriptional data and pre-processing

#### In-situ hybridisation data

We downloaded spatially resolved images of myelin basic protein (*MBP*) messenger RNA (mRNA) from the Marmoset Gene Atlas^37,38^. Images were collected using in-situ hybridisation (ISH), a histochemical technique allowing spatial localisation of specific nucleic acids within a tissue sample^86^. We downloaded in-situ hybridisation images of *MBP* mRNA from a 6-month-old (pre-pubertal) male and a 4-year-old (mature adult) male marmoset. For both animals, *MBP* mRNA was measured in 90 coronal brain slices, spaced 196 μm apart^37,38^. Though the posterior pole of occipital cortex and the anterior pole of frontal cortex were not completely covered, each brain region in the Paxinos et al.^45^ parcellation was present in a sufficient number of slices to constitute a representative sample of brain tissue for analysis at both ages.

To facilitate a quantitative comparison of T1w/T2w and *MBP* mRNA images, in-situ hybridisation slices were stacked to form a volume, which was then linearly and nonlinearly deformed to the BMA 2019 T2w template^79^. To do this, we implemented a preprocessing pipeline, fully outlined in Supplementary Fig. 7, that used a combination of in-house code and openly available code^87^. This pipeline allowed us to estimate the distribution of *MBP* mRNA expression in each cortical area, with comparable spatial resolution to the T1w/T2w images (isotropic voxel resolution of *MBP* mRNA images = 0.10 mm; T1w/T2w images = 0.27 mm). The resulting cortical maps were subsequently used to estimate MIND networks representing inter-areal similarity of *MBP* expression on the same spatial scale and using the same areal parcellation as the MIND networks derived from T1w/T2w images. Following this procedure, we had tissue-level maps of *MBP* mRNA, which could be directly associated with MRI-derived T1w/T2w maps, and networks of *MBP* expression similarity, which could be topologically associated with networks of T1w/T2w similarity.

#### Single-cell, whole genome mRNA data

We used open-access single nucleus RNA sequencing data from Krienen et al.^39^. This dataset contains whole genome transcripts from multiple cortical and subcortical regions comprising six cell types: glutamatergic excitatory neurons (glut), glutamic acid decarboxylase-expressing inhibitory interneurons (GAD), oligodendrocytes (oligo), astrocytes (astro), microglia and macrophages (immune), and endothelial cells (endo). Further details on data collection, quality control, and cell type classification are provided by Krienen et al.^39^.

Cortical dissections in Krienen et al.^39^ were guided by the Paxinos et al.^45^ atlas. Some dissections were restricted to individual Paxinos regions, while others were inclusive of several neighbouring regions, constituting a larger amalgamated area. This resulted in a partial parcellation of cortex into 12 relatively coarse-grained areas (Fig. 3A and Supplementary Table 2). We used this scheme to coarse-grain the more finely parcellated T1w/T2w and *MBP* mRNA images so that they were anatomically aligned with the cortical regions for which single-cell sequencing data were available. Further details on the coarse parcellation can be found in Supplementary Table 2, and the sex and ages of the sampled animals are summarised in the figure legend.

We used these data to generate cell type-specific correlated gene expression (CGE) matrices. Cell-by-gene matrices for each cell type were downloaded from the CELLxGENE data repository (RRID:SCR_021059). Library size normalisation, i.e. normalising gene expression across cell types to correct for differences in sequencing depth, had already been applied to the data^39^. We retained the top 10% most variable genes for CGE network calculation using Scanpy’s highly_variable_genes function^88^. Our reasoning was that genes with low variability in expression across the cortex are unlikely to drive systemic variation in cortical myeloarchitecture. Indeed, previous work showed that transcriptomic variation in a subset of 200 genes that are most highly variable across cortex is closely coupled to variation in mean T1w/T2w of human visual cortex^89^. Within each cell type, we averaged log normalised expression across cells with the same regional annotation to form a region-by-gene expression matrix. Pearson correlations between each pair of regional gene expression vectors were used to estimate the final CGE matrix for each cell type (Fig. 3A). The same process was applied to interneuron subtypes to estimate interneuron subtype-specific correlated gene expression matrices.

### Statistical analysis

#### Correlation

Unless stated otherwise, correlations were estimated using Spearman’s *rho*. *P*-values were obtained from two-tailed tests. Shading around regression lines indicates the 95% confidence interval estimated using 1000 bootstraps.

#### Multiple linear regression

To estimate linear changes in nodal properties (mean T1w/T2w and degree) and edge weights during development, we used multiple linear regressions with age as the independent variable. Correction for multiple comparisons used the Benjamini–Hochberg FDR (false discovery rate) adjustment. We included sex as a covariate in all models, as studies have demonstrated sex differences in regional cortical mean T1w/T2w in humans^90,91^. However, the main effect of sex was not significant in any model after correction for multiple comparisons. Furthermore, some studies indicate that humans display sex-specific adolescent trajectories of cortical mean T1w/T2w^92^. Therefore, we assessed the significance of an age-by-sex interaction term in our models. However, this interaction term was ultimately dropped from all models as it did not reach significance in any model after multiple comparisons correction.

#### Linear regression sensitivity analyses

Some studies undertake additional steps to normalise T1w/T2w scans to improve inter-subject comparability. These studies normalise by features such as median white matter intensity^93^ and median vitreous humour intensity^94^. We therefore conducted sensitivity analyses showing that the inclusion of these features as covariates to our mean T1w/T2w models did not substantially alter regional *β*_age_ estimates (Supplementary Fig. 12B).

Previous research demonstrated a positive correlation between estimated total intracranial volume (eTIV) and average across all MIND network edges (global MIND^8^), which may reflect allometric scaling relationships between brain size and network connectivity^95^. Though we did not observe a significant correlation between eTIV and global MIND (*r* = −0.12, *p* = 0.079), we conducted sensitivity analyses demonstrating that addition of eTIV as a covariate did not substantially alter estimates of regional weighted degree *β*_age_ coefficients (Supplementary Fig. 14C).

#### Spatial null models

In spatially embedded systems, such as the brain, regions that are physically closer to each other tend to be more similar across a wide range of features, including axonal connectivity profiles, cytoarchitectonic structure, and gene expression^58,96^. This spatial dependence, or autocorrelation, between neighbouring brain areas violates the assumption of independence in standard statistical inference procedures and can lead to inflated type I error rates if not properly addressed^96^. We therefore used spatial autocorrelation-preserving null models, generated using BrainSMASH^97^, for statistical inference on parcellated cortical maps. BrainSMASH implements an algorithm that estimates for a particular brain map (for instance, mean T1w/T2w) the variance in regional values as a function of the Euclidean distance between regions (the variogram) and generates surrogate brain maps with matched variograms^98^ that can be used to construct an appropriate null distribution of test statistics. BrainSMASH permuted maps were computed from left hemispheric data only. All tests used 1000 permutations.

#### Network null models

BrainSMASH-generated surrogate T1w/T2w or *MBP* expression maps were used to permute the rows and columns of respective MIND similarity networks, generating null networks with preserved autocorrelation structure. This procedure was applied to the full left hemisphere network, then coarse-grained appropriately when axonal connectivity or correlated gene expression matrices were concerned. To obtain a *p*-value for the correlation between T1w/T2w MIND and Euclidean distance (Fig. 1C), we used network null models generated by applying random permutations to rows and columns of the T1w/T2w MIND matrix. All statistical tests used 1000 permutations.

#### Hierarchical clustering

To explore how cortical areas were coordinated in terms of their adolescent maturation, we used agglomerative hierarchical clustering with Euclidean distance and Ward linkage. To quantitatively compare clustering solutions between modalities (T1w/T2w and *MBP* expression), we calculated the correlation between their cophenetic distance matrices^99^. The cophenetic distance between two nodes or cortical areas is defined as the dendrogram height at which they first join the same cluster. The matrix of cophenetic distances between each pair of cortical areas thus summarises the overall structure of the hierarchical clustering solution. A high correlation between cophenetic distance matrices indicates that the two clustering solutions impose a similar hierarchical organisation on the data, reflecting concordance in the maturational coordination of cortical areas across modalities.

### Age prediction

To assess the extent to which MIND networks capture developmental changes in myelination, we trained machine learning models to predict age from each subject’s T1w/T2w scan. The input features (predictors) used were regional mean T1w/T2w, regional weighted degree, or all edge weights. Although the large majority of intracortical myelination occurs before the end of adolescence, increases have been shown to continue into adulthood in humans^75^. Consistent with this, mean T1w/T2w in some marmoset cortical areas continued increasing beyond the onset of adulthood (18–20 months^16,17^), plateauing by 36 months (Supplementary Fig. 11). To capture this protracted period of myelination, we trained models using animals aged between 7 months and 36 months, yielding *N* = 211 subjects (*N* female = 91).

For each predictor, we trained and tested models on 50 random splits of the data (20% of subjects retained in each test fold), stratified by sex. Within each outer training fold, 5-fold cross validation, again stratified by sex, was used to select between several models. For regional predictors (mean T1w/T2w and weighted degree), we used: a support vector regression with a radial basis function kernel and C regularisation values of [0.1, 1, 10, or 100]; and a Gaussian process regression (GPR) with a summed linear and white noise kernel and alpha value of 1 × 10^−10^. We did not use other values of alpha in our final GPR models to reduce computation time and because preliminary tests demonstrated that modulating alpha to [10^-5^, 10^−3^, or 10^−2^] did not change model predictions. The model with the minimum mean absolute error (MAE) across 5 internal validation folds was trained on the entire training set for that outer fold. At the edge level, due to the much larger feature space (26,335 edges), we used only the GPR model^8^.

For each outer test fold, models were evaluated using the mean absolute error (MAE) and the partial correlation between actual and predicted age values. We used partial correlations to control for the effects of sex and eTIV. Regressing variables out of the feature space can lead to data leakage as estimating effects from the entire dataset allows information from the test set to indirectly influence the training process, inflating model performance^100^. We therefore opted to use partial correlations as a post-hoc adjustment^8^.

### Statistics and reproducibility

#### Sample size

No power analyses were conducted prior to data analysis. The full sample size available to us was *N* = 446 (*N* female = 238). After manual and automated quality control, the sample size available was *N* = 421 (*N* female = 226). For non-linear analyses, we additionally excluded animals in age ranges with a sampling density below 5%, leaving *N* = 416 animals (*N* female = 223), aged 0.62–10.67 years. For developmental analyses, we retained animals below 36 months of age, leaving *N* = 211 (*N* female = 91). This sample size was sufficiently large to yield stable age predictions when using 5-fold cross-validation: the MAE across 50 folds was approximately 3.5 months when using network edges as predictors (Fig. 5B).

#### Quality control

To exclude outliers in MRI-derived phenotypes, we used a combination of manual and automated quality control procedures. First, we visually inspected all T1w/T2w images and manually excluded scans with large artefacts or warping resulting from poor image registration (*N* = 6). We also automatically excluded scans falling in the top or bottom 1% of mean T1w/T2w or MIND weighted degree across all regions (*N* = 20). Furthermore, because automated spline smoothness selection is unstable in age ranges with sparse data^101^, we excluded animals falling in age ranges with a sampling density below 5% (Supplementary Fig. 11).

#### Reproducibility

Reproducibility of MRI-based findings was assessed using a transcriptomic proxy for myelination collected in an independent sample of spatially resolved images of myelin basic protein (*MBP*) mRNA expression, measured using in situ hybridisation, in two male marmosets (aged 6 months and 4 years). Key findings were replicated across modalities, namely, regional patterns of mean cortical myelination in adults, inter-areal myelination similarity network organisation, concordance with cell type-specific gene co-expression, and the pattern of adolescent myeloarchitectonic change in regional myelination and similarity network metrics.

#### Ethics oversight

All data analysed in this study were previously published and collected in accordance with relevant ethical standards.

### Reporting summary

Further information on research design is available in the Nature Portfolio Reporting Summary linked to this article.

## Supplementary information

Supplementary Information

Description of Additional Supplementary Materials

Supplementary Data 1

Reporting Summary

Transparent Peer Review file

## Data Availability

All raw data accessed in this study are openly available. Retrograde tract tracing data^22^ are available from http://marmosetbrain.org. Hierarchical location assignments for cortical areas^25^ are available from https://doi.org/10.1093/cercor/bhab191. Cytoarchitectonic class assignments^46^ are available from https://www.marmosetbrain.org/whole_brain_cb_maps. MRI data^28^ are available from the Brain/MINDs 2.0 data portal (https://dataportal.brainminds.jp/marmoset-mri-na216). In-situ hybridisation data^37,38^ are available from the Brain/MINDs Marmoset Gene Atlas data portal (https://gene-atlas.brainminds.jp/). Processed single nucleus RNA-sequencing data^39^ are available from the CELLxGENE data repository (RRID:SCR_021059; https://cellxgene.cziscience.com/collections/0fd39ad7-5d2d-41c2-bda0-c55bde614bdb). Marker gene sets used to assign biological identities to interneuron clusters are available from https://doi.org/10.1038/s41586-021-03465-8. Intermediate data files generated in this analysis are openly available at https://github.com/edhutch1/marm_MIND and https://doi.org/10.5281/zenodo.19758838. All other data are available from the corresponding author on reasonable request.

## Code availability

Preprocessing of MRI data used Brainsuite18a^80^ and Advanced Normalisation Tools^81^ (ANTs). Preprocessing of in-situ hybridisation data used a modified version of previously published Matlab code^87^ (https://github.com/TrangeTung/marmoset_gradient) and ANTs. Computation of MIND networks used previously published Python code^8^ (https://github.com/isebenius/MIND). Downstream analyses were conducted in Python 3.11 using publicly available packages. Code and data required to reproduce all the analyses and results reported in this paper are openly available at https://github.com/edhutch1/marm_MIND and https://doi.org/10.5281/zenodo.19758838.
