# Author Correction: The cellular correlates and adolescent reorganisation of cortical myelination networks in the common marmoset

**DOI:** 10.1038/s42003-026-11090-2

**Published:** 2026-10-05

**Authors:** E. D. Hutchings, S. J. Sawiak, R. L. Smith, R. A. I. Bethlehem, A. C. Roberts, E. T. Bullmore

**Affiliations:** 1 Department of Psychiatry, University of Cambridge, Cambridge, UK; 2 Department of Physiology, Development and Neuroscience, University of Cambridge, Cambridge, UK; 3 Human Genetics Branch, National Institute of Mental Health, Bethesda, MD USA; 4 Department of Psychology, University of Cambridge, Cambridge, UK; 5 School of Academic Psychiatry, Institute of Psychiatry, Psychology & Neuroscience, King’s College London, London, UK

**Keywords:** Neuroscience, Developmental biology, Development of the nervous system

Correction to: *Communications Biology* https://doi.org/10.1038/s42003-026-10276-y, published online 19 May 2026

In the version of this article initially published, there were errors in the Fig. 2c labels, where the text now reading “*r* = 0.73” and “*P*_variogram_ = 0.001” originally read “*r* = 071” and “*P*_variogram_ = 0.002,” and in the Fig. 2e labels, where “*r* = –038” and “*P*_variogram_ = 0.206” appeared originally as “*r* = –036” and “*P*_variogram_ = 0.217.” In the second paragraph of the “T1w/T2w similarity networks align with molecular and cellular transcriptomic markers of myeloarchitecture” section, the text now reading “*p*_variogram_ = 0.206” replaces the original “*p*_variogram_ = 0.208.” The changes have been made in the HTML and PDF versions of the article.

