# Supplementary material for: The cellular correlates and adolescent reorganisation of cortical myelination networks in the common marmoset
Source: Commun Biol. 2026 May 19;9:1066. doi: 10.1038/s42003-026-10276-y (PMC13454510; doi:10.1038/s42003-026-10276-y)
Supplement: Supplementary file 1 [file 42003_2026_10276_MOESM1_ESM.pdf]

Supplementary Information

The cellular correlates and adolescent reorganisation of cortical myelination networks in the common marmoset

E D Hutchings[1, *], S J Sawiak[2], R L Smith[1, 3], R A I Bethlehem[4], A C Roberts[2], E T Bullmore[1, 5]

[1]Department of Psychiatry, University of Cambridge, Cambridge, UK
[2]Department of Physiology, Development and Neuroscience, University of Cambridge, Downing Street, Cambridge, UK
[3]Human Genetics Branch, National Institute of Mental Health, Bethesda, MD, USA
[4]Department of Psychology, University of Cambridge, Cambridge, UK
[5]School of Academic Psychiatry, Institute of Psychiatry, Psychology & Neuroscience, King's College London, London, UK

* Correspondence to E D Hutchings. Email: eh636@cam.ac.uk

Contents

Supplementary Methods **3**
Local and global estimators of KL divergence 3
Edge-level effects of tuning parameters in KL divergence estimators 4
Anatomical benchmarks 4
Selecting optimal Histogram parameters using anatomical benchmarks 5
Cell type-specific correlated gene expression network analysis 6
Non-linear analyses 7
Rationale for linear modelling of developmental changes 8

Supplementary Data **9**
1. Regions used in whole-cortex analysis 9

Supplementary Tables **10**
1. Tuneable parameters in KL estimators 10
2. Coarse-grained parcellation used in single cell transcriptomic analyses 11
3. Features best fit by linear and non-linear models 12

Supplementary Figures **13**
1. Preprocessing of T1w/T2w ratio images 13
2. Edge-level effects of tuning parameters in KL divergence estimators 14
3. Selecting optimal Histogram algorithm parameters 15
4. Local algorithm out-performs global on anatomical benchmarks and age prediction 16
5. Seed-based MIND maps for the mean adult T1w/T2w MIND network 17
6. Anatomical maps 18
7. Preprocessing of in-situ hybridisation images 19
8. Comparing T1w/T2w and *MBP* expression distribution shapes 20
9. Cross-modal concordance of MIND edges and cross-modal similarity of signal intensity distributions 21
10. Assignment of interneuron clusters to subtypes 22
11. Feature values commonly plateau between 2 and 2.5 years 23
12. Age-related changes in T1w/T2w 24
13. Adult maturational plateau is robust to bin size 25
14. Auditory regions become microstructurally differentiated from the rest of the cortex 26
15. Maturational clustering sensitivity analyses 27
16. Maturational changes in *MBP* expression similarity are concordant with changes in T1w/T2w ratio similarity 28

Supplementary References **29**

Supplementary Methods

Local and global estimators of KL divergence

Non-parametric estimation of the KL divergence between two (voxel) distributions requires a systematic comparison of their probability densities. In the univariate (single feature) case, this can be performed using histograms, kernel density estimation (KDE) or nearest neighbour methods[1]. Histograms and KDE estimate a probability density function over all points in a distribution. We therefore refer to them as “global” estimators. For a pair of cortical areas, a and b, following distributions P_a and P_b, the probability densities at equivalent values of x are successively substituted into equation (1) to estimate KL:

$$D_{KL}(P_a \parallel P_b) = \int p_a(x) \, log\left(\frac{p_a(x)}{p_b(x)}\right) dx \qquad (1)$$

Where $p_a(x)$ and $p_b(x)$ are the probability densities at equivalent values of x, i.e. at equivalent bins if using histograms[2]. Previous studies have used KDE to estimate KL divergence-based similarity networks from voxel-wise grey matter volume[3–5]. These studies have shown that univariate similarity networks exhibit high test-retest reliability[4,5] and are robust to variation of smoothing parameters and parcellation schemes[5]. These networks also show bidirectional changes in grey matter similarity following sleep deprivation[3] and decreases in global efficiency and clustering of networks during healthy ageing[4].

Conversely, nearest neighbour methods involve comparisons of the local density around individual (voxel) values, rather than between relatively coarse partitions of the data[1]. We therefore refer to them as “local” methods. MIND has previously been estimated with a k-Nearest Neighbours (k-NN)-based KL estimator developed by Perez-Cruz[6]. For two sets of voxels, V_a and V_b, following distributions P_a and P_b, KL is estimated as:

$$D_{KL}(P_a \parallel P_b) = -\frac{d}{n}\sum_{i=1}^{n} log\left(\frac{r_k(x_i)}{s_k(x_i)}\right) + log\left(\frac{m}{n-1}\right) \qquad (2)$$

Where d is the number of structural features measured at each voxel, n and m are the number of values (voxels) in V_a and V_b respectively, and $r_k(x_i)$ and $s_k(x_i)$ are the differences between the voxel value x_i to the most similar voxel value in V_a (its own distribution) and V_b (the other distribution), respectively.

Edge-level effects of tuning parameters in KL divergence estimators

Histogram and k-NN estimators of KL divergence include several tuneable parameters. For the histogram algorithm, these are: the number of bins used to partition the data; epsilon, a small constant added to $p_b(x)$ in equation (2) to prevent division-by-zero errors; and the percentiles of data retained when calculating histogram bins. For the k-NN algorithm, these are: *k*, the kth nearest neighbour used to estimate local density; and the percentiles of data retained. Full parameter descriptions and the default values used in our KL estimators are provided in **Supplementary Table 1**.

We reasoned that the choice of parameters may impact the estimated value of KL at a single edge and thus may impact the overall MIND network structure. We therefore undertook a series of simulations to assess which parameters had the largest impact on KL estimation across one edge and all network edges. At the single edge level, we estimated KL between two empirical T1w/T2w voxel distributions extracted from a single subject (**Supplementary Figure 2A**) using default parameter values. We then independently varied each KL estimation parameter in the k-NN and Histogram algorithm and calculated the percentage change in estimated KL from default (**Supplementary Figure 2B**). This revealed that the percentiles parameter for both algorithms and the number of bins used in the histogram algorithm had the largest effects on the value of KL. We then performed a similar analysis at the whole network level (i.e. over all edges), by first generating a full MIND network using default KL parameters (**Supplementary Figure 2C**) and assessing how modulating parameters altered the edge-wise correlations with the default network. This revealed, again, that the same parameters led to largest changes in network edge structure (**Supplementary Figure 2D**). Our simulations indicate that the percentiles parameter for both algorithms and the number of bins used in the histogram algorithm should be carefully selected prior to downstream analysis as they generate large changes in MIND network structure.

Anatomical benchmarks

To facilitate comparisons between similarity networks generated using different KL estimators, and variable parameters of each of those estimators, we adopted a previously used set of anatomical benchmarks[7,8]. Anatomical benchmarking assumes that the “best-performing” or “most optimal” MRI similarity networks are ones that correspond most strongly with the underlying anatomy. In our case, benchmarks were based on cytoarchitecture, interhemispheric symmetry, and axonal connectivity.

Cytoarchitecture

One explanation for macroscale MRI similarity is a result of similarity at the microscale[9]. Therefore, we expect that regions of the same cytoarchitectonic class, due to having similar laminar organisation on microscopy, should tend to be similar in terms of their T1w/T2w voxel distributions. In this case, networks which have a higher proportion of intra-class edges over a range of densities, i.e. those with a higher area under the curve (AUC), are assigning higher macroscale T1w/T2w similarity to pairs of regions with higher microscale myeloarchitectonic similarity, which is favourable.

We used an openly available cytoarchitectonic class assignment map[10], described in **Methods** and illustrated in **Supplementary Figure 6B**.

Interhemispheric symmetry

Though notable cortical asymmetry exists, homotopic regions are highly structurally similar due to the shared genetic effects of antero-posterior morphogen gradients during early patterning of the telencephalon[11]. Our second benchmark was therefore to assess the proportion of all (115) homotopic edges that were present across a range of network densities[7]. Networks with a higher AUC are more optimally capturing high similarity between homotopic cortical areas.

Axonal connectivity

Due to the principle of homophily, regions with higher macroscale similarity are more strongly axonally connected[9]. We therefore expected biologically optimal networks to correlate with axonal connectivity over a range of network densities, and networks with a higher AUC are more optimally capturing homophilic attachment principles.

Selecting optimal Histogram parameters using anatomical benchmarks

Changing the parameters of KL estimation can lead to large changes in resulting MIND networks (**Supplementary Figure 2**). To empirically select the optimal parameter set for downstream analyses, we anatomically benchmarked networks generated from a range of parameters. We chose to vary the percentiles of retained data and number of bins in the histogram algorithm, and the percentiles in the k-NN algorithm as these led to the largest changes in KL estimation (**Supplementary Figure 2**). The optimal parameter set for the histogram algorithm was number of bins = 32 and percentiles = [0.1, 99.9] (**Supplementary Figure 3**). Downstream analyses therefore use this parameter set. Performance of different k-NN parameter networks on anatomical benchmarks was remarkably similar, so we elected to use the default set of percentiles for downstream analyses ([0, 100]).

Cell type-specific correlated gene expression network analysis

Analysis of glutamatergic neuronal subtypes

We elected to not analyse the relationships between myeloarchitectonic similarity and glutamatergic subtype-specific correlated gene expression for two reasons: i) the cell subtype marker gene sets we used are less reliable for excitatory glutamatergic neurons, complicating the biological interpretation of subtypes of glutamatergic neurons; and ii) conducting statistical tests of association between multiple glutamatergic neuronal subtypes and MIND phenotypes would increase the severity of the adjusted false positive threshold for multiple comparisons with commensurate loss of statistical power.

Firstly, to assign cell type clusters to a biological identity, we computed the mean Z-scored expression of cell type marker gene sets obtained from the marmoset primary motor cortex[12] (**Supplementary Figure 10**). Given that primary motor cortex lacks a granular cell layer and contains glutamatergic neuronal subtypes with unique

morphologies (e.g. Betz cells), we expected that the glutamatergic marker gene sets may not generalise to other cortical areas.

Secondly, though glutamatergic neurons show transcriptomic diversity across the cortex[13], we observed that glutamatergic subtype-specific correlated gene expression tended to be more highly collinear (mean pairwise r = 0.75), than interneurons (mean pairwise r = 0.61). We reasoned that these glutamatergic subtypes would be similarly associated with MIND phenotypes and their inclusion in the full analysis would worsen the multiple comparisons problem and reduce power to detect associations between myeloarchitectonic similarity and interneuronal gene co-expression.

Analysis of oligodendrocyte subtypes

Given that the principal phenotype of interest in our study is myelination, we elected to additionally investigate the relationships between oligodendrocyte subtype gene co-expression and MIND phenotypes. Oligodendrocyte cluster 1 mapped onto mature oligodendrocytes (ODCs) while cluster 2 mapped onto oligodendrocyte precursor cells (OPCs). Cluster 3 included only 0.05% of total oligodendrocytes, so was excluded. We repeated the correlation analysis in **Figure 3**, this time using these oligodendrocyte subtypes instead of interneuron subtypes.

ODC gene co-expression was correlated with T1w/T2w MIND (r = 0.10, p = 0.531) and with *MBP* MIND (r = 0.12, p = 0.340); while OPC gene co-expression was more strongly correlated with T1w/T2w MIND (r = 0.17, p = 0.486) and with *MBP* MIND (r = 0.27, p = 0.144). However, none of these correlations were significant when accounting for spatial autocorrelation. Although provisional, this pattern of results is biologically plausible. OPCs proliferate and differentiate in response to neuronal activity[14–16], and therefore undergo transcriptional changes in response to activity in local neuronal circuits. Whereas, ODCs are terminally differentiated, so reflect a more transcriptionally stable cellular state. This may result in enhanced transcriptional coupling between OPC gene expression and myeloarchitecture relative to mature ODCs. Definitive testing of this hypothesis remains a focus for future studies which are sufficiently powered.

Non-linear analyses

Previous literature in humans has suggested that myelination continues to increase beyond the onset of adulthood, into the 30s or 50s[17]. To assess whether marmosets display protracted increases in myelination beyond the accepted onset of sexual maturity at 18-24 months[18], we fit non-linear models to regional mean T1w/T2w, regional MIND weighted degree, and MIND edge weights across the lifespan to identify timepoints of significant change. We used generalized additive models (GAMs) to flexibly capture developmental trajectories without imposing a predefined polynomial form, implemented using the *mgcv* package in R[19]. Because spline smoothness selection becomes unstable in age ranges with sparse data[20], we excluded animals falling in age ranges with a sampling density below 5%, leaving N = 416 animals (N female = 223), aged 0.62-10.67 years.

To determine whether lifespan changes were better characterised as linear or non-linear, we fit the following model:

$$Y \sim Age + s\big(Age,\, k = 10,\, m = c(2{,}0)\big) + Sex \tag{3}$$

Specifying $m = c(2{,}0)$ removes the constant and linear components of the smooth term, such that it captures only non-linear changes. Linear and non-linear age effects are therefore estimated simultaneously but remain statistically separable. A significant smooth term indicates that the trajectory deviates significantly from a straight line, reflecting the presence of non-linear effects beyond the linear age term[20]. For features with a significant smooth term, we fit a full GAM and extracted ages at which key milestones were reached. For each feature, we fit the following model:

$$Y \sim s(Age,\, k = 10) + Sex \tag{4}$$

Example GAM fits are shown for the left primary auditory cortex mean T1w/T2w (**Supplementary Figure 11A**), weighted degree (**Supplementary Figure 11B**), and an example edge (**Supplementary Figure 11C**). To quantify the rate of change across the lifespan, we computed the first derivative of the fitted curve using the method of finite differences using R's *gratia* package[21]. Age ranges in which the confidence intervals of the first derivative do not contain 0 can be thought to be significantly changing[22]. First derivative curves are shown for left primary auditory cortex mean T1w/T2w (**Supplementary Figure 11D**), weighted degree (**Supplementary Figure 11E**), and an example edge **(Supplementary Figure 11F**), with significantly changing age ranges highlighted. We defined the maximum age bound of the first significantly changing age range as the age of plateau for that feature. For each feature set, the distribution of age at plateaus is shown in **Supplementary Figure 11G-I**. Regional mean T1w/T2w values plateau before 3 years of age (median age = 2.29), while MIND weighted degree and edge weights continue changing beyond 3 years, though the median age at plateau in both cases is approximately 2.5 years. Significant plateauing in edge-wise age-related changes may therefore contribute to the qualitative plateau in edge-wise correlations between age-binned networks in **Figure 5A**.

Rationale for linear modelling of developmental changes

We modelled developmental changes using linear models for two reasons. First, linear models are easily interpretable, with a single beta coefficient capturing the slope of change across development. Second, *MBP* expression data were available at only two timepoints, with developmental change approximated as the difference in mean regional values or MIND network properties between an infant (6 months) and adult (4 years) animal. Modelling T1w/T2w trajectories linearly therefore enabled direct cross-modal comparison with *MBP* expression changes, providing a transcriptomic validation of MRI-derived developmental findings.

To statistically assess whether developmental changes were better characterised by linear or non-linear models, we used the model specified in equation (3), which allowed linear and non-linear age effects to be estimated simultaneously and parsed statistically. Developmental analyses used an upper age bound of 3 years, by which

point regional T1w/T2w values had stopped significantly increasing and most MIND network weighted degrees and edge weights had stopped significantly changing (**Supplementary Figure 11G-I**). Although most regional mean T1w/T2w trajectories were better described by non-linear models (**Supplementary Table 3**), MIND network properties were more equally well-fit by linear and non-linear models. This suggests that while linear modelling is a simplification of the true developmental T1w/T2w trajectories, it more faithfully captures developmental changes in MIND network properties.

Supplementary Data

Supplementary Data 1 (data contained in separate excel file): Regions used in whole-cortex analysis. *Table showing the 115 cortical regions per hemisphere used in our analyses. The amygdalo-piriform area of the Paxinos et al.[23] parcellation was not included in our analyses as it is absent in the BMA 2019 atlas used to parcellate MRI images. Additional anatomical data (cortical zone, cytoarchitectonic class, and position in cortical hierarchy) is listed for all cortical areas for which data is available. Whilst we are using the nomenclature used by Paxinos et al.[23] for cortical zonal parcellations of marmoset prefrontal cortex, it should be noted that the division of dorsolateral prefrontal cortex extends into regions typically named dorsomedial prefrontal cortex in humans[24].*

Supplementary Tables

**Estimator type**	**Estimator name**	**Parameter name**	**Parameter description**	**Default parameter value**
Global	Histogram	Number of bins	Number of bins into which to partition the data	128
		Epsilon	Value added to the denominator in Equation (2), when the bin in distribution B is empty, i.e. when $p_b(x) = 0$. This avoids division by 0 errors.	2.2204e-16 (machine epsilon)
		Percentiles	Percentiles of voxel values to retain. This parameter may be tuned to reduce the influence of outlier values, which increase the range over which bins are stretched and therefore influence the presence of empty bins.	[1, 99]
Local	k-NN	*k*	The kth nearest neighbour used to compute local distance in equation (2).	1
		Percentiles	Percentiles of voxel values to retain. This parameter may be tuned to reduce the influence of outlier values.	[0, 100]

Supplementary Table 1: Tuneable parameters in KL estimators. *Tuneable parameters in Histogram and k-NN algorithms, including values used in the default case and a parameter description.*

Coarse region abbreviation	Coarse region full name	Paxinos subregions abbreviation
DLP	Dorsolateral prefrontal cortex	A10, A46D, A46V, A8aD, A8aV, A8b, A9
VLP	Ventrolateral prefrontal cortex	A45, A47L, A47M, A47O, ProM
MPC	Medial prefrontal cortex	A14C, A14R, A32, A32V
OFC	Orbitofrontal cortex	A11, A13L, A13M, A13a, A13b, Gu, OPAl, OPro
LTC	Lateral temporal cortex	TPO, TPPro
LPC	Lateral parietal cortex	OPt, PFG
PCA	Posterior cingulate cortex, area 23	A23V, A23a, A23b, A23c
M1	Primary motor cortex	A4ab, A4c
S1	Primary somatosensory cortex	A1-2, A3a, A3b
A1	Primary auditory cortex	AuA1
V1	Primary visual cortex	V1
V2	Secondary visual cortex	V2

Supplementary Table 2: Coarse-grained parcellation used in single cell transcriptomic analyses*. Coarse-grained cortical parcellation used in single cell transcriptomic analyses and constituent regions. For each of these 12 coarse-grained cortical areas, cells of each subtype were sampled from more than one marmoset, representing both sexes, except for the posterior cingulate cortex, A23, which was sampled in a single male. In total, these cortical data were sampled from 6 marmosets aged from 29 to 34 months, except for one older animal (11 years, 5 months) which contributed one sample from the dorsolateral prefrontal cortex. Given that both sexes were sampled, and predominantly from a constrained age range, transcriptional differences between cortical areas were considered unlikely to be driven mainly by age or sex.*

Feature	% features best fit by linear age term	% features best fit by non-linear age term	% features with no significant age-related change
Mean T1w/T2w	3	90	7
MIND degree	15	30	55
MIND edges	26	20	54

Supplementary Table 3: Features best fit by linear and non-linear models*. Percentage of features best fit by models with a linear age term, non-linear age term, or showing no significant age-related change between pre-adolescence and adulthood.*

Supplementary Figures

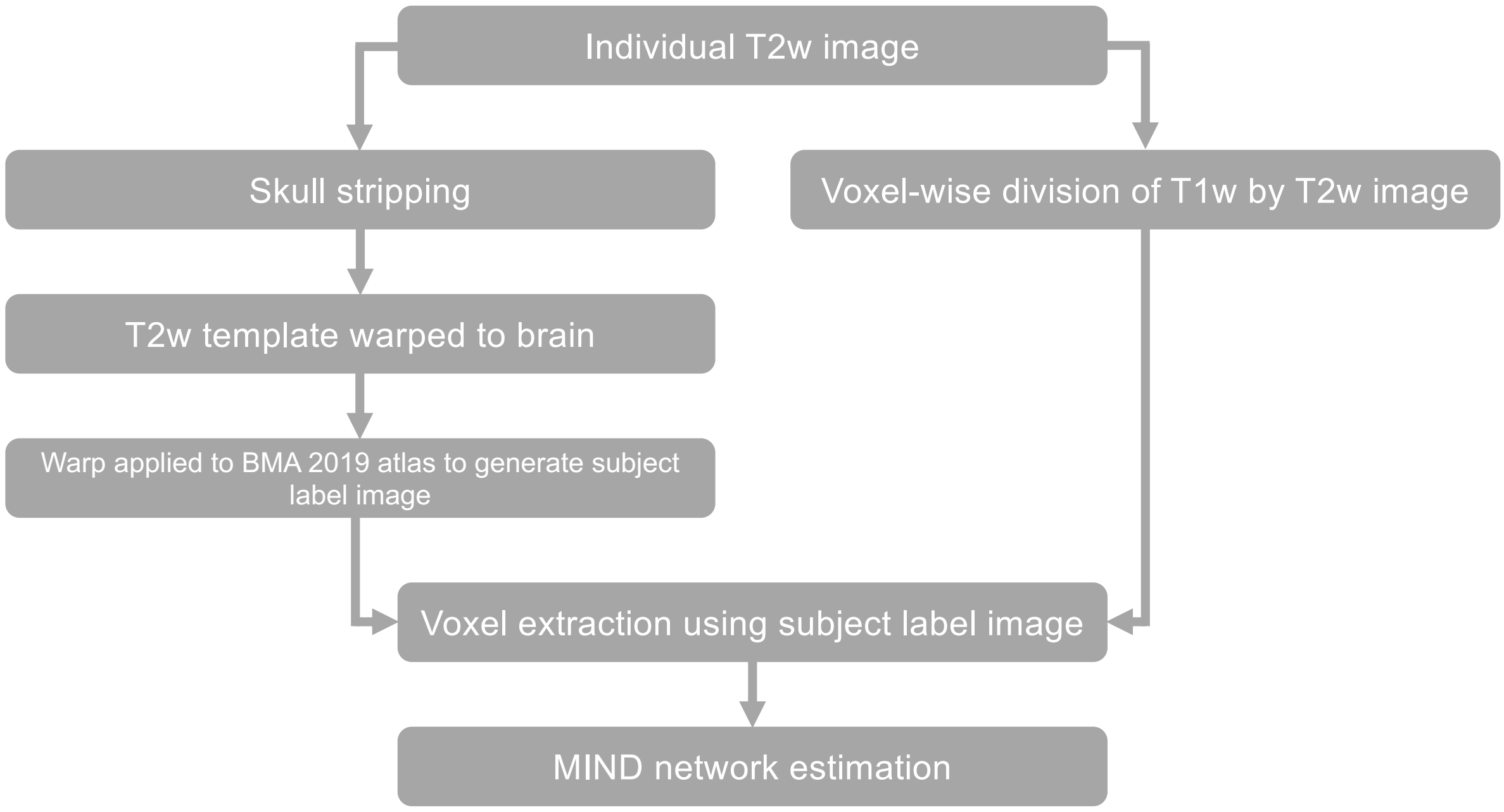

Supplementary Figure 1: Preprocessing of T1w/T2w ratio images. *Schematic showing preprocessing of T1w/T2w ratio MRI images.*

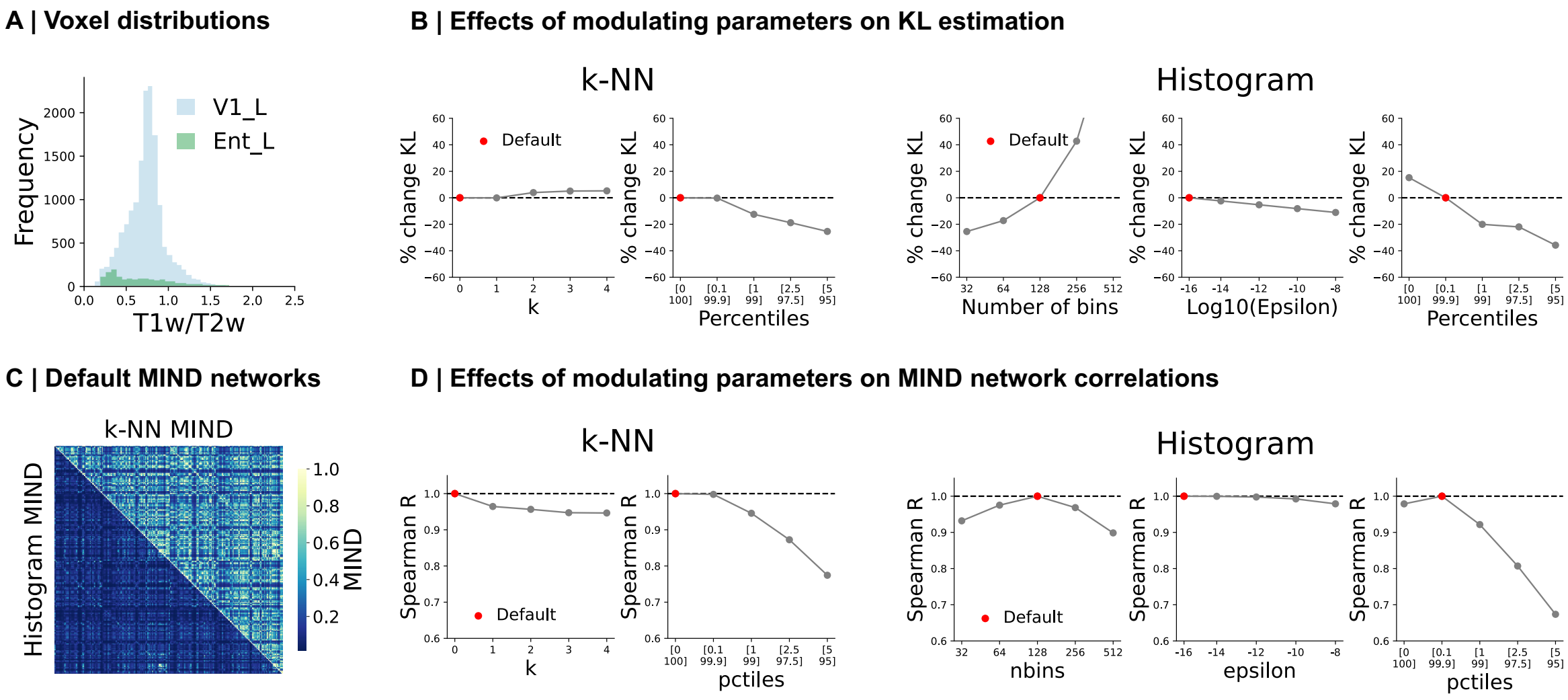

***Supplementary Figure 2: Edge-level effects of tuning parameters in KL divergence estimators. A** Voxel distributions from two regions of a representative adult T1w/T2w ratio image (V1_L: left primary visual cortex; Ent_L: left entorhinal cortex). KL divergence was computed between this pair of regions for a range of algorithm parameter values. **B** Changing all parameters leads to changes in KL, indicated by the percentage change from default (marked in red). **C** MIND similarity networks estimated with default parameters. **D** Changing all parameters leads to a change in network structure, indicated by a Spearman's rho correlation < 1, from default (marked in red). Changes in **B** and **D** are most notable for k-NN percentiles, Histogram percentiles and number of bins.*

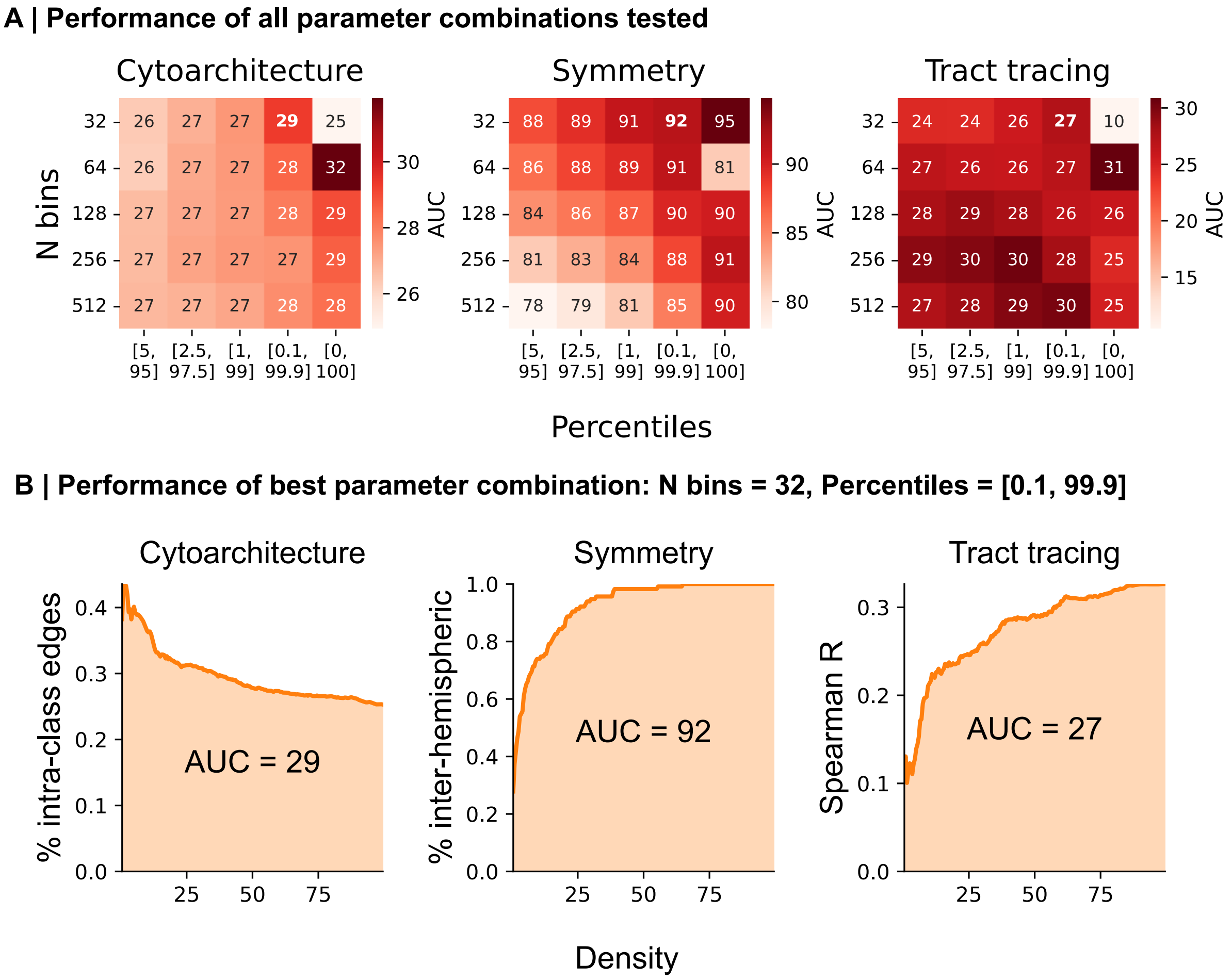

Supplementary Figure 3: Selecting optimal global algorithm parameters. A *Performance of Histogram MIND networks, estimated using all combinations of number of bins and percentiles, on biological benchmarks.* ***B*** *Performance of the optimal Histogram MIND parameter set (Number of bins = 32, percentiles = [0.1, 99.9]), defined as having the highest mean rank performance across all 3 benchmarks in* ***A****.*

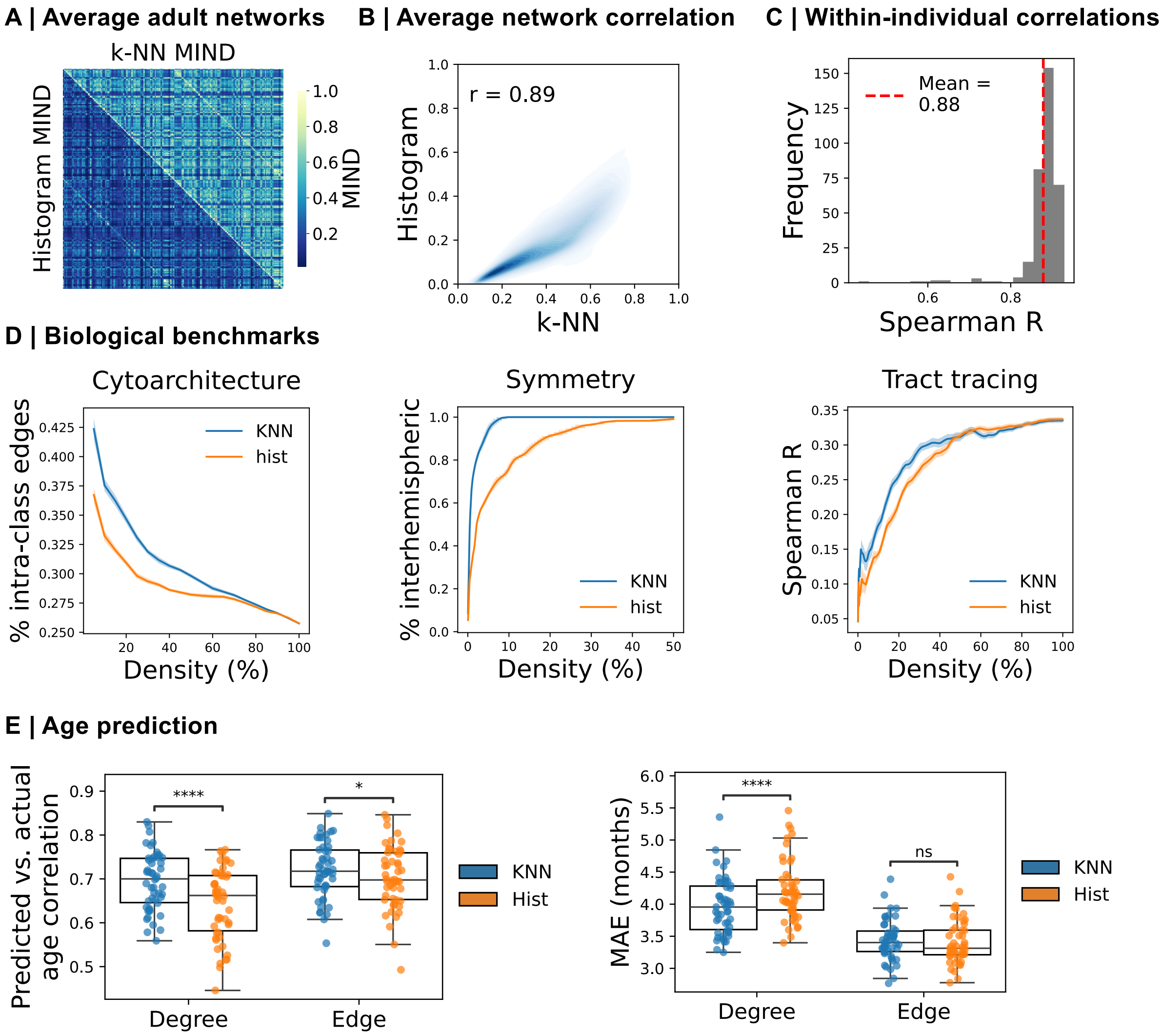

Supplementary Figure 4: Local algorithm out-performs global on anatomical benchmarks and age prediction. ***A*** *Average adult k-NN MIND and optimised Histogram MIND networks.* ***B*** *Average networks in* ***A*** *are highly correlated at the edge level (Spearman r = 0.89).* ***C*** *Networks of individual subjects are highly correlated at the edge-level (mean Spearman r = 0.88).* ***D*** *k-NN MIND out-performs Histogram MIND on cytoarchitectonic, symmetry, and tract tracing benchmarks, indicated by a higher AUC. The shaded areas indicate 95% confidence intervals estimated by subject-level bootstrapping (N = 500 bootstraps).* ***E*** *k-NN MIND out-performs Histogram MIND at age prediction, indicated by a significantly higher median partial correlation between predicted and true age at the degree and edge level, and a significantly lower median MAE at the degree level over 50 splits of the data. Asterisks indicate significance of paired Wilcoxon signed-rank tests: * p < 0.05, ** p < 0.01, *** p < 0.001, **** p < 0.0001.*

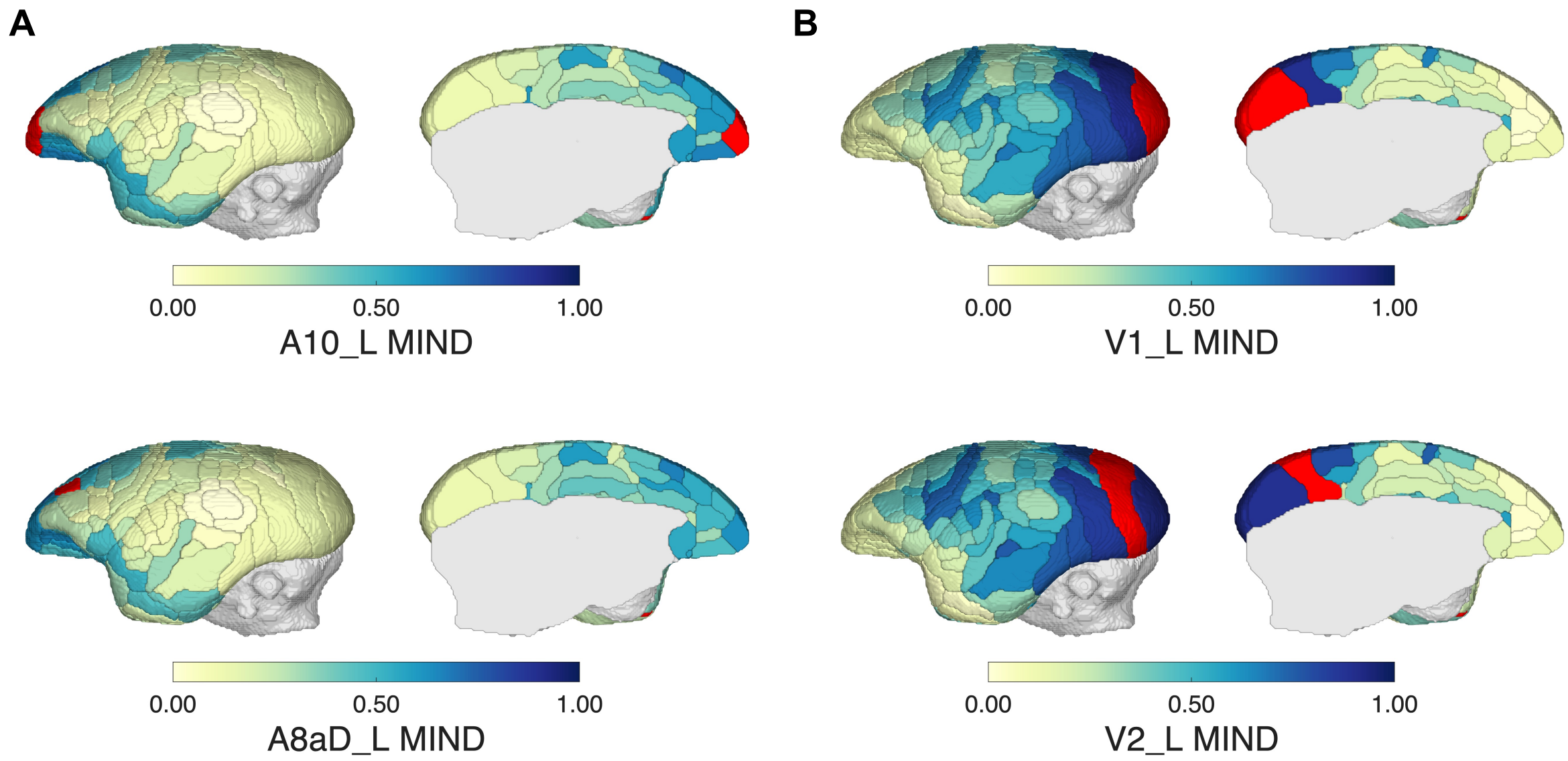

Supplementary Figure 5: Seed-based MIND maps for the mean adult T1w/T2w MIND network. *Each map shows the MIND similarity between a seed region, highlighted in red, and other cortical regions in the mean adult T1w/T2w MIND network (**Figure 1**). The MIND network was highly symmetric, so only left hemispheric regions are shown for simplicity. Example seeds were chosen from two regions in the **A** dorsolateral prefrontal cortex and **B** primary visual cortex to exemplify how patterns of similarity tend to diverge between association and primary sensory regions.*

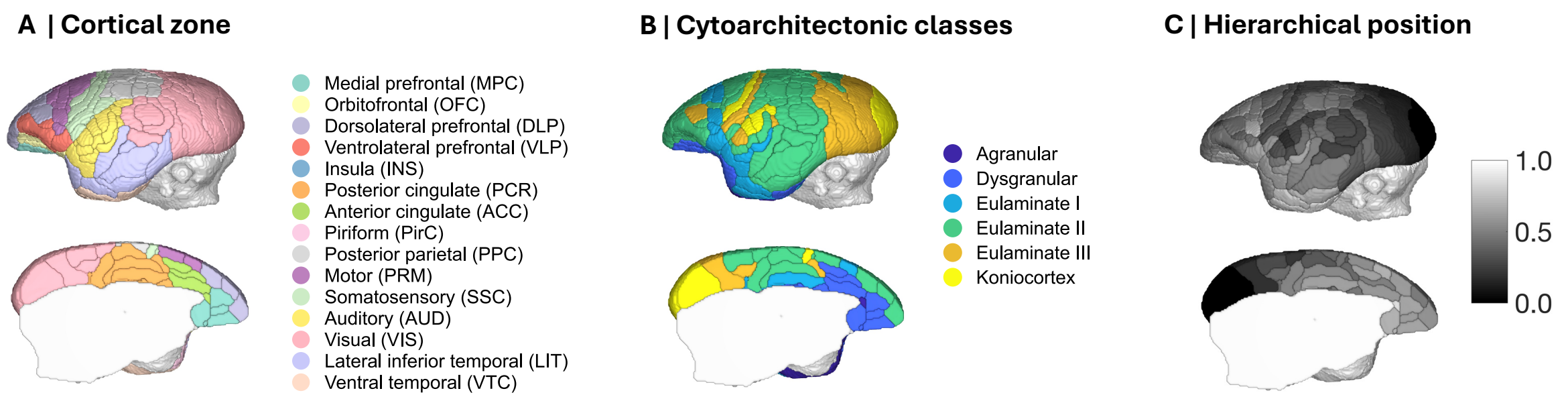

***Supplementary Figure 6: Anatomical maps. A** Anatomical and functional cortical zones from Paxinos et al. [23]. **B** Cytoarchitectonic class assignment map from Atapour et al.[25]. **C** Hierarchical position map from Theodoni et al.[26]. For a full list of cortical areas used in our analyses, alongside their assigned cortical zones, cytoarchitectonic classes, and hierarchical positions, see **Supplementary Data 1**.*

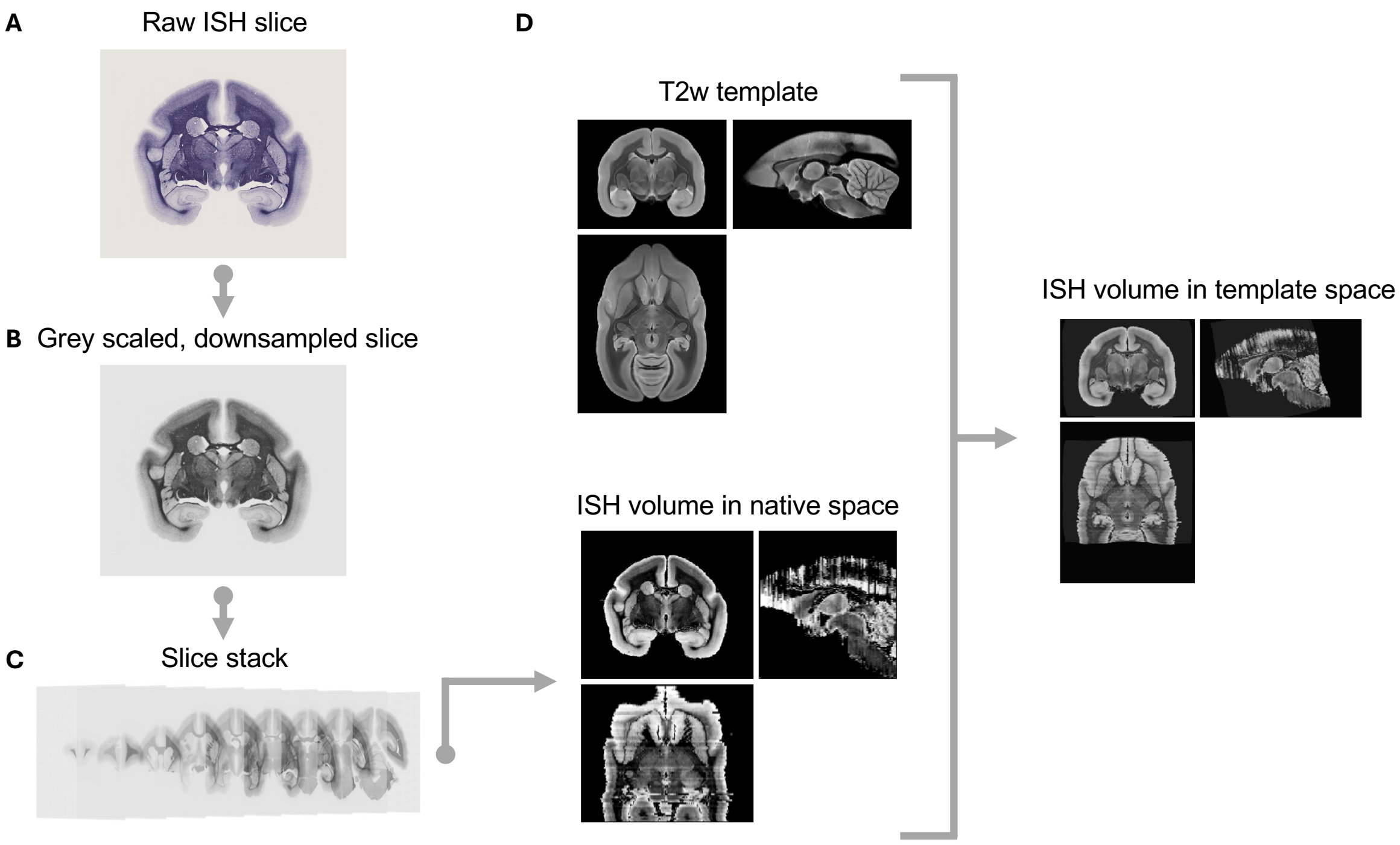

Supplementary Figure 7: Preprocessing of in-situ hybridisation images. *We downloaded in-situ hybridisation images capturing the spatial distribution of myelin basic protein mRNA in a 6-month-old male and 4-year-old male marmoset from the Marmoset Gene Atlas[27,28]. In each brain, MBP gene expression was measured in 90 coronal brain slices, which were used to generate an in-situ hybridisation (ISH) volume that was then co-registered to MRI space. Preprocessing of slices used Matlab and ANTs.* ***A*** *Example raw slice.* ***B*** *Full resolution slices were downsampled and grey-scaled to reduce computational load.* ***C*** *Slices were roughly aligned to create a native ISH volume using code from Tong et al.[29].* ***D*** *The ISH volume was linearly and non-linearly warped to the same ex-vivo T2w template used in the preprocessing of MRI images[30]. Slices were then inverted to match contrast of T1w/T2w images (not shown).*

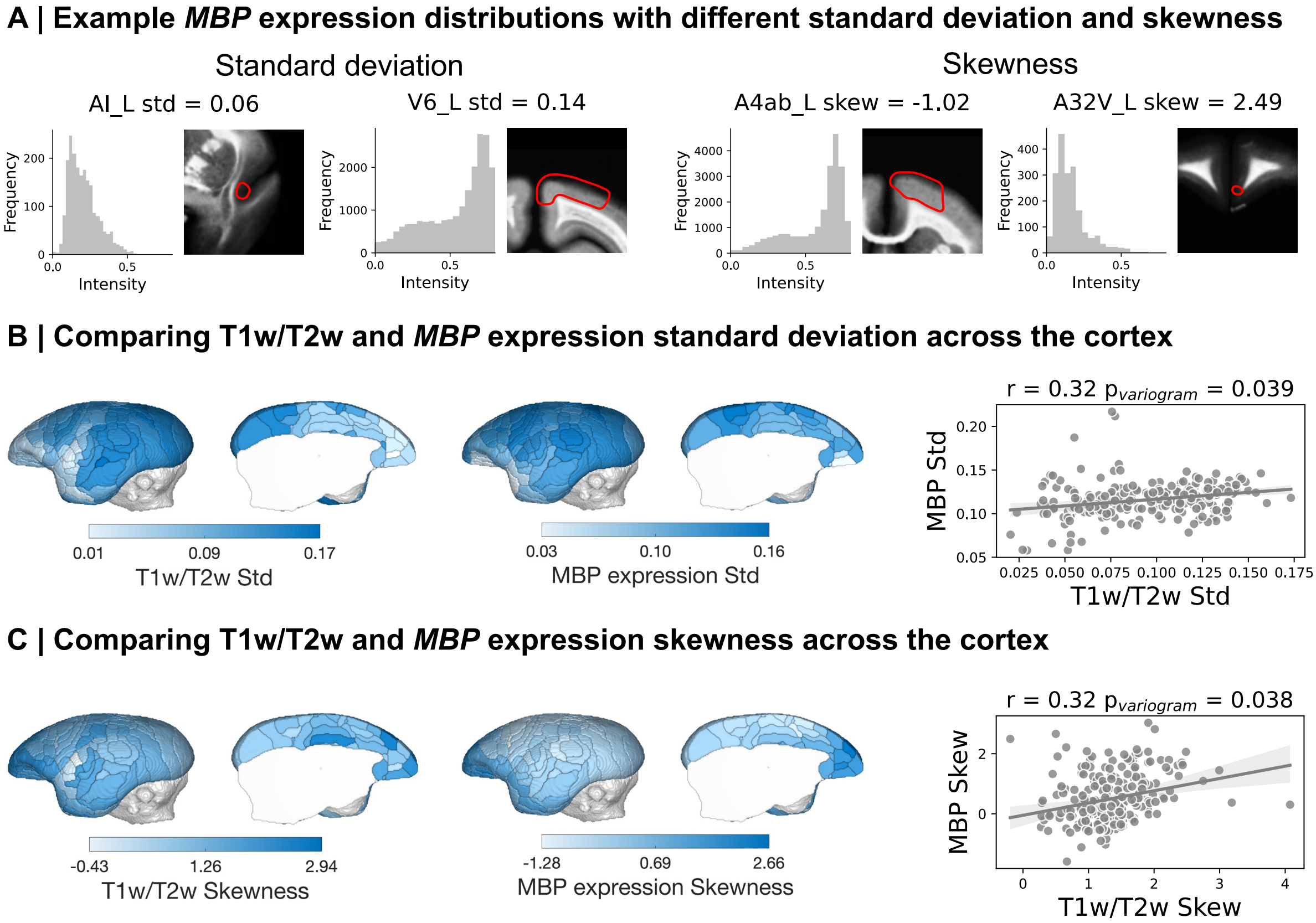

Supplementary Figure 8: Comparing T1w/T2w and MBP expression distribution shapes. A *Example regions from MBP expression data with low and high standard deviation (left panel) and skewness (right panel). Negative skew indicates that the distribution tail falls to the left, and positive skew to the right.* ***B*** *Standard deviations of T1w/T2w and MBP expression distributions are moderately correlated (Spearman r = 0.32,* $p_{variogram}$ *= 0.032).* ***C*** *Skewness of T1w/T2w and MBP expression distributions are weakly correlated (Spearman r = 0.40,* $p_{variogram}$ *= 0.003).*

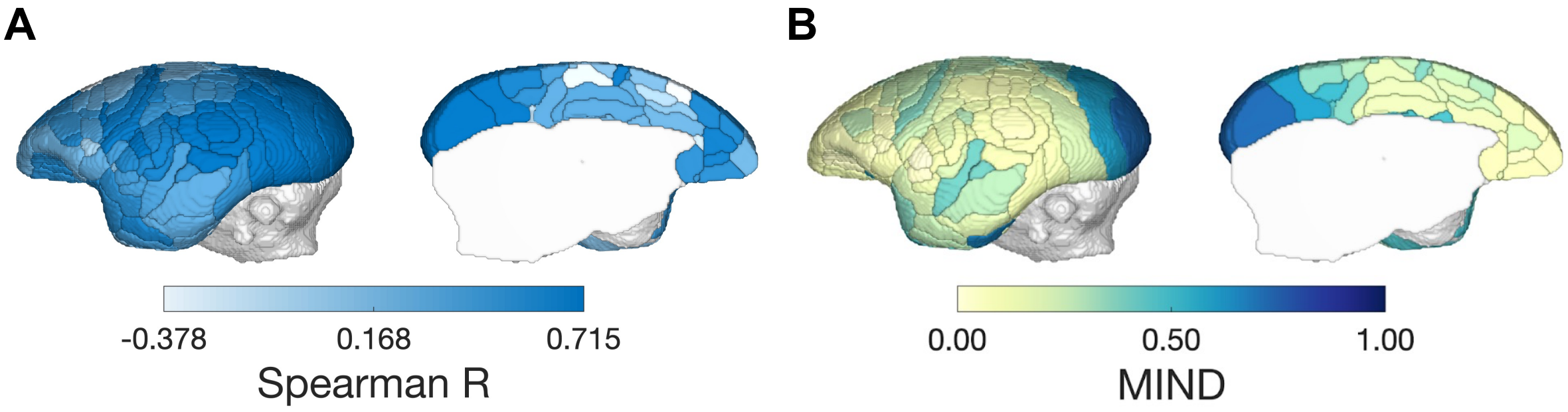

Supplementary Figure 9: Cross-modal concordance of MIND edges and cross-modal similarity of signal intensity distributions. A *Map showing correlation between each cortical area's T1w/T2w MIND edges and MBP MIND edges (**Figure 2E**). **B** Map showing MIND similarity values directly computed between the T1w/T2w ratio and MBP expression signal intensity distributions for each cortical region in the left hemisphere. Resulting maps were poorly correlated (r = -0.17, p = 0.32), indicating that region-wise cross-modal concordance in MIND edge weights reflects not only the distribution properties of those regions but their relationships to all other cortical areas.*

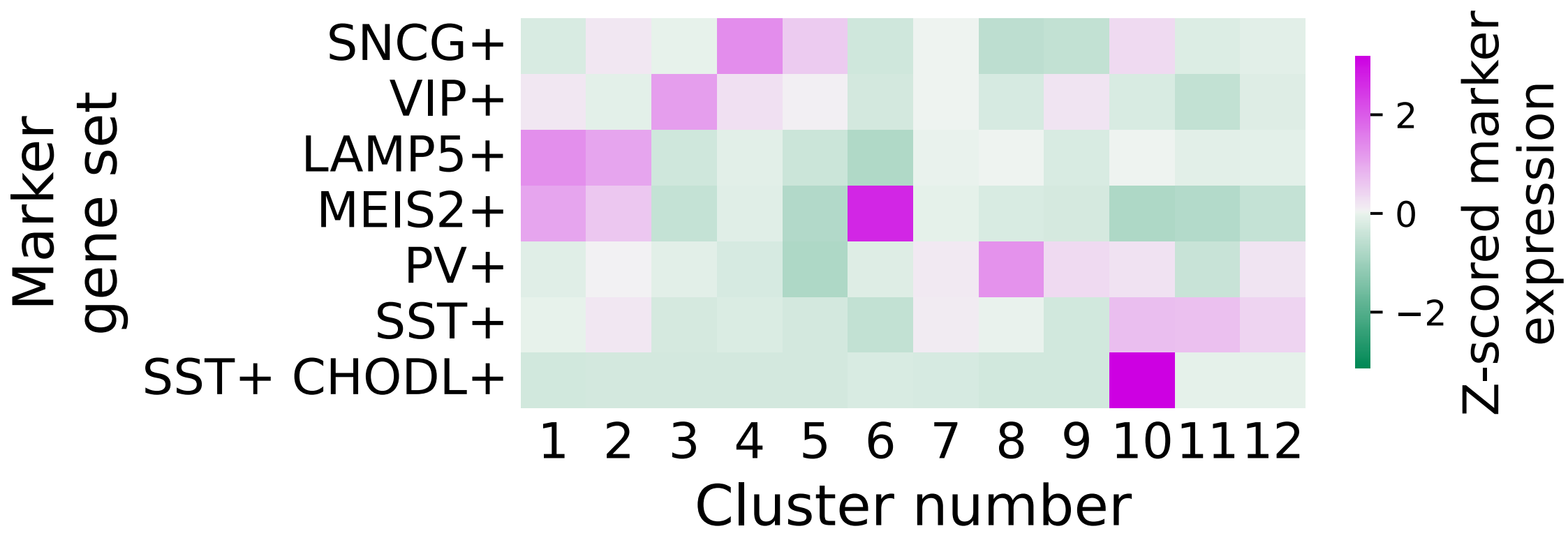

Supplementary Figure 10: Assignment of interneuron clusters to subtypes. *Marker gene sets generated from marmoset primary motor cortex samples[12] were used to assign interneuron clusters to a biological identity. The mean log expression of each marker gene was computed for each interneuron cluster. Expression of marker genes was standardised by Z-scoring across cell types. The mean Z-scored expression of each marker gene set (shown in the figure panel) was computed for each interneuron cluster, which was used to assign each cluster to a single biological identity. We assigned clusters 1 and 2 to LAMP5+, cluster 3 to VIP+, clusters 4 and 5 to SNCG+, cluster 6 to MEIS2+, clusters 7, 11, and 12 to SST+, clusters 8 and 9 to PV+, and cluster 10 to SST+ CHODL+. Clusters 5 and 6 were excluded from downstream analyses as expression was not present in the 12 cortical areas that formed the focus of our analyses.*

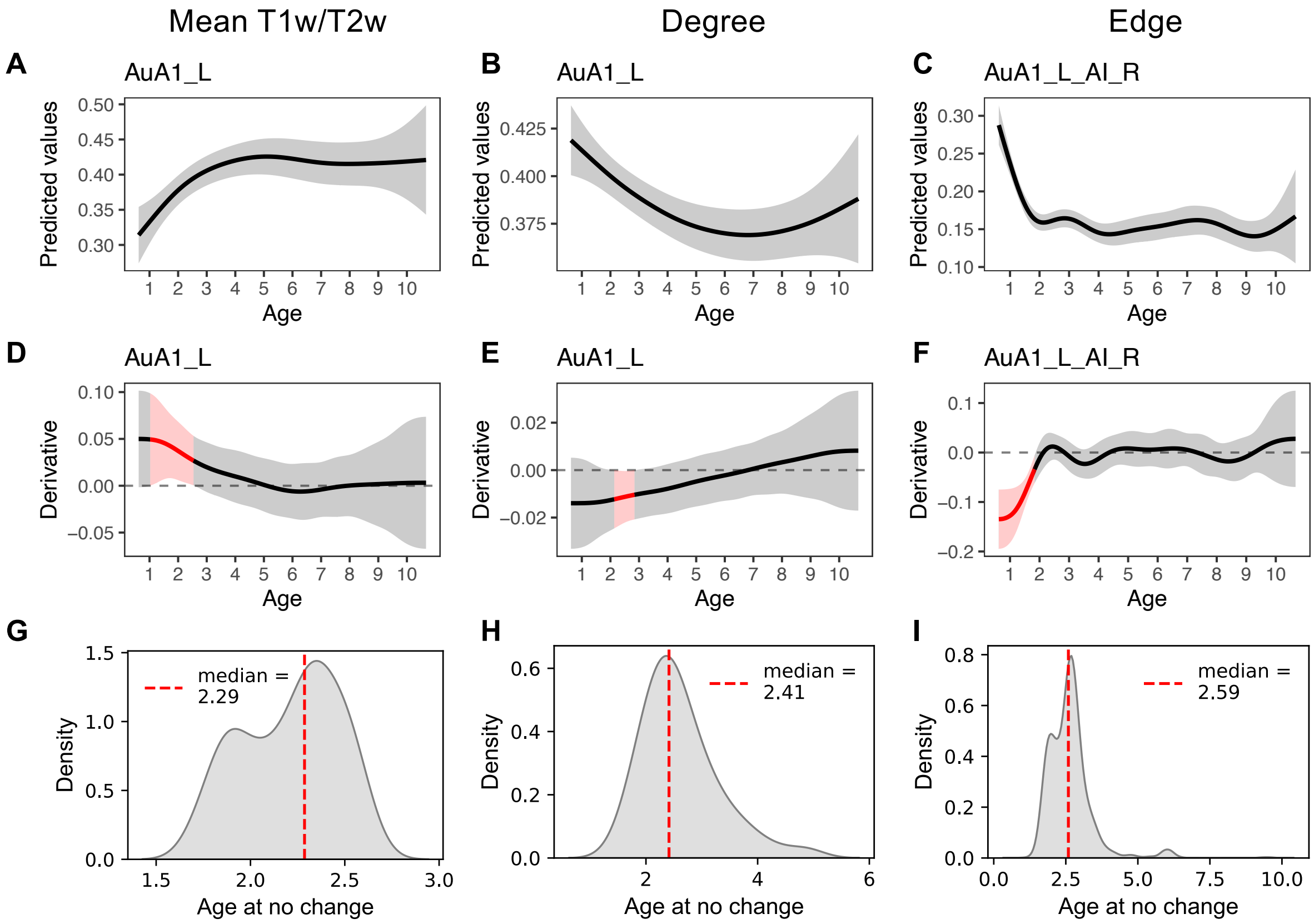

Supplementary Figure 11: Feature values commonly plateau between 2 and 2.5 years. *Example GAM fits for:* ***A*** *the left primary auditory cortex (AuA1_L) mean T1w/T2w,* ***B*** *AuA1_L weighted degree and* ***C*** *MIND edge weight between AuA1_L and the right agranular insula (AI_R).* ***D-F*** *First derivative curves for the GAM model fits in* ***A-C*** *with significantly changing age ranges highlighted in red.* ***G-I*** *Distributions of plateau ages for each set of features. Median plateau age is highlighted in red.*

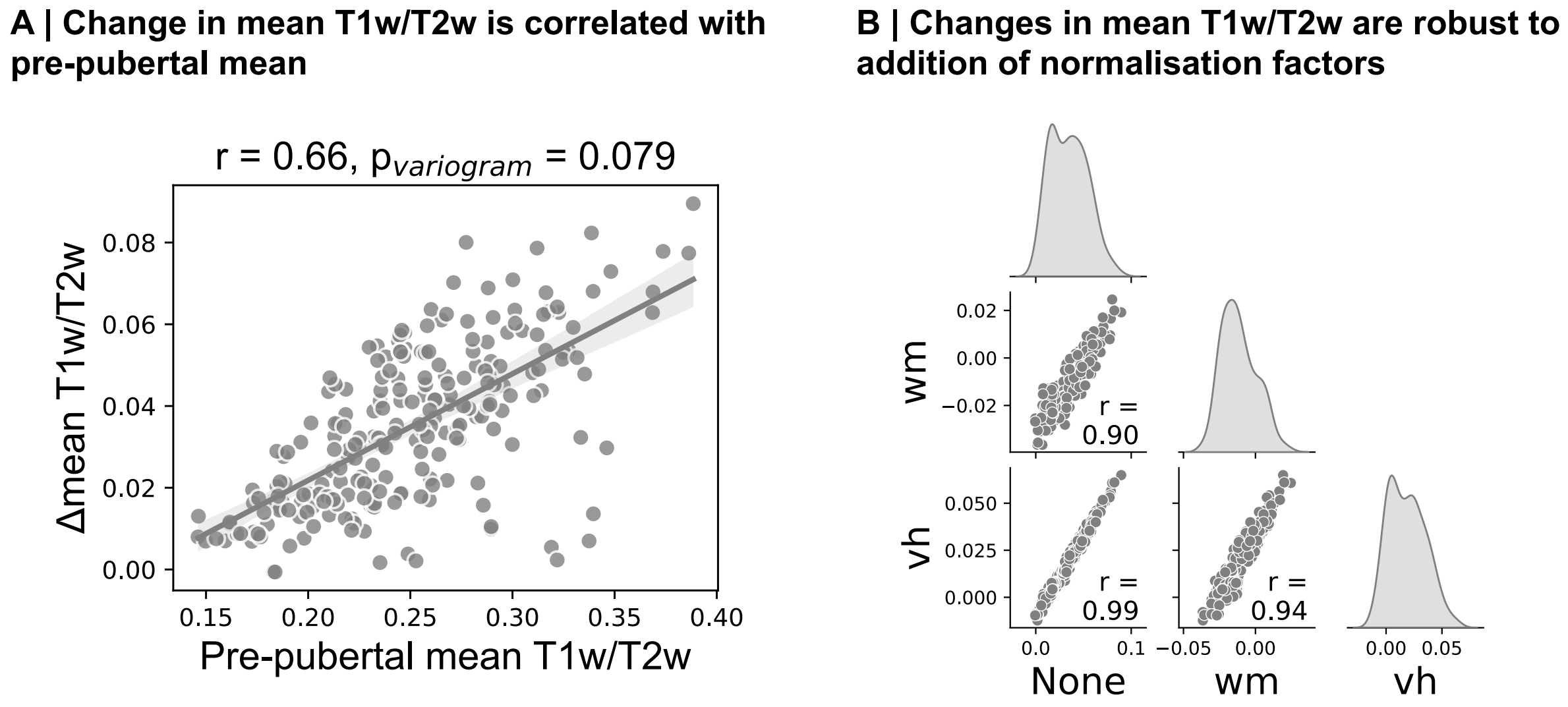

Supplementary Figure 12: Age-related changes in T1w/T2w. A *Pre-pubertal mean T1w/T2w, obtained by averaging across animals <1 year of age, was correlated with a region's adolescent increase in mean T1w/T2w, though this trend did not reach significance after accounting for spatial autocorrelation (Spearman r = 0.66,* $p_{variogram}$ *= 0.079).* ***B*** *Sensitivity analysis demonstrating that addition of normalisation covariates to linear models does not greatly alter estimates of age effects on mean T1w/T2w, indicated by high correlations between* β_{age} *coefficients for the linear models: Mean T1w/T2w ~ Age + Sex + X, where X is a normalisation covariate: None, median white matter signal (wm) or median vitreous humour signal (vh).*

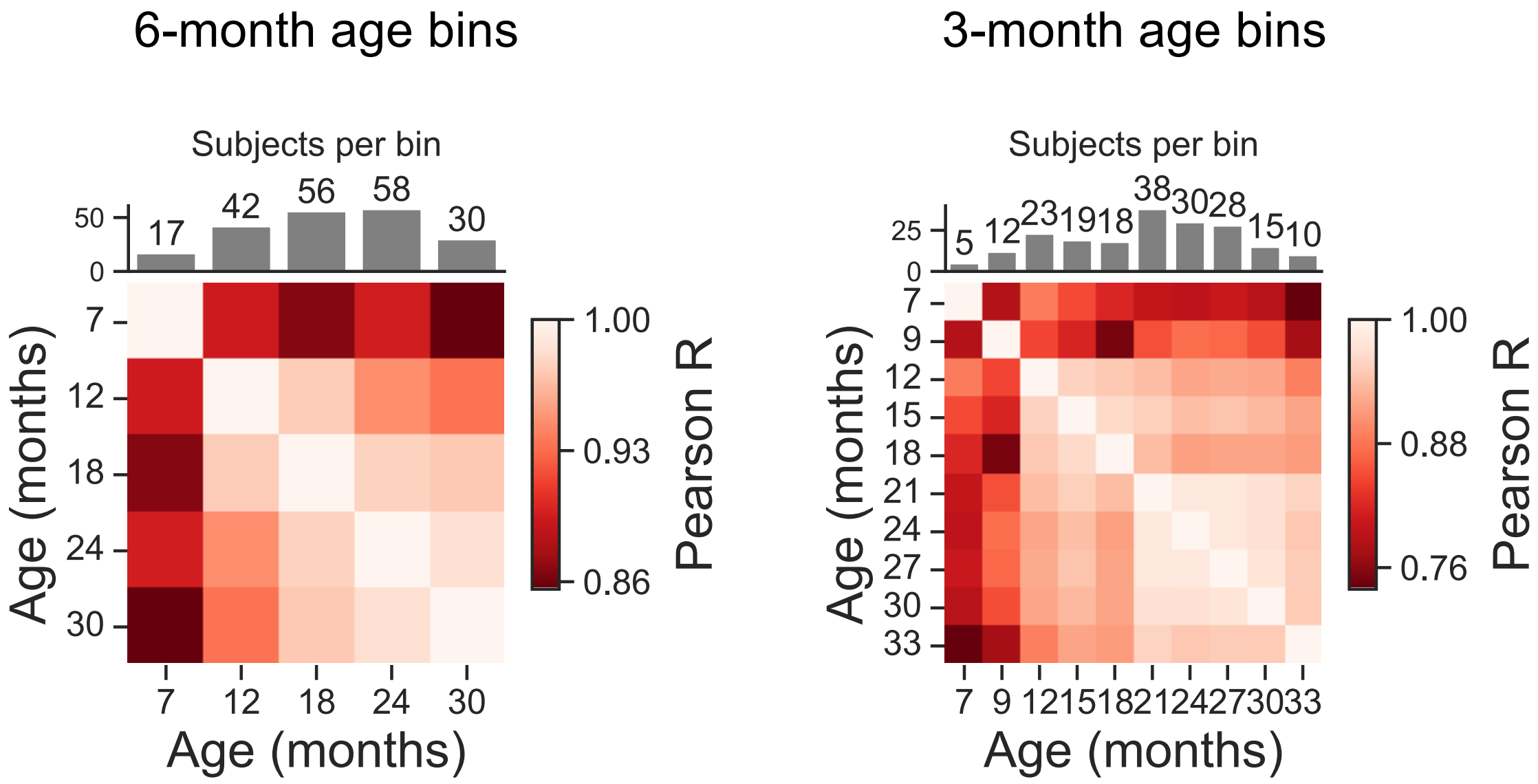

Supplementary Figure 13: Adult maturational plateau is robust to bin size*.* *Correlations between age-binned networks remain high after 18 months, when using 6-month age bins, or 21 months, when using 3-month age bins (note different colour bar scales). This indicates that, regardless of bin size, intracortical myelination networks exhibit a slowing of maturational changes close to the onset of adulthood at ~20 months[18].*

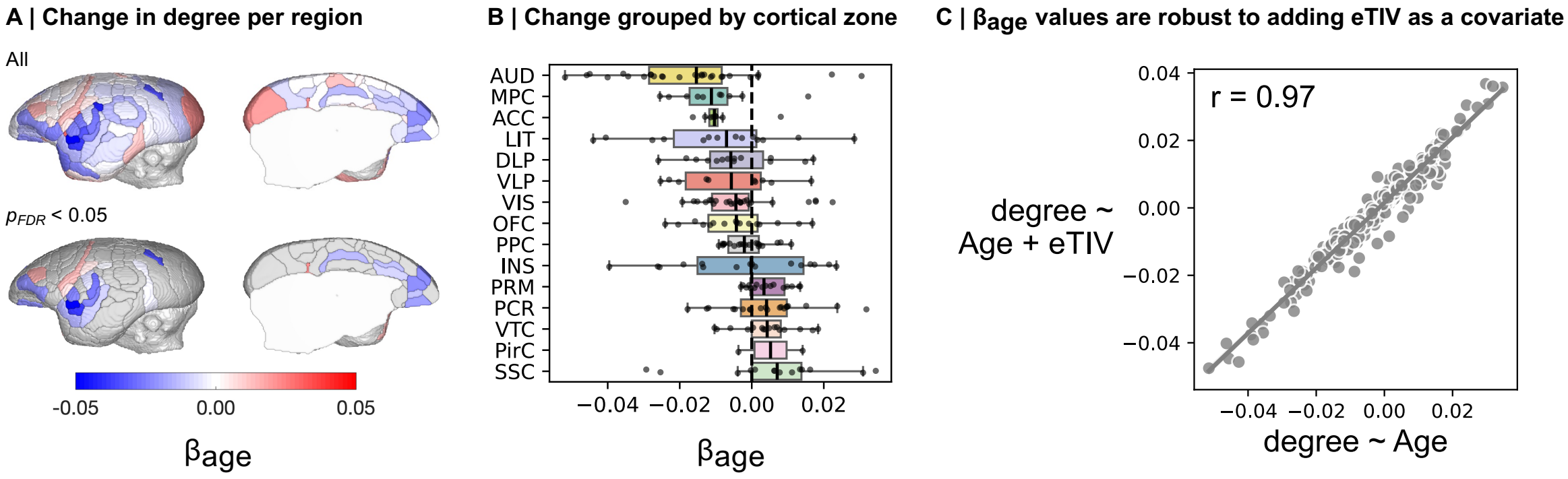

Supplementary Figure 14: Auditory regions become microstructurally differentiated from the rest of the cortex. A *Multiple linear regressions predicting degree from age and sex.* β_{age} *coefficients for the left hemisphere, uncorrected and corrected for multiple comparisons, are shown.* ***B*** *Uncorrected* β_{age} *coefficients grouped by cortical zone.* ***C*** β_{age} *coefficients are highly correlated when including or excluding estimated total intracranial volume as a covariate.*

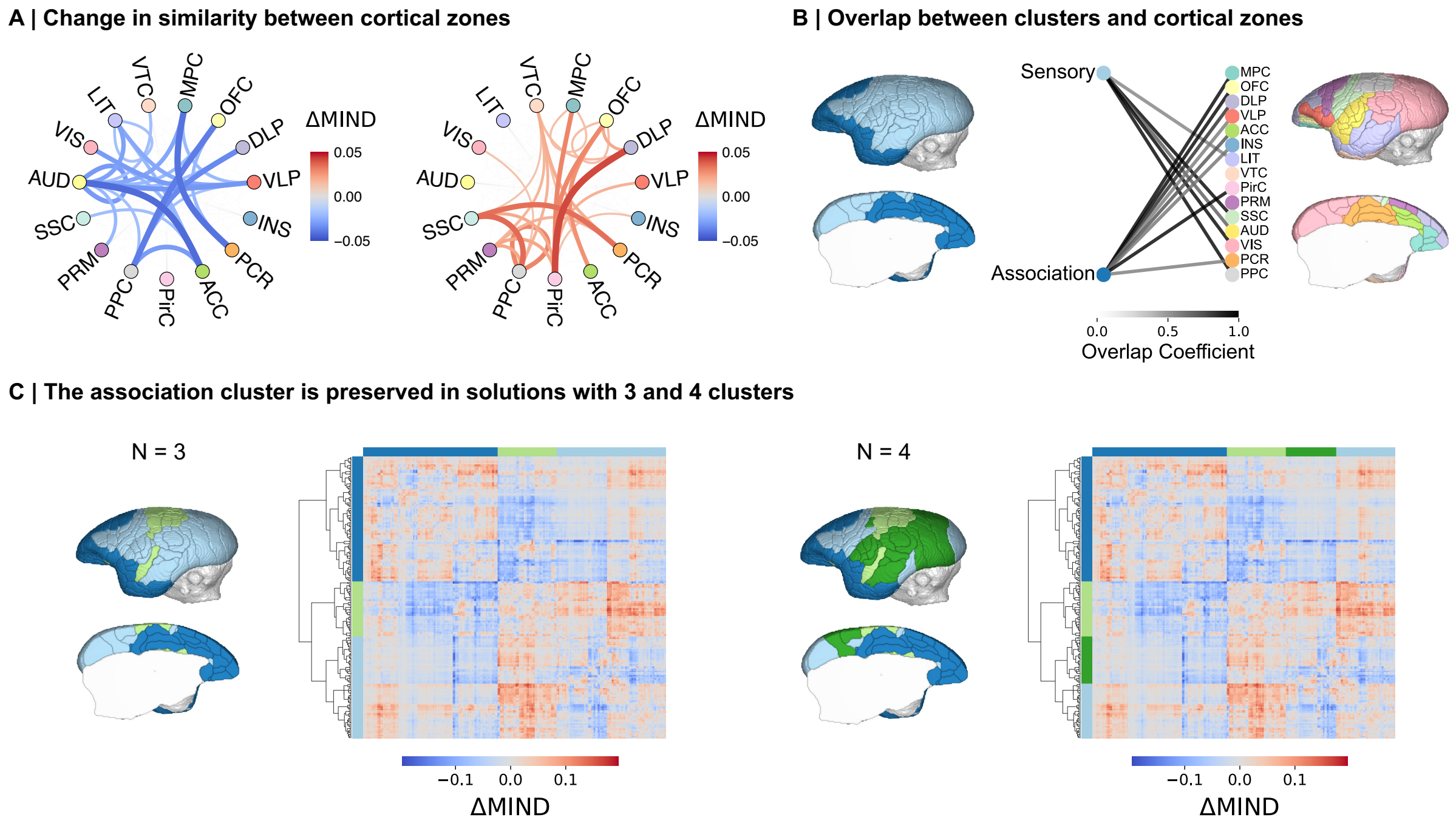

Supplementary Figure 15: Maturational clustering sensitivity analyses: ***A*** *Mean rate of change in T1w/T2w MIND between cortical zones of the left hemisphere. The bottom 20% (left) and top 20% (right) of mean changes are shown.* ***B*** *Degree to which cortical zones are contained within each left hemisphere cluster, quantified by the Szymkiewicz–Simpson overlap coefficient[31]. Overlap equals 1 if all regions in a cortical zone are contained entirely within a cluster, and 0 if none of them are. Overlap is shown if greater than 0.5, i.e. if at least half of the areas in a cortical zone are contained within a cluster. The association cluster overlaps principally with frontal and paralimbic association areas, while the sensory cluster overlaps principally with primary sensory and motor areas.* ***C*** *The association maturational cluster is relatively preserved in three and four cluster solutions, suggesting that frontal and paralimbic association areas are particularly strongly maturationally coordinated.*

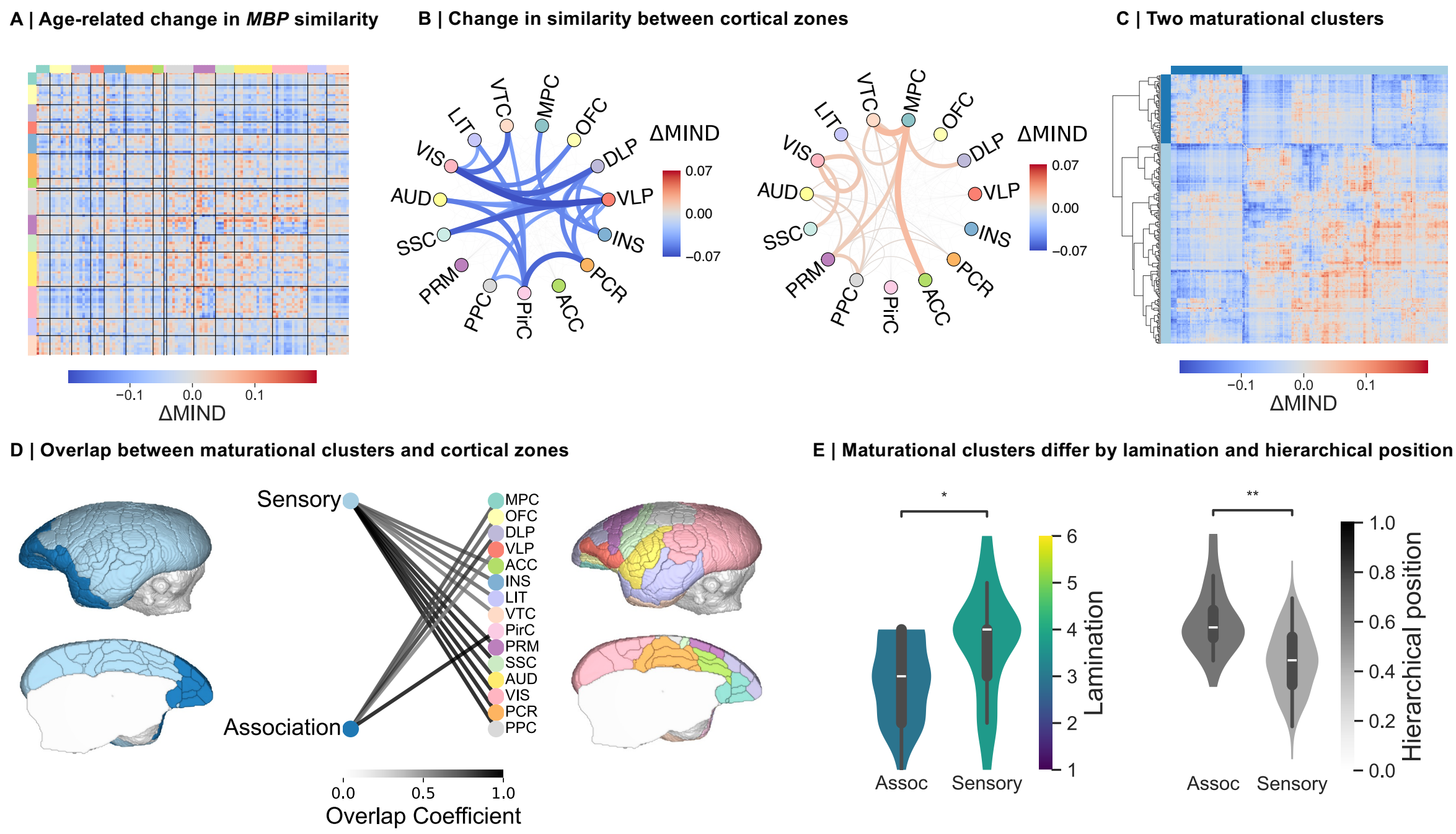

Supplementary Figure 16: Maturational changes in MBP expression similarity are concordant with changes in T1w/T2w ratio similarity. ***A*** *The difference between the adult and infant MBP expression MIND network was used to approximate linear change in similarity. Left hemisphere shown, grouped into cortical zones.* ***B*** *Left hemisphere matrix averaged across cortical zones. The bottom 20% (left) and top 20% (right) of mean changes are shown. The divergence between primary sensory (VIS, AUD, SSC) and association (OFC, DLP, VLP, PirC) cortical zones is particularly pronounced.* ***C*** *Hierarchical clustering was performed in an identical manner to the T1w/T2w MIND age-related change matrix.* ***D*** *The degree to which cortical zones were contained within each maturational cluster was quantified using the Szymkiewicz–Simpson overlap coefficient[31] (overlap > 0.5 is shown). MBP MIND development clusters were largely correspondent with T1w/T2w MIND development clusters, though the MBP MIND association cluster was smaller, containing mainly prefrontal association areas.* ***E*** *Concordant with T1w/T2w MIND development, the sensory cluster (N = 85 left hemispheric regions) had significantly higher mean degree of lamination (t = 3.72, $p_{variogram}$ = 0.010) and significantly lower mean hierarchical position (t = -3.69, $p_{variogram}$ = 0.014) than the association cluster (N = 30 left hemispheric regions). Reported p-values were derived from two-tailed, unpaired t-tests evaluated against spatially autocorrelated null models.*

Supplementary References

1. Silverman, B. W. *Density Estimation for Statistics and Data Analysis*. (Routledge, 2018).
2. Wang, Q., Kulkarni, S. R. & Verdu, S. Divergence estimation of continuous distributions based on data-dependent partitions. *IEEE Trans. Inf. Theory* **51**, 3064–3074 (2005).
3. Kong, X.-Z. *et al.* Measuring individual morphological relationship of cortical regions. *J. Neurosci. Methods* **237**, 103–107 (2014).
4. Kong, X.-Z. *et al.* Mapping individual brain networks using statistical similarity in regional morphology from MRI. *PLoS One* **10**, e0141840 (2015).
5. Wang, H., Jin, X., Zhang, Y. & Wang, J. Single-subject morphological brain networks: connectivity mapping, topological characterization and test-retest reliability. *Brain Behav* **6**, e00448 (2016).
6. Perez-Cruz, F. Kullback-Leibler divergence estimation of continuous distributions. in *2008 IEEE International Symposium on Information Theory* 1666–1670 (IEEE, 2008).
7. Sebenius, I. *et al.* Robust estimation of cortical similarity networks from brain MRI. *Nat. Neurosci.* **26**, 1461–1471 (2023).
8. Smith, R. L. *et al.* Cortical similarity networks in the rat brain: Postnatal development and sensitivity to early life stress. *Network Neuroscience* (2026) doi:10.1162/NETN.a.546.
9. Sebenius, I. *et al.* Structural MRI of brain similarity networks. *Nat. Rev. Neurosci.* **26**, 42–59 (2025).
10. Atapour, N. *et al.* Distribution of calbindin-positive neurons across areas and layers of the marmoset cerebral cortex. *PLoS Comput. Biol.* **20**, e1012428 (2024).
11. Sansom, S. N. & Livesey, F. J. Gradients in the brain: the control of the development of form and function in the cerebral cortex. *Cold Spring Harb. Perspect. Biol.* **1**, a002519 (2009).
12. Bakken, T. E. *et al.* Comparative cellular analysis of motor cortex in human, marmoset and mouse. *Nature* **598**, 111–119 (2021).
13. Krienen, F. M. *et al.* A marmoset brain cell census reveals regional specialization of cellular identities. *Sci Adv* **9**, eadk3986 (2023).
14. Barres, B. A. & Raff, M. C. Proliferation of oligodendrocyte precursor cells depends on electrical activity in axons. *Nature* **361**, 258–260 (1993).
15. Emery, B. Regulation of oligodendrocyte differentiation and myelination. *Science* **330**, 779–782 (2010).
16. Mount, C. W. & Monje, M. Wrapped to Adapt: Experience-Dependent Myelination. *Neuron* **95**, 743–756 (2017).
17. Grydeland, H. *et al.* Waves of Maturation and Senescence in Micro-structural MRI Markers of Human Cortical Myelination over the Lifespan. *Cereb Cortex* **29**, 1369–1381 (2019).
18. Sawiak, S. J. *et al.* Trajectories and Milestones of Cortical and Subcortical Development of the Marmoset Brain From Infancy to Adulthood. *Cereb Cortex* **28**, 4440–4453 (2018).
19. Wood, S. Mixed GAM Computation Vehicle with Automatic Smoothness Estimation [R package mgcv version 1.9-4]. (2025).
20. Wood, S. N. *Generalized Additive Models: An Introduction with R, Second Edition*. (CRC Press, 2017).

21. Simpson, G. L. gratia: An R package for exploring generalized additive models. *Journal of Open Source Software* **9**, 6962 (2024).
22. Simpson, G. L. Modelling Palaeoecological Time Series Using Generalised Additive Models. *Front. Ecol. Evol.* **6**, 396134 (2018).
23. Paxinos, G., Rosa, M., Merlin, S. & Majka, P. *The Marmoset Brain in Stereotaxic Coordinates*. (Academic Press, 2025).
24. Carlén, M. What constitutes the prefrontal cortex? *Science* **358**, 478–482 (2017).
25. Atapour, N. *et al.* Distribution of calbindin-positive neurons across areas and layers of the marmoset cerebral cortex. *PLoS Comput Biol* **20**, e1012428 (2024).
26. Theodoni, P. *et al.* Structural attributes and principles of the neocortical connectome in the marmoset monkey. *Cereb. Cortex* **32**, 15–28 (2021).
27. Shimogori, T. *et al.* Digital gene atlas of neonate common marmoset brain. *Neurosci. Res.* **128**, 1–13 (2018).
28. Kita, Y. *et al.* Cellular-resolution gene expression profiling in the neonatal marmoset brain reveals dynamic species- and region-specific differences. *Proc. Natl. Acad. Sci. U. S. A.* **118**, (2021).
29. Tong, C. *et al.* Multimodal analysis demonstrating the shaping of functional gradients in the marmoset brain. *Nat. Commun.* **13**, 6584 (2022).
30. Woodward, A. *et al.* The Brain/MINDS 3D digital marmoset brain atlas. *Sci. Data* **5**, 180009 (2018).
31. Simpson, G. G. Mammals and the nature of continents. *American Journal of Science* **241**, (1943).
