# Supplementary material for: The cellular correlates and adolescent reorganisation of cortical myelination networks in the common marmoset
Source: Commun Biol. 2026 May 19;9:1066. doi: 10.1038/s42003-026-10276-y (PMC13454510; doi:10.1038/s42003-026-10276-y)
Supplement: Supplementary file 2 [file 42003_2026_10276_MOESM2_ESM.pdf]

Description of Additional Supplementary Files

File name: Supplementary Data 1

Description: full list of cortical areas used in our analyses
