# Supplementary material for: The cellular correlates and adolescent reorganisation of cortical myelination networks in the common marmoset
Source: Commun Biol. 2026 May 19;9:1066. doi: 10.1038/s42003-026-10276-y (PMC13454510; doi:10.1038/s42003-026-10276-y)
Supplement: Supplementary file 3 [file 42003_2026_10276_MOESM4_ESM.pdf]

Corresponding author(s): Ed Hutchings
Last updated by author(s): Oct 14, 2025

Reporting Summary

Nature Portfolio wishes to improve the reproducibility of the work that we publish. This form provides structure for consistency and transparency in reporting. For further information on Nature Portfolio policies, see our Editorial Policies and the Editorial Policy Checklist.

Statistics

For all statistical analyses, confirm that the following items are present in the figure legend, table legend, main text, or Methods section.

n/a	Confirmed	
☐	☒	The exact sample size (*n*) for each experimental group/condition, given as a discrete number and unit of measurement
☐	☒	A statement on whether measurements were taken from distinct samples or whether the same sample was measured repeatedly
☐	☒	The statistical test(s) used AND whether they are one- or two-sided<br>*Only common tests should be described solely by name; describe more complex techniques in the Methods section.*
☐	☒	A description of all covariates tested
☐	☒	A description of any assumptions or corrections, such as tests of normality and adjustment for multiple comparisons
☐	☒	A full description of the statistical parameters including central tendency (e.g. means) or other basic estimates (e.g. regression coefficient) AND variation (e.g. standard deviation) or associated estimates of uncertainty (e.g. confidence intervals)
☐	☒	For null hypothesis testing, the test statistic (e.g. *F*, *t*, *r*) with confidence intervals, effect sizes, degrees of freedom and *P* value noted<br>*Give P values as exact values whenever suitable.*
☒	☐	For Bayesian analysis, information on the choice of priors and Markov chain Monte Carlo settings
☒	☐	For hierarchical and complex designs, identification of the appropriate level for tests and full reporting of outcomes
☐	☒	Estimates of effect sizes (e.g. Cohen's *d*, Pearson's *r*), indicating how they were calculated

Our web collection on statistics for biologists contains articles on many of the points above.

Software and code

Policy information about availability of computer code

Data collection	No software was used for data collection.
Data analysis	Preprocessing of MRI data used Brainsuite18a (Shattuck and Leahy 2002) and Advanced Normalisation Tools v2.5.0 (ANTs; Avants et al. 2011). Preprocessing of in-situ hybridisation data used a modified version of previously published Matlab code (Tong et al. 2022; https://github.com/TrangeTung/marmoset_gradient) and ANTs. Estimation of MIND networks used previously published python code (Sebenius et al. 2023; https://github.com/isebenius/MIND). Downstream analyses were conducted largely in python 3.11 using publicly available packages: Brainsmash (0.11.0), nibabel (5.3.2), numpy (1.26.4), pandas (2.2.3), scanpy (1.9.6), scipy (1.11.3), scikit-learn (1.7.2), pingouin (0.5.5), and statsmodels (0.14.0). Non-linear analyses were conducted in R 4.4.0 using publicly available packages: mgcv (1.9-1), gratia (0.9.0), lme4 (1.1-35.3), and MuMIn (1.48.4). Code and data required to run downstream analyses is available at https://github.com/edhutch1/marm_MIND.

For manuscripts utilizing custom algorithms or software that are central to the research but not yet described in published literature, software must be made available to editors and reviewers. We strongly encourage code deposition in a community repository (e.g. GitHub). See the Nature Portfolio guidelines for submitting code & software for further information.

Data

Policy information about availability of data

All manuscripts must include a data availability statement. This statement should provide the following information, where applicable:

- Accession codes, unique identifiers, or web links for publicly available datasets
- A description of any restrictions on data availability
- For clinical datasets or third party data, please ensure that the statement adheres to our policy

All data analysed in this study are publicly available. Retrograde tract tracing data (Majka et al. 2020) are available from http://marmosetbrain.org. Region-wise hierarchical position assignments (Theodoni et al. 2021) are available from https://doi.org/10.1093/cercor/bhab191. Region-wise cytoarchitectonic class assignments (Atapour et al. 2024) are available from https://www.marmosetbrain.org/whole_brain_cb_maps. MRI data (Hata et al. 2023) are available from the Brain/MINDs 2.0 data portal (https://dataportal.brainminds.jp/marmoset-mri-na216). In-situ hybridisation data (Shimogori et al. 2018 and Kita et al. 2021) are available from the Brain/MINDs Marmoset Gene Atlas data portal (https://gene-atlas.brainminds.jp/). Processed single nucleus RNA-sequencing data (Krienen et al. 2023) are available from the CELLxGENE data repository (RRID:SCR_021059; https://cellxgene.cziscience.com/collections/0fd39ad7-5d2d-41c2-bda0-c55bde614bdb). Marker gene sets used to assign biological identities to interneuron clusters (Bakken et al. 2021) are available from https://doi.org/10.1038/s41586-021-03465-8.

Research involving human participants, their data, or biological material

Policy information about studies with human participants or human data. See also policy information about sex, gender (identity/presentation), and sexual orientation and race, ethnicity and racism.

Reporting on sex and gender	This study did not analyse human data.
Reporting on race, ethnicity, or other socially relevant groupings	This study did not analyse human data.
Population characteristics	This study did not analyse human data.
Recruitment	This study did not analyse human data.
Ethics oversight	This study did not analyse human data.

Note that full information on the approval of the study protocol must also be provided in the manuscript.

Field-specific reporting

Please select the one below that is the best fit for your research. If you are not sure, read the appropriate sections before making your selection.

☒ Life sciences ☐ Behavioural & social sciences ☐ Ecological, evolutionary & environmental sciences

For a reference copy of the document with all sections, see nature.com/documents/nr-reporting-summary-flat.pdf

Life sciences study design

All studies must disclose on these points even when the disclosure is negative.

Sample size	No power analyses were conducted prior to data analysis. The full sample size available to us was N = 446 (N female = 238). After manual and automated quality control, the sample size available was N = 421 (N female = 226). For non-linear analyses, we additionally excluded animals in age ranges with a sampling density below 5% to improve spline estimates, leaving N = 416 animals (N female = 223), aged 0.62-10.67 years. For developmental analyses, we retained animals below 36 months of age, leaving N = 211 (N female = 91). This sample size was sufficiently large to yield stable age predictions when using 5-fold cross-validation: the median mean absolute error (MAE) across 50 folds was approximately 3.5 months when using network edges as predictors. To further demonstrate that this sample size was adequate, we replicated our main results (see Replication) using an independent proxy for myelination—images of myelin basic protein (MBP) expression measured with in situ hybridisation.
Data exclusions	To exclude outliers in MRI-derived phenotypes, we used a combination of manual and automated quality control procedures. First, we visually inspected all T1w/T2w images and manually excluded scans with large artefacts or warping resulting from poor image registration (N = 6 excluded). We also automatically excluded scans falling in the top or bottom 1% of mean T1w/T2w or MIND weighted degree across all regions (N = 20 excluded). Furthermore, because spline-based estimates become unstable in age ranges with sparse data (Wood 2025), we excluded animals falling in age ranges with a sampling density below 5% in non-linear lifespan analyses (Supplementary Figure 11).
Replication	We replicated our main MRI findings using an independent proxy for myelination—images of myelin basic protein (MBP) expression measured using in situ hybridisation. Co-expression of genes in glutamatergic neurons and PV^+ and VIP^+ was most strongly correlated with similarity in both MRI- and MBP-derived maps of myelination (Figure 3). Age-related changes in mean cortical myelination (Figure 4) were highly correlated across modalities. Corresponding similarity networks showed comparable developmental changes (Figure 6), which sensory and association areas clustering together across both modalities, though with some modality-specific differences.

Randomization	No data were collected in this study and no experimental groups were constructed in this study.
Blinding	Blinding was not relevant to our study as we did not perform randomised experiments.

Reporting for specific materials, systems and methods

We require information from authors about some types of materials, experimental systems and methods used in many studies. Here, indicate whether each material, system or method listed is relevant to your study. If you are not sure if a list item applies to your research, read the appropriate section before selecting a response.

Materials & experimental systems

n/a	Involved in the study
☒	☐ Antibodies
☒	☐ Eukaryotic cell lines
☒	☐ Palaeontology and archaeology
☐	☒ Animals and other organisms
☒	☐ Clinical data
☒	☐ Dual use research of concern
☒	☐ Plants

Methods

n/a	Involved in the study
☒	☐ ChIP-seq
☒	☐ Flow cytometry
☐	☒ MRI-based neuroimaging

Animals and other research organisms

Policy information about studies involving animals; ARRIVE guidelines recommended for reporting animal research, and Sex and Gender in Research

Laboratory animals	This study analysed data from common marmosets (Callithrix jacchus). MRI analyses used cross-sectional T1w/T2w ratio data from N = 446 common marmosets spanning 0.62–12.75 years of age (N female = 238; Hata et al., 2023). Retrograde tract-tracing data (Majka et al., 2020) involved 52 adult marmosets (N female = 21, 1.4–4.6 years). Single-nucleus RNA-sequencing data (Krienen et al., 2023) were derived from cortical tissue samples of 6 adult marmosets (29 months - 11 years, 5 months). Each region was sampled in at least one male and one female, except for the posterior cingulate cortex, A23, which was sampled in a single male. In situ hybridisation data (Shimogori et al., 2018; Kita et al., 2021) were obtained from two male marmosets aged 6 months and 4 years.
Wild animals	This study did not analyses data from wild animals.
Reporting on sex	MRI analyses used cross-sectional T1w/T2w ratio data from N = 446 common marmosets (N female = 238; Hata et al., 2023). Sex was included as a covariate in age-related analyses, and an age*sex interaction was tested.
Field-collected samples	This study did not analyses data from field-collected samples
Ethics oversight	Ethical approval was provided by the institutions that collected the original data.

Note that full information on the approval of the study protocol must also be provided in the manuscript.

Plants

Seed stocks	This study did not analyse plant data.
Novel plant genotypes	This study did not analyse plant data.
Authentication	This study did not analyse plant data.

Magnetic resonance imaging

Experimental design

Design type	We used publicly available T1-weighted and T2-weighted images from the Brain/MINDs project (Hata et al. 2023), hence no study design is applicable

Design specifications	Only structural images were used, so design specifications are not applicable.
Behavioral performance measures	No behavioural variables were analysed as part of this study.

Acquisition

Imaging type(s)	Structural (T1-weighted and T2-weighted)
Field strength	9.4T
Sequence & imaging parameters	T1-weighted images were collected using an MP-RAGE sequence (TR = 6000 ms, TE = 2 ms, flip angle = 12°, TI = 1600 ms, voxel size = 0.27 × 0.27 × 0.54 mm, scan time ≈ 20 min). T2-weighted images used a RARE sequence (TR = 4000 ms, TE = 22 ms, RARE factor = 4, flip angle = 90°, voxel size = 0.27 × 0.27 × 0.54 mm, scan time ≈ 7 min 24 s). All images were reconstructed to 0.27mm isotropic voxel resolution. For full details of image acquisition, see Hata et al. (2023).
Area of acquisition	Whole brain

Diffusion MRI ☐ Used ☒ Not used

Preprocessing

Preprocessing software	T2w images were skull-stripped using BrainSuite18a (r = 2, s = 0.6; Shattuck and Leahy 2002) to improve the quality of subsequent image registration. Template registration and atlas label warping were performed using advanced normalisation tools v2.5.0 (ANTs; Avants et al. 2011). An ex vivo T2w template (Woodward et al. 2018) was registered to each subject's T2w image using three sequential transforms: a rigid transform (Rigid[0.1]; mutual information metric, 32 bins; 1000×500×250×100 iterations), an affine transform (Affine[0.1]; mutual information metric, 32 bins; same convergence schedule), and a diffeomorphic SyN transform (SyN[0.1, 3, 0.1]; cross-correlation metric, radius = 4; 100×70×50×2 iterations). All stages used shrink factors of 8×4×2×1 and smoothing sigmas of 3×2×1×0. The resulting warp was applied to the BMA 2019 atlas using antsApplyTransforms with nearest-neighbour interpolation, generating a parcellation label image for each subject in native MRI space. Analyses were conducted in native space to preserve the original resolution and distributional characteristics of voxel-level MRI features.
Normalization	Data were not normalised. Sensitivity analyses revealed that addition of two covariates normally used to normalise T1w/T2w images (median white matter and median vitreous humor signal) did not greatly alter estimates of age-related change in mean T1w/T2w.
Normalization template	We used a publicly available ex-vivo T2-weighted template from the Brain/MINDs project (Woodward et al. 2018).
Noise and artifact removal	Noise was not removed from individual scans. Rather, to address the presence of outliers, we removed scans falling in the top and bottom 1% of mean cortical T1w/T2w or mean weighted degree across all regions. We also visually inspected images and removed scans which contained large, visible artifacts or were warped due to poor registration.
Volume censoring	Our preprocessing pipeline did not use volume censoring.

Statistical modeling & inference

Model type and settings	Modelling and statistical analyses were conducted downstream of MIND network estimation. Analyses included correlation, multiple linear regression to assess age-related changes in regional and network measures, agglomerative hierarchical clustering to identify groups of edges with similar age-related trajectories, and machine-learning to predict age from MRI-derived features. We also conducted supplementary non-linear analyses using general additive models.
Effect(s) tested	Only structural data were considered in this study.

Specify type of analysis: ☐ Whole brain ☒ ROI-based ☐ Both

Anatomical location(s)	ROIs were defined based on the cortical parcellation of Paxinos et al. (2012)
Statistic type for inference (See Eklund et al. 2016)	No voxel-wise analyses were performed. Clustering of voxel-level data was not performed. Clustering of edge-wise age-related changes was performed using agglomerative hierarchical clustering (distance metric = Euclidean distance, linkage metric = Ward linkage). Clustering solutions with N=2, 3, and 4 clusters were analysed in this study.
Correction	Corrections for FDR and FWER were performed in this study, though not for voxel-level analyses.

Models & analysis

n/a	Involved in the study	
☒	☐	Functional and/or effective connectivity
☐	☒	Graph analysis
☐	☒	Multivariate modeling or predictive analysis

Graph analysis	Edges of structural similarity networks were the subject of analyses. Weights were not thresholded. At the node level, weighted degree (average over all non-thresholded edges for that cortical area) was analysed. Analyses were performed both at the individual level and at the group level.
Multivariate modeling and predictive analysis	Multivariate analyses were used to assess age-related changes in regional mean T1w/T2w, regional weighted degree, or similarity network edge weights. All models included age and sex as a independent variables and sensitivity analyses assessed age*sex interactions, the inclusion of normalisation covariates (median white matter signal or median vitreous humor signal) in mean T1w/T2w models, and the inclusion of estimated total intracranial volume in degree models. Agglomerative hierarchical clustering (distance metric = Euclidean distance, linkage metric = Ward linkage) was used to reduce the dimensionality of the resulting matrix of edge level changes.<br><br>To assess the extent to which MIND networks capture developmental changes in myelination, we trained machine learning models to predict age from T1w/T2w scans in N=211 animals aged 7 months (pre-pubertal) to 36 months (mature adult). Repeated 5-fold cross validation with 50 random permutations of the data was used to compare age prediction performance for models trained using either i) mean T1w/T2w per cortical area, ii) MIND weighted degree per cortical area, or iii) MIND edges between each pair of cortical areas. Each fold was balanced for sex and subjects in the top or bottom 1% of mean T1w/T2w or MIND weighted degree (averaged over all cortical areas) were excluded to reduce the impact of outliers. For each predictor, we trained and tested models on 50 random splits of the data (20% of subjects, N=43, retained in each test fold), stratified by sex. Within each outer training fold, 5-fold cross validation, again stratified by sex, was used to select between several models. For regional predictors (mean T1w/T2w and weighted degree), we used: a support vector regression with a radial basis function kernel and C regularisation values of [0.1, 1, 10, or 100]; and a gaussian process regression (GPR) with a summed linear and white noise kernel and alpha value of 1×10-10. We did not use other values of alpha in our final GPR models to reduce computation time and because preliminary tests demonstrated that modulating alpha to [10-5, 10-3, or 10-2] did not change model predictions. The model with the minimum mean absolute error (MAE) across 5 internal validation folds was trained on the entire training set for that outer fold. At the edge level, due to the much larger feature space (26335 edges) we used only the GPR model (Sebenius et al. 2023). For each outer test fold, models were evaluated using the mean absolute error (MAE) and the correlation between actual and predicted age values. We used partial correlations to control for the effects of sex and eTIV. Regressing variables out of the feature space can lead to data leakage as estimating effects from the entire dataset allows information from the test set to indirectly influence the training process, inflating model performance (Dinga et al. 2020). We therefore opted to use partial correlations as a post-hoc adjustment (Sebenius et al. 2023).
