# Supplementary material for: The cellular correlates and adolescent reorganisation of cortical myelination networks in the common marmoset
Source: Commun Biol. 2026 May 19;9:1066. doi: 10.1038/s42003-026-10276-y (PMC13454510; doi:10.1038/s42003-026-10276-y)
Supplement: Supplementary file 4 [file 42003_2026_10276_MOESM5_ESM.pdf]

nature portfolio

Peer Review File

The cellular correlates and adolescent reorganisation of cortical myelination networks in the common marmoset

Corresponding Author: Mr Ed Hutchings

This file contains all reviewer reports in order by version, followed by all author rebuttals in order by version.

Version 0:

Reviewer comments:

Reviewer #1

(Remarks to the Author)
Overall, this work is incredibly ambitious and technically interesting. I wish authors would add more explanation to their results so it is easier to readers to follow. For example, in Figure 5, there is no explanation on how the networks were calculated (tracer-based? Diffusion?) or why these network metrics were used. Figure 3 shows a higher correlation for Glut, why not investigate that cell type instead of inhibitory neurons? Even more, why not look into oligos if the correlation was with a myelin map? This is an exciting and meaningful work, if authors make further improvements in the text to make it more reader friendly, it will undoubtedly have a broad impact on the neuroscientific field.

Introduction
Diffusion tissue properties and tractography are different modalities. The author should compare myelin maps with tissue properties and not tractography itself.

Methods
Authors should add the basic MRI acquisition data to the paper such as resolution, TR and TE. If animals were registered to a higher resolution template, could that interpolation affect the following analyses as it would artificially increase the voxel count?

Fig 1. Are these analyses Age and sex corrected? Do you see sex differences? MIND seems to be using the distribution of each area to do its calculations, but if the image was upsampled upon registration, it might feed more voxels and therefore inflate the statistical power?

Fig. 2 Why average 340 animals? Since it compares with an atlas, wouldn't it be more useful to select animals with similar age/sex to the ones that were used for the atlas?
Alternatively, are these data age and sex corrected?
Why not use MIND to directly compare T1w/T2w with MBP expression instead of a correlation?

Fig 3 why oligos don't correlate to MIND?

"When we repeated this analysis substituting MBP MIND 317 edge weights for T1w/T2w MIND edge weights, we found much stronger relationships 318 with cell-type specific genome co-expression. Inter-areal co-expression by 319 glutamatergic neurons was most strongly correlated with MBP MIND"
Would that be the other way around? 3C shows MBP results

Fig4. C , "though this trend did not 373 remain significant when accounting for spatial autocorrelation." The authors should include in the main figure the corrected p-values.

Fig 4D. Are both T1/T2 and MBP using only 2 animals of 6 months and 2 years? The disparities in sample sizes, sex, and age ranges could influence the analyses.

Fig 5. I was not able to comprehend this result. What is the mean network in line 403 and 404? Without understanding this, I was also unable to understand Fig 5B. Were the features used in isolation? Or is there a model that combine the 3 features

with a better age estimate in C?

Reviewer #2

(Remarks to the Author)
This is an extremely rigorous and valuable study that successfully integrates macroscale neuroimaging (T1w/T2w MIND network) with molecular and cellular transcriptomic data (MBP mRNA, snRNA-seq) to characterize the conserved patterns of myeloarchitectural maturation in the common marmoset. The work successfully establishes the biological validity of the MRI-derived similarity network and identifies a sensory-association gradient of adolescent reorganization that mirrors findings in humans, strongly supporting the marmoset's utility as a translational model.
The paper is likely to significantly influence thinking in the field by demonstrating the utility of MIND networks in capturing cellular-level biological reality and by consolidating the marmoset as a key model for neurodevelopmental studies.While the work is robust, addressing the following points would further strengthen the conclusions:

1. Biological Interpretation of MBP MIND Superiority
The results show that MBP MIND similarity is substantially more strongly correlated with snRNA-seq gene co-expression (e.g., Glutamatergic r=0.67) than T1w/T2w MIND(r = 0.44), nominally significant only).
Please elaborate in the Discussion on the reasons for this marked difference. Does MBP MIND (derived from mRNA density) act as a near-gold standard for pure cellular myeloarchitecture similarity, thereby demonstrating the biological ceiling for correlation with gene expression, while T1w/T2w MIND is constrained by sensitivity to non-myelin factors (e.g., iron, water) or image noise? A detailed discussion here would be important.

2. Developmental Modeling and Non-Linearity
The MIND network correlation analysis (Figure 5A) and age prediction (Figure 5C) suggest a plateau in developmental change beginning around 18-21months.
The primary developmental analysis uses multiple linear regression across the 7-36 months range. Given the suggested non-linearity, please justify the choice of a linear model, or confirm that sensitivity analyses using a non-linear model (e.g., piecewise linear or quadratic term) were performed to ensure that the reported linear Bage coefficients are the most appropriate representation of the adolescent reorganization trajectory.

3. Interpretation of Auditory Cortex Differentiation
The finding that the Auditory Cortex (AUD) shows a significant decrease in nodal degree (myeloarchitectonic differentiation) during adolescence is compelling. While this may suggest functional specialization related to vocal communication, the observed structural differentiation should also be interpreted in the context of the AUD's high susceptibility to experience-dependent plasticity throughout life, particularly in the common marmoset.
-Current literature suggests that the marmoset auditory cortex:
Undergoes significant experience-dependent reorganization based on long-term exposure to self-generated vocalizations (Chuang et al., 2005).
-Exhibits a high degree of vocal plasticity during development, which is tied to social interaction and environmental cues (Gultekin et al., 2021).
-Displays dynamic, context-dependent modulation (facilitation/suppression) of neural responses based on the immediate sound environment (Wang et al., 2025).

Please discuss the AUD's structural differentiation as a potential reflection of its enhanced sensitivity and vulnerability to environmental/social influences. The high plasticity in AUD may lead to increased inter-individual variability in its myelin distribution pattern, causing it to become less similar to the average cortical pattern and thus resulting in the observed decrease in MIND Degree (structural divergence). This interpretation directly complements the Discussion's existing argument regarding the enhanced adult plasticity in higher-order areas leading to increased inter-individual variability.

4. Reproducibility concerns
Please clarify the definition of the cortical network nodes. Specifically, were the cortical regions defined bilaterally (e.g., Left V1 and Right V1 as separate nodes), or was the analysis restricted to a single hemisphere? This is vital for interpreting network topology.

5. Figure
There was no description about Figure 3E. The description about 3E seemed to be included in 3D.

Reviewer #3

(Remarks to the Author)
Summary and General Assessment:
This paper investigates MRI-derived structural similarity in the common marmoset brain using Kullback–Leibler (KL) divergence and examines its correspondence with multiple forms of biological data—including gene expression patterns, single-cell transcriptomic profiles, and tracer-based cytoarchitectural information—sourced from publicly available databases. The approach is promising for advancing our understanding of primate architectomics, with potential relevance to human brain research, and it highlights the utility of large-scale, data-driven methodologies. However, the clarity of the manuscript limits its overall impact. Substantial revision of the narrative structure and presentation would improve readability

and strengthen the contributions of the study.
Major Comments:
1. Lines 211–215 and Figure 1C: Is there a reason the manuscript does not report a p-value for the correlation between Euclidean distance and MINDS similarity, while significance is reported for the correlations with T1w/T2w similarity and axonal connectivity? Does this omission imply that the association was not significant? Please clarify and, if applicable, provide the corresponding statistical results.
2. It would be more appropriate to compare age-matched measures—specifically, the T1w/T2w averages for 4-year-old animals and the MBP map derived from 4-year-old data rather than averaging across all ages. Using age-specific values would yield a more accurate interpretation of the relationship between myelin proxies and MBP expressions.
3. Please clarify why the analysis of age-related changes was restricted to developmental and young-adult ages, rather than including the full dataset, which contains older individuals (>8 years). The rationale for defining the developmental period as up to 36 months is unclear. Prior literature typically defines sexual maturity around 18–24 months, and the MBP datasets used by the authors involve animals at 0.6 and 4 years of age. If the assumption is that T1w/T2w values plateau after approximately 3 years of age, a nonlinear regression analysis would be more appropriate to test this hypothesis. If the intention is to focus specifically on developmental processes, it may be clearer to frame the analysis as "age-related developmental change" rather than simply "age-related change."
Minor Comments:
1. In addition to the MIND similarity heatmap, providing region-wise KL divergence maps—particularly for comparisons between primary sensory and prefrontal association cortical zones, where the authors report strong dissimilarity—would greatly enhance and clarify the interpretability of the results.
2. Please include the term "cell type–specific correlated gene expression (CGE)" in the legend of Figure 3C, as it does not appear in the manuscript until line 866.
Technical Comments:
1. Lines 708–710: I guess the T1w image matrix in native space does not match that of the T2w image (T1w is acquired in 3D, whereas T2w is acquired in 2D). Consequently, the voxel positions in T1w do not correspond to those in T2w. How, then, was the T1w/T2w ratio computed without performing co-registration? Clarification on this procedure is needed.
2. The ISH volume appears to be missing occipital areas, as noted in Lines 828–830. How was the information from "at least one representative tissue sample analysable" registered and transformed into MRI space under these circumstances? The lack of occipital coverage raises concerns regarding the validity of the MINDS-derived MBP estimates in those regions.
Typographical and Grammatical Errors:
1. The font size in several sections (e.g., Lines 208–223 and the legend of Supplementary Figure 6) appears smaller than the surrounding text. Please ensure consistent formatting throughout the manuscript.
2. Line 314: Although not a typographical error, it would be clearer to specify "T1w/T2w MIND" rather than simply "MIND," to distinguish it from the MBP-derived MIND measure.
3. Line 334: "Figure 3B–C" appears to refer instead to Figures 3D–E. Line 338: "Figure 3D" may also need to be updated to "Figure 3D–E." Please verify and correct the figure references accordingly.

Version 1:

Reviewer comments:

Reviewer #1

(Remarks to the Author)
I thank the authors for answering all my questions and concerns and for their hard work on this manuscript. I believe the manuscript will be of interest to the vast readership of Commsbio and conveys an important scientific approach on studying myeloarchitectural development on marmosets

Reviewer #2

(Remarks to the Author)
I would like to thank the authors for their thorough and thoughtful response to my previous comments.The manuscript presents a compelling, multi-scale integration of neuroimaging and transcriptomics that will be of great interest to the neurodevelopmental and primate research communities.

Reviewer #3

(Remarks to the Author)
The authors have adequately addressed all of my previous comments in this second round of review. The manuscript has been substantially improved overall and is now suitable for publication. While the amount of supplementary material is somewhat extensive, I understand that this may be necessary. I have no further comments.

Open Access This Peer Review File is licensed under a Creative Commons Attribution 4.0 International License, which permits use, sharing, adaptation, distribution and reproduction in any medium or format, as long as you give appropriate credit to the original author(s) and the source, provide a link to the Creative Commons license, and indicate if changes were made.
In cases where reviewers are anonymous, credit should be given to 'Anonymous Referee' and the source.
The images or other third party material in this Peer Review File are included in the article's Creative Commons license, unless indicated otherwise in a credit line to the material. If material is not included in the article's Creative Commons license and your intended use is not permitted by statutory regulation or exceeds the permitted use, you will need to obtain permission directly from the copyright holder.
To view a copy of this license, visit https://creativecommons.org/licenses/by/4.0/

Response to reviewers: "The cellular correlates and adolescent reorganisation of cortical myelination networks in the common marmoset"

We thank the editor and reviewers for their insightful comments. Below are point-by-point responses to reviewer comments, which include: a quote of the reviewer's comment (in blue); our response to the comment (in black); and all relevant changes made to the main or supplementary text (in red).

Reviewer 1

1.1

"Overall, this work is incredibly ambitious and technically interesting. I wish authors would add more explanation to their results so it is easier to readers to follow."

We thank the reviewer for their positive feedback. We have added substantial explanatory material to support readers' understanding of our results, with multiple specific changes (detailed below) made throughout the paper to enhance overall accessibility of the paper.

1.2

"For example, in Figure 5, there is no explanation on how the networks were calculated (tracer-based? Diffusion?) or why these network metrics were used."

We apologise for any lack of clarity in the text accompanying **Figure 5**. **Figure 5** represents the same T1w/T2w MIND similarity networks that were estimated and validated against anatomical and transcriptomic benchmark data in the earlier part of the study (**Figure 1-3**). We have changed the manuscript in the **Results** section to explain in more detail why we use these metrics:

"Models were trained to predict age from an individual scan using three distinct sets of features: i) regional mean T1w/T2w signal itself; ii) MIND networks summarised by the weighted degree of each node; and iii) all edge weights in the MIND network. We reasoned that if models trained using similarity network metrics (ii and iii) provided more accurate age predictions than a non-network metric (i), this would suggest that similarity networks are more sensitive to the subtle biological changes occurring during adolescent brain development, prioritising their adoption in future neurodevelopmental MRI studies."

1.3

"Figure 3 shows a higher correlation for Glut, why not investigate that cell type instead of inhibitory neurons?"

We thank the reviewer for sharing this question. We outline our reasoning for not investigating excitatory neuron subtype correlations in the **Supplementary Methods** and discuss this as a limitation in the **Discussion**.

Updated **Supplementary Methods**:

"

Cell type-specific correlated gene expression network analysis

Analysis of glutamatergic neuronal subtypes

We elected to not analyse the relationships between myeloarchitectonic similarity and glutamatergic subtype-specific correlated gene expression for two reasons: i) the cell subtype marker gene sets we used are less reliable for excitatory glutamatergic neurons, complicating the biological interpretation of subtypes of glutamatergic neurons; and ii) conducting statistical tests of association between multiple glutamatergic neuronal subtypes and MIND phenotypes would increase the severity of the adjusted false positive threshold for multiple comparisons with commensurate loss of statistical power.

Firstly, to assign cell type clusters to a biological identity, we computed the mean Z-scored expression of cell type marker gene sets obtained from the marmoset primary motor cortex (Bakken et al. 2021, **Supplementary Figure 10**). Given that primary motor cortex lacks a granular cell layer and contains glutamatergic neuronal subtypes with unique morphologies (e.g. Betz cells), we expected that the glutamatergic marker gene sets may not generalise to other cortical areas.

Secondly, though glutamatergic neurons show transcriptomic diversity across the cortex (Krienen et al. 2023), we observed that glutamatergic subtype-specific correlated gene expression tended to be more highly collinear (mean pairwise r = 0.75), than interneurons (mean pairwise r = 0.61). We reasoned that these glutamatergic subtypes would be similarly associated with MIND phenotypes and their inclusion in the full analysis would worsen the multiple comparisons problem and reduce power to detect associations between myeloarchitectonic similarity and interneuronal gene co-expression."

Updated **Discussion**:

"Second, it would in principle be interesting to analyse the relationships between MIND MRI phenotypes and excitatory neuronal subtypes. Previous work has shown that excitatory subtypes differ in their association with mean cortical myelin across the cortex (Burt et al. 2018; Fulcher et al. 2019). Moreover, activity-dependent myelination is influenced by factors such as growth factor signalling and neuronal diameter (Mount and Monje 2017), both of which vary across glutamatergic subtypes. However, for

methodological reasons detailed in Supplementary Information, here we restricted our focus to inhibitory inter-neuronal subtypes."

1.4

"Even more, why not look into oligos if the correlation was with a myelin map?
This is an exciting and meaningful work, if authors make further improvements in the text to make it more reader friendly, it will undoubtedly have a broad impact on the neuroscientific field."

We thank the reviewer for this important comment. We report correlations between MIND MRI similarity and gene expression in two major oligodendrocyte subtypes in the **Supplementary Information**, and reference this section in the relevant **Results** section.

Updated **Supplementary Information**:

"

Analysis of oligodendrocyte subtypes

Given that the principal phenotype of interest in our study is myelination, we elected to additionally investigate the relationships between oligodendrocyte subtype gene co-expression and MIND phenotypes. Oligodendrocyte cluster 1 mapped onto mature oligodendrocytes (ODCs) while cluster 2 mapped onto oligodendrocyte precursor cells (OPCs). Cluster 3 included only 0.05% of total oligodendrocytes, so was excluded. We repeated the correlation analysis in **Figure 3**, this time using these oligodendrocyte subtypes instead of interneuron subtypes.

ODC gene co-expression was correlated with T1w/T2w MIND ($r = 0.10$, $p = 0.531$) and with *MBP* MIND ($r = 0.12$, $p = 0.340$); while OPC gene co-expression was more strongly correlated with T1w/T2w MIND ($r = 0.17$, $p = 0.486$) and with *MBP* MIND ($r = 0.27$, $p = 0.144$). However, none of these correlations were significant when accounting for spatial autocorrelation. Although provisional, this pattern of results is biologically plausible. OPCs proliferate and differentiate in response to neuronal activity (Barres and Raff 1993; Emery 2010; Mount and Monje 2017), and therefore undergo transcriptional changes in response to activity in local neuronal circuits. Whereas, ODCs are terminally differentiated, so reflect a more transcriptionally stable cellular state. This may result in enhanced transcriptional coupling between OPC gene expression and myeloarchitecture relative to mature ODCs. Definitive testing of this hypothesis remains a focus for future studies which are sufficiently powered."

Updated **Results**:

"Furthermore, we observed non-significant associations between T1w/T2w and *MBP* MIND and gene co-expression in two oligodendrocyte subtypes (**Supplementary Methods**)."

1.5

"Introduction
Diffusion tissue properties and tractography are different modalities. The author should compare myelin maps with tissue properties and not tractography itself."

We agree that diffusion tissue properties and tractography are different modalities. In this study, MRI-derived myelin maps have been compared only to tissue properties, for instance, by spatial correlation with fine-grained cortical maps of myelin basic protein (*MBP*). However, many different modalities of network data can be compared topologically, in terms of weighted connections or associations between nodes, allowing us to compare the brain network represented by MIND to the "gold-standard" connectome represented by tract-tracing data. We have clarified this point in the **Methods**:

"This pipeline allowed us to estimate the distribution of *MBP* mRNA expression in each cortical area, with comparable spatial resolution to the T1w/T2w images (isotropic voxel resolution of *MBP* mRNA images = 0.10mm; T1w/T2w images = 0.27mm). The resulting cortical maps were subsequently used to estimate MIND networks representing inter-areal similarity of *MBP* expression on the same spatial scale and using the same areal parcellation as the MIND networks derived from T1w/T2w images. Following this procedure, we had tissue-level maps of *MBP* mRNA, which could be directly associated with MRI-derived T1w/T2w maps, and networks of *MBP* expression similarity, which could be topologically associated with networks of T1w/T2w similarity."

1.6

"Methods
Authors should add the basic MRI acquisition data to the paper such as resolution, TR and TE."

We thank the reviewer for their comment. The main text has been updated accordingly in the **Methods**:

"We used T1-weighted (T1w) and T2-weighted (T2w) images acquired at 9.4T from the Brain/MINDS project, currently the world's largest open-access marmoset MRI data resource (Hata et al. 2023). The dataset contains cross-sectional MRI images from N = 446 marmosets, spanning 0.62 to 12.75 years (N female = 238). T1w images were collected using a magnetisation-prepared rapid gradient echo (MP-RAGE) with repetition time (TR) = 6000 ms, echo time (TE) = 2 ms, flip angle = 12°, number of averages (NA) = 1, inversion time = 1600 ms, and scan time = 20 min. T2w images were collected using rapid acquisition with relaxation enhancement (RARE) with TR = 4000 ms, TE = 22 ms, RARE factor = 4, flip angle = 90°, NA = 1, and scan time = 7 min, 24 s. Reconstructed T1w and T2w images had identical matrix dimensions (178 × 178 × 96), voxel sizes (0.27 mm isotropic), orientations (RAS), and voxel-to-RAS affine transformation matrices, indicating a one-to-one correspondence of voxel coordinates across modalities. For full details on image acquisition, see Hata et al. 2023."

1.7

"If animals were registered to a higher resolution template, could that interpolation affect the following analyses as it would artificially increase the voxel count?"

We thank the reviewer for raising this query. We agree that image interpolation can influence the results of MIND MRI analysis by effects on the number of (independent) voxels or vertices available for estimation of the Kullback-Liebler divergence (Sebenius et al. 2023). However, this is not a material concern in this study because all images were analysed in their native space without interpolation or template registration.

We have emphasised in **Methods** that MRI images were analysed in native space:

"An ex-vivo T2w template (Woodward et al. 2018) was warped to each T2w image, after which the warp was applied to the BMA 2019 atlas to generate a label image for each subject in native MRI space. Animals were analysed in native space to preserve the original resolution and distributional characteristics of voxel-level MRI features."

1.8

"Fig 1. Are these analyses Age and sex corrected?"

We thank the reviewer for this comment. We repeated the analysis in **Figure 1D** using age and sex matched animals. The resulting correlation and p-value were unchanged. The remaining analyses in **Figure 1** compared the mean adult T1w/T2w similarity network with population-level reference datasets (i.e., cortical zone assignments, Euclidean distance matrix, and cytoarchitectonic class labels). We expect that these reference measures do not vary by sex or adult age, age and sex correction was not applicable for these comparisons.

1.9

"Do you see sex differences? "

Sex was included as a covariate in all linear models of mean T1w/T2w, regional MIND degree, and MIND edge weights, but did not reach significance in any ROI or edge after multiple comparisons correction. This has been reflected in the **Methods**:

"We included sex as a covariate in all models as studies have demonstrated sex differences in regional cortical mean T1w/T2w in humans (Küchenhoff et al. 2024). However, the main effect of sex was not significant in any model after correction for multiple comparisons."

1.10

"MIND seems to be using the distribution of each area to do its calculations, but if the image was upsampled upon registration, it might feed more voxels and therefore inflate the statistical power?"

We thank the reviewer for this comment. As noted in response to **1.7** the MRI data were analysed in their native space, therefore there was no upsampling due to template co-registration.

1.11

"Fig. 2 Why average 340 animals? Since it compares with an atlas, wouldn't it be more useful to select animals with similar age/sex to the ones that were used for the atlas Alternatively, are these data age and sex corrected?"

We thank the reviewer for this excellent suggestion, which we have now implemented. Age- and sex-matching animals marginally increased the magnitude and decreased the p-values of correlations in **Figure 3**. We make reference to this matching procedure and report updated correlations and p-values for the age- and sex-matched analyses in the manuscript:

"The resulting *MBP* mRNA image volume was compared with a mean T1w/T2w volume formed by averaging T1w/T2w images from N = 16 age- and sex-matched animals… Regionally parcellated cortical maps of mean *MBP* expression and mean T1w/T2w (**Figure 2B**) were positively correlated (Spearman r = 0.73, pvariogram < 0.001, **Figure 2C**). Standard deviation and skewness, quantifying the shape of each regional distribution, were less strongly, though still significantly, correlated between *MBP* mRNA and T1w/T2w MRI images (**Supplementary Figure 8**).

Given the high correspondence between T1w/T2w and *MBP* expression, we further hypothesised that *MBP* MIND networks would show a highly similar organisation to T1w/T2w MIND networks. Since each cortical area in the *MBP* mRNA image contains a distribution of voxel values of *MBP* expression, we could estimate the *MBP* MIND network using identical methods and a commensurate cortical parcellation as was used to estimate T1w/T2w MIND networks (**Figure 2D**). Across all 26,335 edges, T1w/T2w and *MBP* similarity were significantly correlated (Spearman r = 0.42, pvariogram < 0.001), and the strength of correlation was weaker (non-significantly) for regions highest in the cortical hierarchy (Spearman r = -0.38, pvariogram = 0.208, **Figure 2E**)."

1.12

"Why not use MIND to directly compare T1w/T2w with MBP expression instead of a correlation?"

We thank the reviewer for this interesting suggestion. The proposed analysis is answering a slightly different question to our original analysis in **Figure 2E**, therefore results are not convergent. We have added this analysis to the **Supplementary Information**:

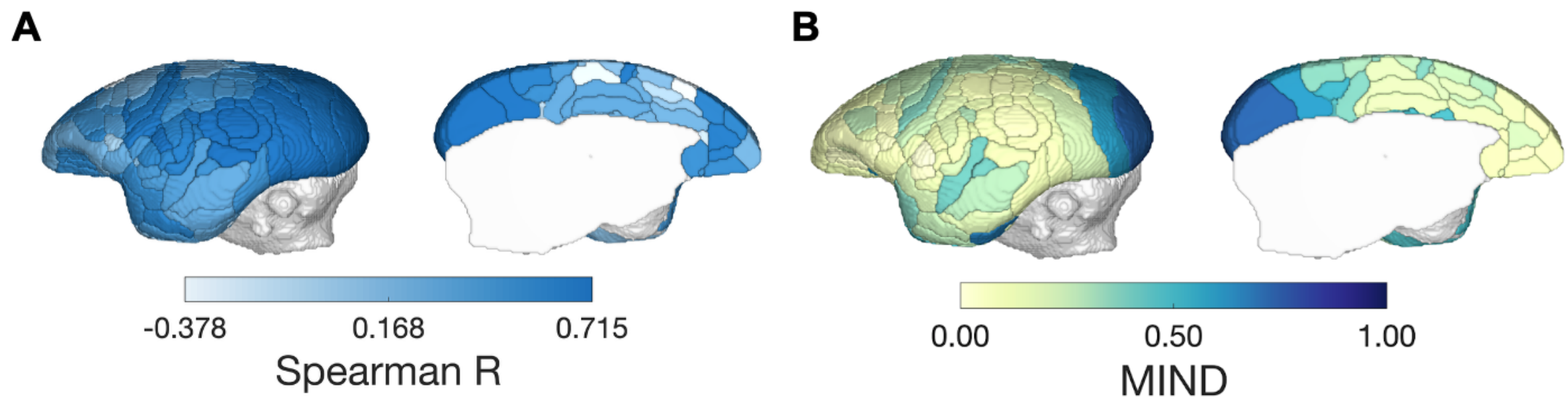

***"Supplementary Figure 9: Cross-modal concordance of MIND edges and cross-modal similarity of signal intensity distributions. A** Map showing correlation between each cortical region's T1w/T2w MIND edges and MBP MIND edges (**Figure 2E**). **B** Map showing MIND similarity values directly computed between the T1w/T2w ratio and MBP expression signal intensity distributions for each cortical region in the left hemisphere. Resulting maps were poorly correlated (r = -0.17, p = 0.32), indicating that region-wise cross-modal concordance in MIND edge weights reflects not only the distribution properties of those regions but their relationships to all other cortical areas."*

We also reference this analysis in the updated the **Results** section:

"Across all 26,335 edges, T1w/T2w and *MBP* similarity were significantly correlated (Spearman r = 0.42, pvariogram < 0.001), and the strength of correlation was weaker (non-significantly) for regions highest in the cortical hierarchy (Spearman r = -0.38, pvariogram = 0.208, **Figure 2E**). This was not driven exclusively by cross-modal similarity in signal intensity distributions, which displayed a distinct cortical pattern (**Supplementary Figure 9**)."

1.13

"Fig 3 why oligos don't correlate to MIND?"

We thank the reviewer for calling attention to this somewhat counter-intuitive finding. We suspect that this is due to insufficient statistical power, and have highlighted this in the limitations section of the **Discussion**:

"Furthermore, we did not observe significant correlations between myeloarchitectonic similarity and oligodendrocyte gene co-expression, which we expected given that oligodendrocytes are the key cell type responsible for myelination. We suspect that this is due to insufficient statistical power: expanding the number of cortical areas for

which single cell RNA sequencing data are available (beyond the 12 areas openly provided by Krienen et al. (2023), which allow estimation of 66 pair-wise inter-areal similarities or edges) would allow for a finer-grained and more comprehensive comparison between cortical networks of structural similarity and gene co-expression."

1.14

" "When we repeated this analysis substituting MBP MIND 317 edge weights for T1w/T2w MIND edge weights, we found much stronger relationships 318 with cell-type specific genome co-expression. Inter-areal co-expression by 319 glutamatergic neurons was most strongly correlated with MBP MIND". Would that be the other way around? 3C shows MBP results "

We thank the reviewer for bringing our attention to this lack of clarity, which we have addressed in the updated manuscript:

"When we repeated this analysis using *MBP* MIND edge weights instead of T1w/T2w MIND edge weights, we found much stronger relationships with cell-type specific genome co-expression"

1.15

"Fig4. C , "though this trend did not 373 remain significant when accounting for spatial autocorrelation." The authors should include in the main figure the corrected p-values."

The reviewer is referring to a supplementary analysis (**Supplementary Figure 12A** in the updated manuscript). The corrected p-values are reported in this figure legend rather than in the main text.

1.16

"Fig 4D. Are both T1/T2 and MBP using only 2 animals of 6 months and 2 years? The disparities in sample sizes, sex, and age ranges could influence the analyses."

We thank the reviewer for raising this important point. In **Figure 4D**, rates of change in regional mean T1w/T2w were estimated from N = 211 animals using multiple linear regressions, while rates of change in regional mean *MBP* expression were estimated as the age-normalised difference between N = 2 animals. Our use of N = 2 animals to estimate adolescent change in *MBP* expression was a necessary simplification given our limited data access, and we accept that the disparity in sample sizes between modalities could influence our analyses. However, we expect the magnitude of this influence to be small, given that previous sensitivity analyses in section **1.11** revealed that correlations and p-values were highly stable when using a smaller number (N = 16) of age- and sex-matched animals, instead of all animals, to estimate T1w/T2w images in adult-based analyses.

1.17

"Fig 5. I was not able to comprehend this result. What is the mean network in line 403 and 404? Without understanding this, I was also unable to understand Fig 5B. Were the features used in isolation? Or is there a model that combine the 3 features with a better age estimate in C?"

We apologise for any lack of clarity in the text accompanying **Figure 5**, and have updated the manuscript to enhance clarity. In **Figure 5A**, the "mean network" for an age bin refers to the average over all edges for subjects in that corresponding age range. In **Figure 5B,** features were used in isolation to predict age, meaning there were three separate models predicting age using either region-wise mean T1w/T2w, region-wise MIND weighted degree, or edge-wise MIND similarity. We have updated the text accompanying **Figure 5** to enhance clarity:

"We hypothesised that T1w/T2w MIND would also show age-related changes representative of the inter-areally coordinated maturation of cortical myeloarchitecture. To qualitatively assess whether T1w/T2w MIND networks showed age-related changes, we assigned individual networks for animals aged <3 years to one of 7 age-defined bins (each spanning ≥4 months)**,** estimated the mean network for each age bin by averaging across edges for animals within that bin, and computed the correlations between all pairs of averaged networks (**Figure 5A**).
…
To assess the extent to which T1w/T2w MIND networks capture developmental changes which are chronologically consistent across individuals, we used machine learning models to predict subject age. Models were trained to predict age from an individual scan using one of three distinct sets of features: i) regional mean T1w/T2w signal itself; ii) MIND networks summarised by the weighted degree of each node; and iii) all edge weights in the MIND network."

Reviewer 2

2.1

"This is an extremely rigorous and valuable study that successfully integrates macroscale neuroimaging (T1w/T2w MIND network) with molecular and cellular transcriptomic data (MBP mRNA, snRNA-seq) to characterize the conserved patterns of myeloarchitectural maturation in the common marmoset. The work successfully establishes the biological validity of the MRI-derived similarity network and identifies a sensory-association gradient of adolescent reorganization that mirrors findings in humans, strongly supporting the marmoset's utility as a translational model.
The paper is likely to significantly influence thinking in the field by demonstrating the utility of MIND networks in capturing cellular-level biological reality and by consolidating the marmoset as a key model for neurodevelopmental studies.While the work is robust, addressing the following points would further strengthen the conclusions:"

We thank the reviewer deeply for their detailed and positive comments.

2.2

"1. Biological Interpretation of MBP MIND Superiority
The results show that MBP MIND similarity is substantially more strongly correlated with snRNA-seq gene co-expression (e.g., Glutamatergic r=0.67) than T1w/T2w MIND(r = 0.44), nominally significant only). Please elaborate in the Discussion on the reasons for this marked difference. Does MBP MIND (derived from mRNA density) act as a near-gold standard for pure cellular myeloarchitecture similarity, thereby demonstrating the biological ceiling for correlation with gene expression, while T1w/T2w MIND is constrained by sensitivity to non-myelin factors (e.g., iron, water) or image noise? A detailed discussion here would be important."

We thank the reviewer for highlighting this important discussion point and agree completely with their suggested interpretation. We have further highlighted this limitation in the updated **Discussion**:

"... non-myelin and image acquisition-related factors, through influencing regional T1w/T2w distribution shapes and therefore the inter-areal similarity of regional T1w/T2w distributions, constitute a source of non-myeloarchitectonic variability in T1w/T2w similarity networks, which could explain why we observed only nominally significant correlations between T1w/T2w MIND and gene co-expression. The absence of these non-myelin factors in *MBP* images means that these images more faithfully represent cortical myelination patterns and provide a ceiling for correlations between myeloarchitectonic similarity and gene co-expression."

2.3

"2. Developmental Modeling and Non-Linearity
The MIND network correlation analysis (Figure 5A) and age prediction (Figure 5C) suggest a plateau in developmental change beginning around 18-21months. The primary developmental analysis uses multiple linear regression across the 7-36

months range. Given the suggested non-linearity, please justify the choice of a linear model, or confirm that sensitivity analyses using a non-linear model (e.g., piecewise linear or quadratic term) were performed to ensure that the reported linear Bage coefficients are the most appropriate representation of the adolescent reorganization trajectory."

We thank the reviewer for raising this important consideration. We have undertaken novel non-linear analyses of age-related changes in regional mean T1w/T2w, regional MIND degree, and MIND edge weights, which we have added to the **Supplementary Information**. We also provide clarification for our use of linear models in downstream analyses as a simplification to facilitate comparisons with *MBP* expression data in **Results**, and acknowledge this as a limitation of our study in the **Discussion**.

Updated **Supplementary Information**:

Non-linear modelling

Previous literature in humans has suggested that myelination continues to increase beyond the onset of adulthood, into the 30s or 50s (Grydeland et al. 2019). To assess whether marmosets display protracted increases in myelination beyond the accepted onset of sexual maturity at 18-24 months (Sawiak et al. 2018), we fit non-linear models to regional mean T1w/T2w, regional MIND weighted degree, and MIND edge weights across the lifespan to identify timepoints of significant change. We used generalized additive models (GAMs) to flexibly capture developmental trajectories without imposing a predefined polynomial form, implemented using the *mgcv* package in R (Wood 2025). Because spline-based estimates become unstable in age ranges with sparse data (Wood 2025), we excluded ages with a sampling density below 5%, corresponding to subjects older than 10.6 years.

To determine whether developmental effects were better characterised as linear or non-linear, we fit the following model:

$$Y \sim \text{Age} + s(\text{Age},\, k = 10,\, m = c(2,0)) + \text{Sex} \qquad (3)$$

Specifying m = c(2,0) removes the constant and linear components of the smooth term, such that it captures only non-linear changes. Linear and non-linear age effects are therefore estimated simultaneously but remain statistically separable. A significant smooth term indicates that the trajectory deviates significantly from a straight line, reflecting the presence of non-linear effects beyond the linear age term (Wood 2017). For features with a significant smooth term, we fit a full GAM and extracted ages at which key milestones were reached. For each feature, we fit the following model:

$$Y \sim s(\text{Age},\, k = 10) + \text{Sex} \qquad (4)$$

Example GAM fits are shown for the left primary auditory cortex mean T1w/T2w (**Supplementary Figure 11A**), weighted degree (**Supplementary Figure 11B**), and an example edge (**Supplementary Figure 11C**). To quantify the rate of change across the lifespan, we computed the first derivative of the fitted curve using the method of finite differences using R's *gratia* package (Simpson 2024). Age ranges in which the confidence intervals of the first derivative do not contain 0 can be thought to be significantly changing (Simpson 2018). First derivative curves are shown for left primary auditory cortex mean T1w/T2w (**Supplementary Figure 11D**), weighted degree (**Supplementary Figure 11E**), and an example edge **(Supplementary Figure 11F**), with significantly changing age ranges highlighted. We defined the maximum age bound of the first significantly changing age range as the age of plateau for that feature. For each feature set, the distribution of age at plateaus is shown in **Supplementary Figure 11G-I**. Regional mean T1w/T2w values plateau before 3 years of age (median age = 2.29), while MIND weighted degree and edge weights continue changing beyond 3 years, though the median age at plateau in both cases is approximately 2.5 years. Significant plateauing in edge-wise age-related changes may therefore contribute to the qualitative plateau in edge-wise correlations between age-binned networks in **Figure 5A**.

Justifying a linear modelling approach

We only had access to *MBP* expression data from two timepoints (pre-adolescence: 6 months and adulthood: 4 years). To fairly compare developmental changes in *MBP* expression and mean T1w/T2w or T1w/T2w MIND, we opted to model T1w/T2w changes as linear. Given that these changes are likely to be non-linear in reality, we see this modelling approach as a necessary simplification to facilitate a fair comparison with *MBP* expression changes.

To statistically assess whether features were best fit by linear or non-linear models between pre-adolescence and 3 years, the age at which regional T1w/T2w values have significantly stopped increasing (**Supplementary Figure 11G**), we used the model specified in (3), which allowed us to parse linear and non-linear effects. Although most regional mean T1w/T2w trajectories were best fit by non-linear models (**Supplementary Table 4**), MIND network properties were more equally-well fit by linear and non-linear models. This suggests that while linear trajectories may be a useful simplification of true underlying T1w/T2w trajectories, they more faithfully capture true MIND network trajectories.

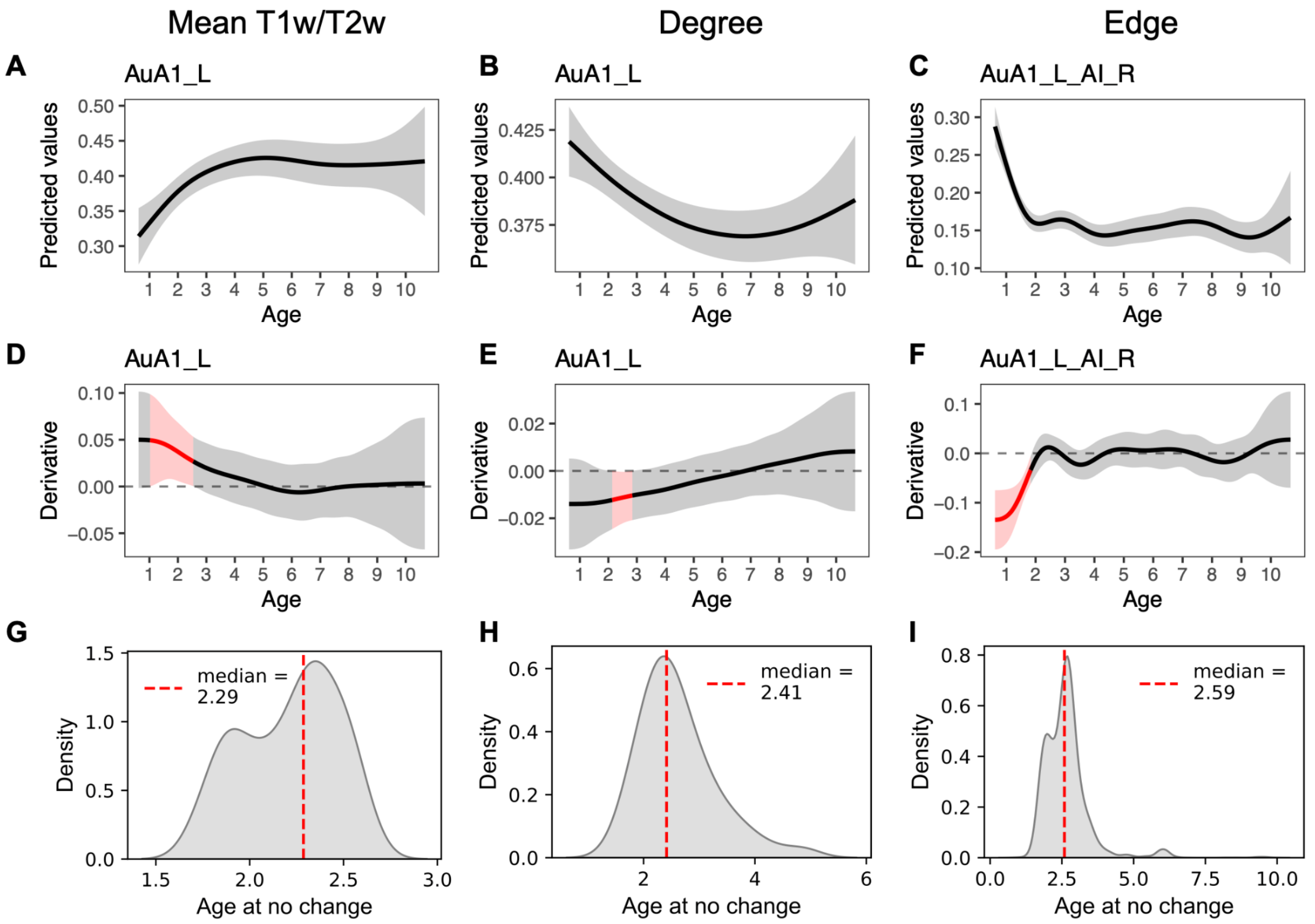

Supplementary Figure 11: Feature values commonly plateau between 2 and 2.5 years. *Example GAM fits for:* ***A*** *the left primary auditory cortex (AuA1_L) mean T1w/T2w,* ***B*** *AuA1_L weighted degree and* ***C*** *MIND edge weight between AuA1_L and the right agranular insula (AI_R).* ***D-F*** *First derivative curves for the GAM model fits in* ***A-C*** *with significantly changing age ranges highlighted in red.* ***G-H*** *Distributions of plateau ages for each set of features. Median plateau age is highlighted in red.*

Feature	% features best fit by linear age term	% features best fit by non-linear age term	% features with no significant age-related change
Mean T1w/T2w	3	90	7
MIND degree	15	30	55
MIND edges	26	20	54

Supplementary Table 4: Features best fit by linear and non-linear models*. Percentage of features best fit by models with a linear age term, non-linear age term, or showing no significant age-related change between pre-adolescence and adulthood.*

Updated **Results**:

"We modelled age-related changes in a subset of N=211 marmosets with ages ranging from 7 to 36 months (pre-puberty to mature adulthood; **Figure 4A**). We used multiple linear regressions to estimate the linear slope of mean T1w/T2w change over this age range (**Figure 4B**). We used linear models because they are simple and easily

interpretable, and this approach allowed a direct comparison with linear age-related changes in *MBP* for which only two timepoints were available. Supplementary non-linear analyses revealed that mean T1w/T2w values plateau before 3 years of age, motivating the use of 3 years as an upper age range for linear analyses (**Supplementary Figure 11**)."

Updated **Discussion**:

"...we used linear modelling approaches as an interpretable way to model age-related changes and to facilitate cross-modal comparisons with *MBP* expression data, for which we only had access to two timepoints, although we expect that cortical myelination similarity would show non-linear trajectories."

2.4

"3. Interpretation of Auditory Cortex Differentiation

- The finding that the Auditory Cortex (AUD) shows a significant decrease in nodal degree (myeloarchitectonic differentiation) during adolescence is compelling. While this may suggest functional specialization related to vocal communication, the observed structural differentiation should also be interpreted in the context of the AUD's high susceptibility to experience-dependent plasticity throughout life, particularly in the common marmoset.
- Current literature suggests that the marmoset auditory cortex:
  - Undergoes significant experience-dependent reorganization based on long-term exposure to self-generated vocalizations (Chuang et al., 2005).
  - Exhibits a high degree of vocal plasticity during development, which is tied to social interaction and environmental cues (Gultekin et al., 2021).
  - Displays dynamic, context-dependent modulation (facilitation/suppression) of neural responses based on the immediate sound environment (Wang et al., 2025).
- Please discuss the AUD's structural differentiation as a potential reflection of its enhanced sensitivity and vulnerability to environmental/social influences. The high plasticity in AUD may lead to increased inter-individual variability in its myelin distribution pattern, causing it to become less similar to the average cortical pattern and thus resulting in the observed decrease in MIND Degree (structural divergence). This interpretation directly complements the Discussion's existing argument regarding the enhanced adult plasticity in higher-order areas leading to increased inter-individual variability."

We thank the reviewer for this insight, which we have used as a basis for expanding our discussion on the meaning of adolescent auditory myeloarchitectonic differentiation:

"We observed marked decreases in the weighted degree of auditory cortical regions, indicating that these regions became increasingly myeloarchitectonically differentiated from the rest of the cortex during adolescence and early adulthood. Previous research

in marmosets has demonstrated that vocal behaviours show protracted development, remaining plastic in adulthood (Gultekin et al. 2021), and that the response properties of auditory cortical neurons to self-vocalisations change following manipulation of the laryngeal apparatus in adults (Cheung et al. 2005). This sustained experience-dependent plasticity may contribute to the increasing structural differentiation we observed, as individual vocal and social experiences shape local microstructural properties over time. We also observed myeloarchitectonic differentiation in regions functionally connected to auditory cortex within a broader auditory processing network, particularly the anterior cingulate cortex (Grisjeels et al., 2023; Jafari et al., 2023), suggesting that experience-dependent differentiation may extend beyond primary auditory fields to distributed circuitry supporting vocal communication.

Edge-level similarity network changes were organised along an axis from primary sensory to transmodal association areas (Mesulam 1998; Sydnor et al. 2021)."

We also updated the **Results** to reference the decreasing weighted degree of some frontal areas:

"Anterior temporal, mainly auditory, and some frontal areas showed significant degree decreases…"

2.5

"4. Reproducibility concerns
Please clarify the definition of the cortical network nodes. Specifically, were the cortical regions defined bilaterally (e.g., Left V1 and Right V1 as separate nodes), or was the analysis restricted to a single hemisphere? This is vital for interpreting network topology."

We apologise for our lack of methodological clarity, which we have addressed in the updated **Methods** section:

"Cortical areas were defined with separate left and right counterparts. For a full list of cortical areas used in our analyses, see **Supplementary Table 1**. The full preprocessing pipeline is visualised in **Supplementary Figure 1**."

2.6

"5. Figure
There was no description about Figure 3E. The description about 3E seemed to be included in 3D."

We thank the reviewer for noting this error. The **Results** and **Figure 3** legend in the updated manuscript have been amended:

"We observed that both T1w/T2w MIND and *MBP* MIND were most strongly correlated with whole genome co-expression by PV+ and vasoactive intestinal peptide (VIP+) interneuronal subtypes (**Figure 3D-E**).

…

D *T1w/T2w MIND was correlated with gene co-expression in PV+ and VIP+ interneurons, though no correlations were significant after correction for multiple comparisons.* ***E*** *MBP MIND was strongly and significantly correlated with gene co-expression in PV+ and VIP+ interneurons*."

Reviewer 3

3.1

"Summary and General Assessment:
This paper investigates MRI-derived structural similarity in the common marmoset brain using Kullback–Leibler (KL) divergence and examines its correspondence with multiple forms of biological data—including gene expression patterns, single-cell transcriptomic profiles, and tracer-based cytoarchitectural information—sourced from publicly available databases. The approach is promising for advancing our understanding of primate architectomics, with potential relevance to human brain research, and it highlights the utility of large-scale, data-driven methodologies. However, the clarity of the manuscript limits its overall impact. Substantial revision of the narrative structure and presentation would improve readability and strengthen the contributions of the study."

We thank reviewer 3 for their feedback and apologise for any lack of clarity. We have substantially revised the manuscript throughout for greater clarity.

3.2

"Major Comments:
1. Lines 211–215 and Figure 1C: Is there a reason the manuscript does not report a p-value for the correlation between Euclidean distance and MINDS similarity, while significance is reported for the correlations with T1w/T2w similarity and axonal connectivity? Does this omission imply that the association was not significant? Please clarify and, if applicable, provide the corresponding statistical results."

We thank the reviewer for bringing this to our attention. This p-value was significant but was inadvertently omitted from the relevant text and figure legend. We have added the p-value to the updated **Results** section and **Figure 1C**:

"MIND similarity was negatively correlated with the Euclidean distance between regions (Spearman r = -0.27, $p_{rand} < 0.001$, **Figure 1C**)."

3.3

"2. It would be more appropriate to compare age-matched measures—specifically, the T1w/T2w averages for 4-year-old animals and the MBP map derived from 4-year-old data rather than averaging across all ages. Using age-specific values would yield a more accurate interpretation of the relationship between myelin proxies and MBP expressions."

We thank the reviewer for raising this important point. When repeating the analyses in **Figure 2** using age- and sex-matched animals, correlations and p-values were highly stable. We report these statistics in the updated manuscript (see section **1.11**).

3.4

"3. Please clarify why the analysis of age-related changes was restricted to developmental and young-adult ages, rather than including the full dataset, which contains older individuals (>8 years). The rationale for defining the developmental period as up to 36 months is unclear. Prior literature typically defines sexual maturity around 18–24 months, and the MBP datasets used by the authors involve animals at 0.6 and 4 years of age. If the assumption is that T1w/T2w values plateau after approximately 3 years of age, a nonlinear regression analysis would be more appropriate to test this hypothesis. If the intention is to focus specifically on developmental processes, it may be clearer to frame the analysis as "age-related developmental change" rather than simply "age-related change." "

We thank the reviewer for raising this critical issue. In the **Supplementary Methods**, we now hypothesise that increases in T1w/T2w ratio may continue beyond the accepted onset of sexual maturity in marmosets, as is the case in humans (Grydeland et al. 2019), and undertake non-linear analyses of T1w/T2w across the lifespan to test this hypothesis. These analyses demonstrate that regional mean T1w/T2w values continue changing after 18-24 months, stopping by 36 months (see section **2.3**). We also provide additional clarification for our continued use of linear models between 7 and 36 months in downstream analyses to enhance interpretability and facilitate comparisons with *MBP* expression data (see section **2.3**).

3.5

"Minor Comments:
1. In addition to the MIND similarity heatmap, providing region-wise KL divergence maps—particularly for comparisons between primary sensory and prefrontal association cortical zones, where the authors report strong dissimilarity—would greatly enhance and clarify the interpretability of the results."

We thank the reviewer for this interesting suggestion for additional analysis, which we have done and now report in the **Supplementary Information** and **Results**:

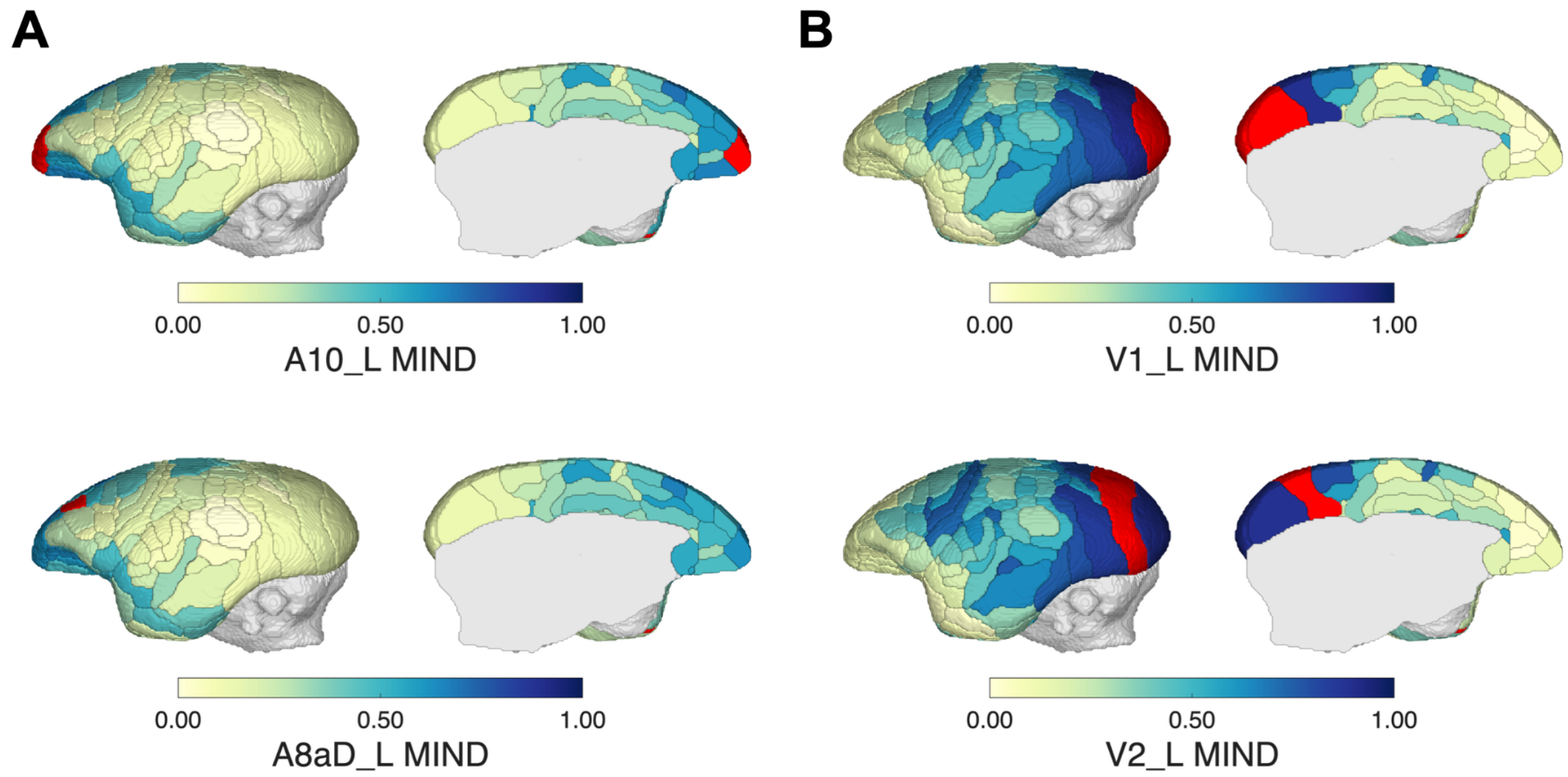

Supplementary Figure 5: Seed-based MIND maps for the mean adult T1w/T2w MIND network. *Each map shows the MIND similarity between a seed region, highlighted in red, and other cortical regions. The MIND network was highly symmetric, so only left hemispheric regions are shown, for simplicity. Example seeds were chosen from two regions in the* ***A*** *dorsolateral prefrontal cortex and* ***B*** *primary visual cortex to exemplify how patterns of similarity tend to diverge between association and primary sensory regions.*

Updated **Results**:

"The average adult (N = 340, mean age = 4.3 years) T1w/T2w similarity network displayed high average similarity within anatomically and functionally defined cortical zones (Paxinos et al. 2012) and strong dis-similarity between primary sensory and prefrontal association cortical zones (**Figure 1B**). For two example primary sensory and prefrontal association regions, seed-based maps of MIND similarity to the rest of the cortex are visualised in **Supplementary Figure 5**."

3.6

"2. Please include the term "cell type–specific correlated gene expression (CGE)" in the legend of Figure 3C, as it does not appear in the manuscript until line 866."

We thank the reviewer for drawing our attention to this lack of consistency in our terminology. We have amended this in the updated **Figure 3A** legend (where CGE matrices are first referenced):

"**A** Cell type-specific correlated gene expression (CGE) matrices estimated using single nucleus RNA sequencing data from 12 cortical areas (Krienen et al. 2023)"

3.7

"Technical Comments:
1. Lines 708–710: I guess the T1w image matrix in native space does not match that of the T2w image (T1w is acquired in 3D, whereas T2w is acquired in 2D). Consequently, the voxel positions in T1w do not correspond to those in T2w. How, then, was the T1w/T2w ratio computed without performing co-registration? Clarification on this procedure is needed."

We thank the reviewer for this important question. Co-registration was not performed because: i) reconstructed T1w and T2w image volumes were provided on identical native-space sampling grids and ii) marmosets were head-fixed and anesthetised between acquisitions, minimising head motion. The reviewer is correct to point out that point i) was not sufficiently clarified in the original manuscript. We have amended this in the **Methods**:

"Reconstructed T1w and T2w images had identical matrix dimensions (178 × 178 × 96), voxel sizes (0.27 mm isotropic), orientations (RAS), and voxel-to-RAS affine transformation matrices, indicating a one-to-one correspondence of voxel coordinates across modalities.

…

T1w and T2w images were not co-registered prior to calculation of T1w/T2w images because: i) reconstructed T1w and T2w image volumes were provided on identical native-space sampling grids; and ii) marmosets were anaesthetised and head-fixed before scanning, minimising head motion between T1w and T2w image acquisitions (Hata et al. 2023).

3.8

"2. The ISH volume appears to be missing occipital areas, as noted in Lines 828–830. How was the information from "at least one representative tissue sample analysable" registered and transformed into MRI space under these circumstances? The lack of occipital coverage raises concerns regarding the validity of the MINDS-derived MBP estimates in those regions."

We apologise for the lack of clarity in the original manuscript. Due to the physical constraints of tissue slicing, some peripheral slices from the occipital (mainly the primary visual cortex, V1) and frontal lobes (mainly the frontal pole, A10) were omitted. However, these regions were contained in a sufficient number of histological slices for them to be representatively sampled in the final *MBP* volume. For example, in the left hemisphere of the 4-year-old *MBP* volume, 43 coronal slices contained area V1 and 22 coronal slices contained area A10. In the left hemisphere of the 6-month-old *MBP* volume, 36 coronal slices contained area V1 and 29 coronal slices contained area A10 in the left hemisphere. We have updated the text in the **Methods** to more clearly illustrate this point:

"Though the posterior pole of occipital cortex and the anterior pole of frontal cortex were not completely covered, each brain region in the Paxinos et al. (2012)

parcellation was present in a sufficient number of slices to constitute a representative sample of brain tissue for analysis at both ages."

3.9

"Typographical and Grammatical Errors:
1. The font size in several sections (e.g., Lines 208–223 and the legend of Supplementary Figure 6) appears smaller than the surrounding text. Please ensure consistent formatting throughout the manuscript."

We thank the reviewer for pointing out these inconsistencies. We have ensured that font size and font family are consistent throughout the manuscript text. Any inconsistencies in word spacing (for example in **Data Availability**) are due to our use of Microsoft Word's "Justify text" to ensure that text spans the entirety of each line.

3.10

"2. Line 314: Although not a typographical error, it would be clearer to specify "T1w/T2w MIND" rather than simply "MIND," to distinguish it from the MBP-derived MIND measure."

This has been amended in the updated manuscript:

"The cell type-specific correlation between whole genome co-expression and T1w/T2w MIND similarity was strongest for glutamatergic neurons"

3.11

"3. Line 334: "Figure 3B–C" appears to refer instead to Figures 3D–E. Line 338: "Figure 3D" may also need to be updated to "Figure 3D–E." Please verify and correct the figure references accordingly."

We thank the reviewer for drawing our attention to these issues. Line 334 does intentionally refer to **Figure 3B-C** to convey that the analyses in **Figure 3D-E** were performed in the same way. To prevent confusion, we have removed this bracketed phrase. We have also correctly referenced **Figure 3D-E**:

"We therefore estimated the correlations between interneuronal subtype-specific whole genome co-expression and T1w/T2w MIND edge weights ~~(as in **Figure 3B-C**)~~ for a set of 10 interneuron subtypes defined a priori by hierarchical clustering (Krienen et al. 2023, **Supplementary Figure 10**). We observed that both T1w/T2w MIND and *MBP* MIND were most strongly correlated with whole genome co-expression by PV+ and vasoactive intestinal peptide (VIP+) interneuronal subtypes (**Figure 3D-E**)."
